# The Use of Comparative Genomic Analysis for the Development of Subspecies-Specific PCR Assays for *Mycobacterium abscessus*

**DOI:** 10.3389/fcimb.2022.816615

**Published:** 2022-03-28

**Authors:** Winifred C. Akwani, Arnoud H.M. van Vliet, Jordan O. Joel, Sönke Andres, Margo Diricks, Florian P. Maurer, Mark A. Chambers, Suzanne M. Hingley-Wilson

**Affiliations:** ^1^ Department of Microbial Sciences, School of Biosciences and Medicine, Faculty of Health and Medical Sciences, University of Surrey, Guildford, United Kingdom; ^2^ Department of Pathology and Infectious Diseases, School of Veterinary Medicine, Faculty of Health and Medical Sciences, University of Surrey, Guildford, United Kingdom; ^3^ National and Supranational Reference Center for Mycobacteria, Research Center Borstel, Borstel, Germany; ^4^ German Center for Infection Research (DZIF), Partner Site Hamburg-Lübeck-Borstel-Riems, Hamburg, Germany; ^5^ Molecular and Experimental Mycobacteriology, Research Center Borstel, Borstel, Germany; ^6^ Institute of Medical Microbiology, Virology, and Hygiene, University Medical Center Hamburg-Eppendorf, Hamburg, Germany

**Keywords:** *Mycobacterium abscessus*, *Mycobacterium bolletii*, *Mycobacterium massiliense*, multiplex polymerase chain reaction, cystic fibrosis, genomics

## Abstract

*Mycobacterium abscessus* complex (MABC) is an important pathogen of immunocompromised patients. Accurate and rapid determination of MABC at the subspecies level is vital for optimal antibiotic therapy. Here we have used comparative genomics to design MABC subspecies-specific PCR assays. Analysis of single nucleotide polymorphisms and core genome multilocus sequence typing showed clustering of genomes into three distinct clusters representing the MABC subspecies *M. abscessus*, *M. bolletii* and *M. massiliense*. Pangenome analysis of 318 MABC genomes from the three subspecies allowed for the identification of 15 MABC subspecies-specific genes. *In silico* testing of primer sets against 1,663 publicly available MABC genomes and 66 other closely related *Mycobacterium* genomes showed that all assays had >97% sensitivity and >98% specificity. Subsequent experimental validation of two subspecies-specific genes each showed the PCR assays worked well in individual and multiplex format with no false-positivity with 5 other mycobacteria of clinical importance. In conclusion, we have developed a rapid, accurate, multiplex PCR-assay for discriminating MABC subspecies that could improve their detection, diagnosis and inform correct treatment choice.

## Introduction

The *Mycobacterium* (*Mycobacteroides*) *abscessus* complex (MABC) consists of a group of rapidly growing, nontuberculous mycobacteria (NTM) that are associated with pulmonary infections, especially in cystic fibrosis patients (Harris et al., 2012), and has become one of the most common groups of NTM species isolated from pulmonary samples worldwide (Hoefsloot et al., 2013). MABC infections have limited chemotherapeutic options due to both intrinsic and acquired drug resistance to the most commonly used antibiotic classes (Haworth et al., 2017). Despite the prolonged treatment with a combination of different antibiotics (including macrolides, rifampicin and ethambutol), MABC infections remain difficult to treat and have poor treatment outcomes (Chen et al., 2019). These infections pose a major challenge to public health.

MABC has been subdivided into three independent subspecies: *M. abscessus* subsp. *abscessus* (*M. abscessus*)*, M. abscessus* subsp. *bolletii* (*M. bolletii*) and *M. abscessus* subsp. *massiliense* (*M. massiliense*) (Leao et al., 2011). MABC subspecies can exhibit both smooth and rough morphotypes which is influenced by the cell wall glycopeptidolipid (GPL). It is believed that the morphotypes contribute to bacterial colonization and play a role in virulence, with the rough strains generally being more virulent compared with the smooth strains (Howard et al., 2006; Kim et al., 2013; Bernut et al., 2016). These subspecies differ in treatment options and their potential clinical outcomes. For example, the three subspecies differ in their susceptibility to macrolide antibiotics, which can be ineffective when treating MABC infections (Davidson et al., 2014). This is due to the presence of an inducible functional erythromycin resistance methylase (*erm*41) gene that confers macrolide resistance in both *M. bolletii* and in a subpopulation of *M. abscessus* but is non-functional and non-inducible in *M. massiliense* (Bastian et al., 2011; Shallom et al., 2013; Brown-Elliott et al., 2015; Kim et al., 2015). Thus, correct identification of MABC subspecies is of high clinical relevance.

Classical mycobacterial identification methods have been based on the phenotypic characterisation of isolates following culture, including medium selectivity, colony morphology, growth rate (less than seven days), pigmentation and biochemical tests (positive arylsulfatase test, negative nitrate reductase, negative iron uptake, positive sodium chloride tolerance and negative citrate utilisation) (McGrath et al., 2008; Leao et al., 2009; Bhalla et al., 2018). The outcome of these tests can take up to two weeks (Bhalla et al., 2018). While these are often considered the gold standard, they are dependent on bacterial growth and often take a long time, particularly with slow growing mycobacterial species (more than seven days).

The three subspecies of *M. abscessus* are often considered to be phenotypically indistinguishable (Leao et al., 2009), and hence alternative methods are required. Molecular techniques and mass spectrometry methods have largely replaced biochemical techniques for the identification of MABC isolates. Line probe assays (GenoType NTM-DR) have been used to identify MABC to the subspecies level and determine antibiotic resistance to macrolides (Kehrmann et al., 2016). Similarly, multi-locus sequence typing (MLST) and multispacer sequence typing (MST) have been used (Sassi et al., 2013; Wuzinski et al., 2019), but are time-consuming and expensive, while mass spectrometry-based methods such as Matrix-assisted laser desorption/ionization-time of flight (MALDI-TOF) require the use of expensive equipment and trained personnel (Fangous et al., 2014; Panagea et al., 2015).

PCR-based tests offer a rapid and relatively simple alternative, which have been able to differentiate between *M. abscessus* and *M. massiliense*, but not *M. bolletii* (Nakanaga et al., 2014; Mase et al., 2019). However, there is a limited number of PCR-based methods that have been developed which are able to differentiate between all the three subspecies (Minias et al., 2020; Yoshida et al., 2021).

Rapid developments in genome sequencing have resulted in the availability of a large number of pathogen genomes, which are invaluable for our understanding of the evolution, epidemiology and biology of these microbial pathogens. In this study, we have used the wealth of genomic information available for the subspecies of MABC to study the genetic differences between the subspecies, and to identify genes specific to each. These subspecies-specific genes have subsequently been used to design a subspecies-distinguishing PCR assay that can be further developed for use in clinical diagnostics to aid treatment choice.

## Materials and Methods

### Bacterial Strains and Growth Conditions

*M. abscessus, M. bolletii*, and *M. massiliense* clinical isolates were a kind gift from the National *Mycobacterium* Reference Laboratory, Borstel, Germany. An auxotrophic mutant strain of *M. tuberculosis* (H37Rv Δ*panCD* Δ*leuCD*) was used as a control (Vilchèze et al., 2018). Mycobacterial strains were grown on Middlebrook 7H11 agar (Sigma-Aldrich) supplemented with 0.5% glycerol and 10% oleic albumin dextrose catalase (OADC) (Becton Dickinson (BD), Oxford, UK), and incubated at 37°C for 3 to 5 days for MABC strains and, 14 to 21 days for *M. tuberculosis*. *E. coli* BW25141 was grown on LB agar (Sigma-Aldrich) and used as a further control.

### Genomic DNA Isolation and Genome Sequencing

Genomic DNA of non-MABC NTM species (*M. chelonae, M. avium, M. intracellulare* and *M. chimaera*) were also a kind gift from National *Mycobacterium* Reference Laboratory, Borstel, Germany. Genomic DNA was extracted from MABC isolates and from *M. tuberculosis* using a protocol specific for mycobacteria, including enzymatic digestion, mechanical disruption of the cell wall and extraction with phenol/chloroform/isoamyl alcohol 25:24:1 (Käser et al., 2010). Genomic DNA of *E. coli* was extracted using Monarch Genomic DNA purification kit (New England Biolabs, Ipswich, MA, USA). Genomic DNA was quantified using a Biodrop µLITE spectrophotometer (ISOGEN Life Science, Netherlands). The integrity of the genomic DNA samples was assessed by the presence or absence of a dense molecular band using agarose gel electrophoresis.

### Genome Sequence Selection

**Figure 1:**
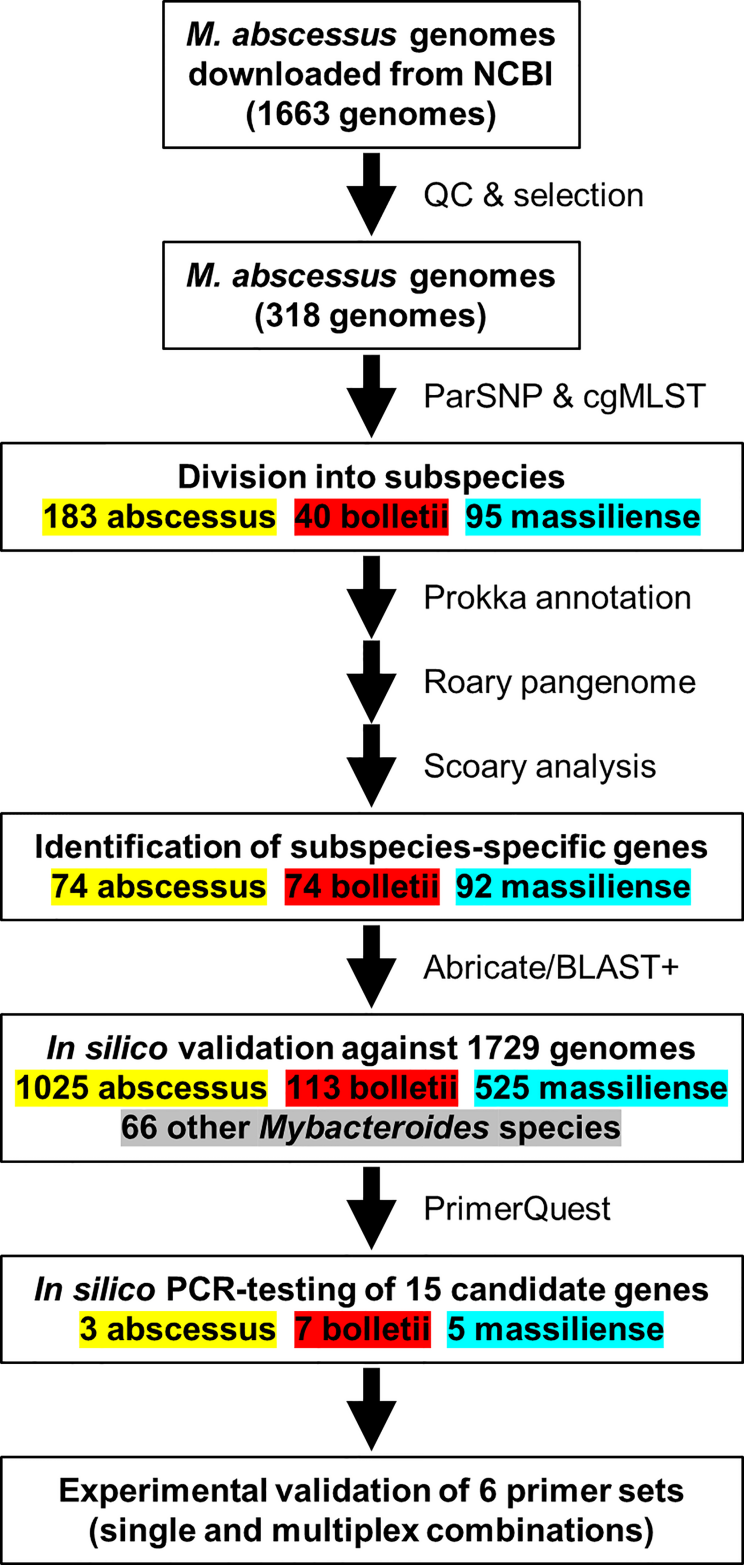
An overview of the workflow used to identify *M. abscessus* genomes for the *in-silico* evaluation of the PCR primers sequences.

A total of 1,663 MABC genome sequences and 66 genome sequences from related *Mycobacterium* species (e.g., *M. chelonae*, *M. immunogenem*) were obtained from GenBank using NCBI-genome-download version 0.2.3 (https://github.com/kblin/ncbi-genome-download) (**Supplementary Table S1**). These were first assessed for assembly quality using QUAST version 4.6.3 (Gurevich et al., 2013). Exclusion criteria were the presence of N’s in the contigs, N50<25 kb, L50>50, number of contigs >300 and absence of information on isolation source. The final dataset for pangenome analysis consisted of 318 genomes, whereas the excluded genome sequences were used to test the specificity of selected genes from pangenome analyses *in silico* and for *in silico* PCR assays (**Figure 1**).

### Bioinformatic Analysis of Genome Sequences

Phylogenetic analysis of the sub-selection of 318 MABC genomes (**Supplementary Table S1**) was based on core genome single nucleotide polymorphisms identified using ParSNP with the “-a 13 -x” settings as described elsewhere (Pornsukarom et al., 2018). Isolates were assigned to the three MABC subspecies based on their placement on the core genome SNP and core genome MLST phylogenetic trees. Genomes were subsequently annotated using Prokka version 1.14 (Seemann, 2014). The GFF files produced were used to create the pangenome of MABC using Roary 3.12 (Page et al., 2015) using default settings and a BLAST cut-off of 95%, and subspecies-dependent genes were identified using Scoary (Brynildsrud et al., 2016), with an initial threshold of a Bonferroni-corrected p-value of 0.05. Subsequently genes were only included if present in >90% of their respective subspecies, and <10% of the other subspecies. Selected genes were tested against all 1,663 MABC genomes and 66 related *Mycobacterium* spp using Abricate version 1.0.9 (https://github.com/tseemann/abricate) with a minimum coverage of 70% and minimum identity of 80% for a positive match.

### Core Genome Multilocus Sequence Typing (cgMLST)

A core genome MLST scheme was developed for MABC subspecies using chewBBACA version 2.8.5 (Silva et al., 2018) with default settings, as described in the tutorial on the program GitHub webpage (https://github.com/B-UMMI/chewBBACA_tutorial). A training file for *M. abscessus* was generated from the completed genome of *M. abscessus* subspecies *abscessus* UC22 (Accession number CP012044.1) using Prodigal version 2.6.3 (Hyatt et al., 2010). The scheme was initially generated using 18 genomes of each subspecies, selected on genomes being complete or if draft, to have a high N50, low L50, and spread over the core genome SNP-tree. An initial whole genome MLST scheme contained 10,913 loci, and a subset of 2,785 loci present in >99% of the 54 genomes was selected for further screening with the 318 MABC subspecies used for the core genome SNP tree, and the other 1,345 MABC genomes (**Supplementary Table S1**). This allowed the scheme to be further improved in a selection of 1,262 genes with a chewBBACA threshold of 25, and a more selective scheme with 882 genes (chewBBACA threshold of 50). Phylogenetic trees from the chewBBACA allele calls were constructed using GrapeTree version 1.5.0 and the RapidNJ algorithm (Zhou et al., 2018). The MABC subspecies cgMLST scheme with allele sequences and training file is available from FigShare (https://doi.org/10.6084/m9.figshare.19158563.v1) and will be made available *via* Chewie-NS (https://chewbbaca.online) (Mamede et al., 2021).

### PCR Amplification Primers and Parameters

Based on the final candidate genes chosen, primers were designed using PrimerQuest (Integrated DNA Technologies, https://eu.idtdna.com/pages/tools/primerquest). The amplicon size range was between 100-200 bp with an optimum length set at 125 bp. The primers are detailed further in **Table 1**. Single blind and multiplex PCR assays differentiating MABC into its subspecies (*M. abscessus*, *M. bolletii* and *M. massiliense*) were conducted with the listed primers. The total bacterial DNA samples were numbered from one to fifteen including all the controls by another member of the laboratory for a single blinded test. This blinded test was carried out from the beginning of the DNA extraction using the procedures detailed above.

**Table 1:** Selected genes, product sizes and primer sequences for *M. abscessus* subspecies-specific PCRs.

Reference genome^*^	Gene number	Coordinates	Primers	Product
*M. abscessus* UC22	MAUC22_07270	3729832-992	5’-TCCAACCGAGATGACCAGAG	161 bp
			5’-CCGATATAGAATTCGGCCAGCAAGT	
*M. abscessus* UC22	MAUC22_18635	1444138-323	5’-CCATCACCACACAAGGAGAG	186 bp
			5’-CCAAAGACTCGCGCAACAATC	
*M. bolletii* BD	MASB_RS22045	4413042-217	5’-GGCTTCACGTTCAATCAGTTTCTA	176 bp
			5’-CGATTCACTGCTCCGCATTC	
*M. bolletii* BD	MASB_RS03355	671265-448	5’-GTTGTAGGGATGACGTGGTG	184 bp
			5’-CTCCGCACCGAAGAAGAAAT	
*M. massiliense* JCM15300	MMASJCM_0836	828761-894	5’-AGGGTATTTCACTTGATGACCTATG	134 bp
			5’-GATCGCCGTCAGCGAATAAT	
*M. massiliense* JCM15300	MMASJCM_0834	826228-365	5’-GTCAGCAACTCGGCAAGAAG	138 bp
			5’-GTTTCTCCTGGAACGAGATCTAATG	

*The accession numbers of the genome sequences used are M. abscessus UC22: CP012044, M. bolletii BD: NZ_AP018436, and M. massiliense JCM15300:AP014547.

For the single PCR assays, each mixture contained 12.5 µl of 2 × GoTaq G2 Green master mix [which comprises of Buffer (pH 8.5), 400 µM of each of the dNTP and 4 mM MgCl_2_] (Promega), 0.4 µM of the forward primer and 0.4 µM of the reverse primer, 1 µl of the extracted DNA [diluted, DNA concentration (1-10µg)], 1 µl of DMSO and nuclease free water to achieve a final volume of 25 µl. A negative template control that consisted of each primer and 1 µl of nuclease free water was also included. For the multiplex PCR assays, each mixture contained 25 µl of 2× GoTaq G2 Green master mix (Promega), 0.2 µM of each of the forward primers and 0.2 µM of each of the reverse primers of all the MABC subspecies primers sets, 1 µl of all the extracted DNA and controls (DNA concentration <5µg), 2 µl of DMSO and nuclease free water to achieve a final volume of 50 µl. Amplification was carried out in a thermal cycler (Applied Biosystems™ SimpliAmp™ Thermal Cycler, Fisher Scientific, Loughborough, UK) using the following PCR cycling conditions: initial denaturation at 95°C for 2 min; 35 cycles of denaturation at 95°C for 1 min; annealing at 60°C for 1 min; and extension at 72°C for 1.5 min; with final extension at 72°C for 10 min. The PCR products were separated by 2% agarose gel electrophoresis.

## Results

### Identification of Genomic Diversity in MABC Subspecies-Specific Genes by Pangenome Analysis

A set of 1,663 MABC genomes was obtained from public repositories with their metadata (**Supplementary Table S1**), and genomes were clustered using phylogenetic trees based on core genome single nucleotide polymorphisms (SNPs) and using a core genome multiclocus sequence typing (cgMLST) scheme developed for this study. Both methods resulted in subdivision in three large clusters, representing the three MABC subspecies *M. abscessus*, *M. bolletii* and *M. massiliense* (**Supplementary Figure S1**). Comparison of the SNP- and cgMLST based trees using a tanglegram showed differences of order within each MABC subspecies cluster, but no differences in subspecies assignment (**Supplementary Figure S2**). The initial dataset consisted of 840 *M. abscessus*, 113 *M. bolletii*, 351 *M. massiliense* and 359 genomes where the MABC subspecies was not identified in the description. Of the 359 MABC genomes without subspecies information, 182 were determined to be *M. abscessus*, 18 *M. bolletii* and 159 *M. massiliense*. There were 18 genomes that did not cluster with the named subspecies, suggesting the original description was incorrect (**Supplementary Table S1**); these were all *M. bolletii* genomes which were *M. abscessus* (n=2) and *M. massiliense* (n=16) (**Supplementary Table S1**). In the remainder of this study, we have used the genomic clustering as leading.

### Identification of MABC Subspecies-Specific Genes by Pangenome Analysis

**Figure 2:**
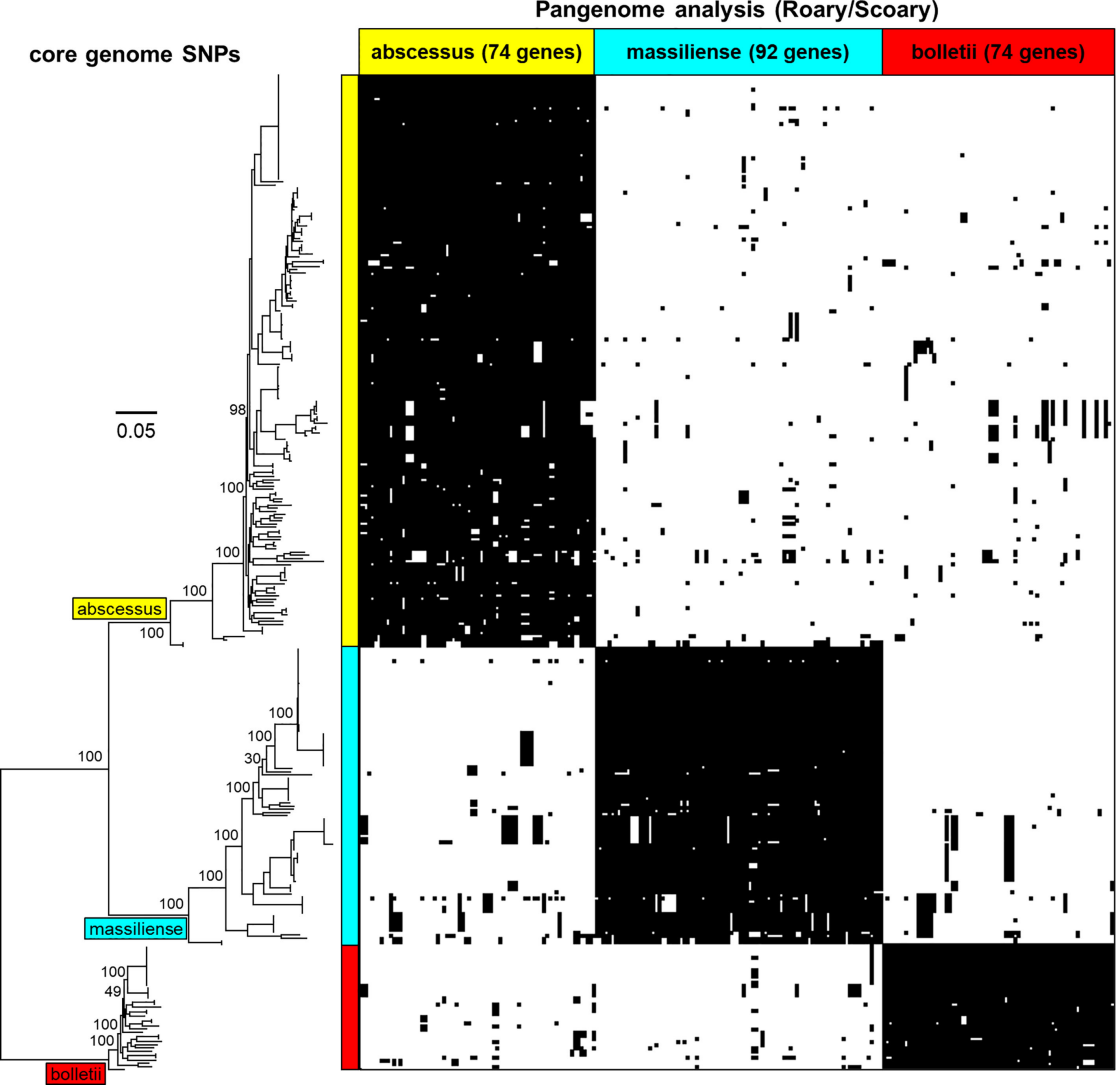
Phylogenetic tree of the core genomes of the MABC subspecies *- M. abscessus* (yellow), *M. massiliense* (light blue) and *M. bolletii* (red) with pangenome matrix of genes specific to MABC. The pangenome was determined using Roary and queried using Scoary based on the core genomes showing subspecies- specific genes that are either present or absent. Bootstrap values based on ParSNP tree iterations (expressed in percentage) have been added to the major branches in the phylogenetic tree.

A selection of 318 MABC genomes (183 *M. abscessus*, 40 *M. bolletii* and 95 *M. massiliense*) was used to perform pangenome analysis using Roary (Page et al., 2015) and were clustered using core genome SNPs (**Figure 2**). The pangenome was interrogated for MABC-subspecies-specific genes using Scoary (Brynildsrud et al., 2016), with subspecies-specific genes defined as being present in >90% of the specific subspecies, and <10% in the other two subspecies. A total of 240 subspecies-specific genes were identified, with 74 being *M. abscessus*-specific, 74 being *M. bolletii*-specific, and 92 being *M. massiliense*-specific (**Figure 2**). These were further tested against the full 1,663 MABC genomes, and a further 66 genomes representing phylogenetically related *Mycobacterium* species to *M. abscessus* (e.g., *M. chelonae*, *M. immunogenem*, see **Supplementary Table S1**) (Gupta et al., 2018). This reduced the number of MABC subspecies-specific genes to three *M. abscessus*-specific, seven *M. bolletii*-specific and five *M. massiliense*-specific genes. The 15 candidate genes were subsequently tested using *in silico* PCR amplification for their MABC subspecies specificity (**Table 2**) with the complete 1,663 MABC genomes and the 66 other *Mycobacteria* genomes. For each of the subspecies, two primer pairs representing two individual genes of each subspecies were selected as they showed >98% specificity and >97% sensitivity in the *in-silico* PCR (**Table 2**, **Supplementary Table S1** and **Supplementary Figure S1**). These primer sets were further tested using conventional PCR.

**Table 2:** *In silico* PCR validation of *M. abscessus* subspecies-specific PCR primers.

Gene number	Genome	Size (bp)	*M. abscessus* subspecies			Other (n=66)^a^
			*abscessus* (n=1024)	*bolletii* (n=113)	*massiliense* (n=526)	
MAUC22_07270	*abscessus* UC22	161	1016 (99.2%)	0 (0%)	3 (0.6%)	0 (0%)
MAUC22_18635	*abscessus* UC22	186	1013 (98.9%)	8 (7.1%)	0 (0%)	1 (1.5%)
MASB_RS22045	*bolletii* BD	176	7 (0.7%)	113 (100%)	0 (0%)	0 (0%)
MASB_RS03355	*bolletii* BD	184	0 (0%)	110 (97.3%)	0 (0%)	0 (0%)
MMASJCM_0836	*massiliense* JCM15300	134	2 (0.2%)	1 (0.9%)	513 (97.5%)	0 (0%)
MMASJCM_0834	*massiliense* JCM15300	138	0 (0%)	0 (0%)	513 (97.5%)	0 (0%)

*The category “Other” consisted of 39 M. chelonae, five M. franklinii, 16 M. immunogenem, two M. salmoniphilum and four M. saopaulense. The one positive other Mycobacteroides genome with the MAUC22_18635 PCR was a M. saopaulense genome (Msao1729). Information on all genomes included can be found in **Supplementary Table S1**.

### Single and Multiplex PCR Assay Differentiating *Mycobacterium abscessus* Subspecies

**Figure 3:**
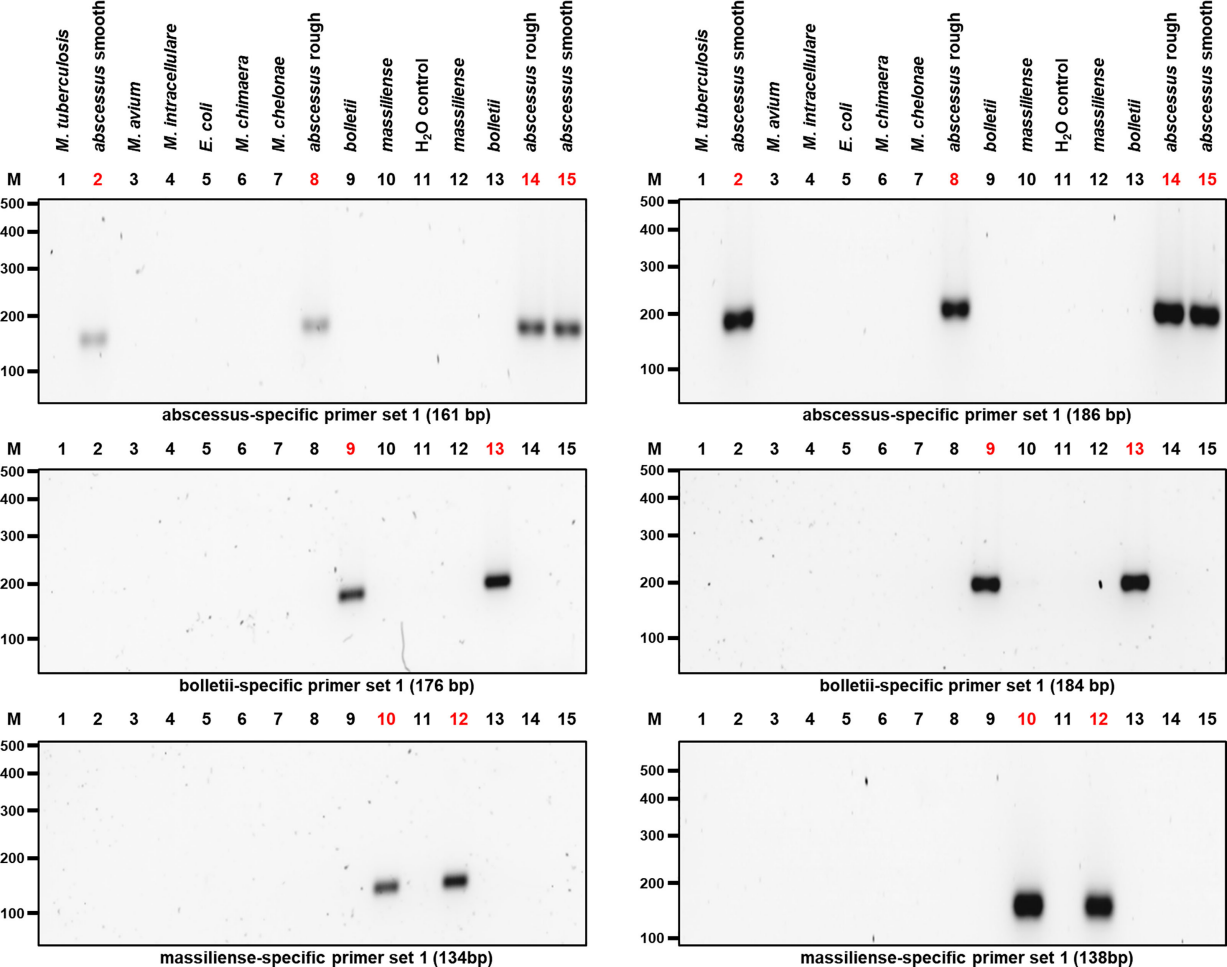
PCR results after optimizing the amplification of the candidate genes used to isolate the three MABC subspecies. Two different sets of primers pairs as shown in **Table 1** were used to differentiate MABC into its subspecies. *M. abscessus* (rough and smooth morphotypes), *M. bolletii*, *M. massiliense* and other bacterial species used as controls including *M. chelonae*, *M. tuberculosis*, *M. avium, M. intracellulare, M. chimaera*, and *E. coli* were selected. Both *M. abscessus* strains including rough and smooth morphotypes yielded 161-bp and 186-bp gene amplicons, respectively (Top Panel). In addition, *M. bolletii* yielded 176-bp and 184-bp gene amplicons, respectively (Middle Panel). Lastly, *M. massiliense* yielded 134-bp and 138-bp gene amplicons, respectively (Bottom Panel). Each MABC subspecies yielded an amplified PCR product that was specific to that species.

The selected primer sets were first tested individually with *M. abscessus*, *M. bolletii* and *M. massiliense* DNA, which resulted in amplicons of the predicted sizes (**Table 1** and **Figure 3**), while negative controls consisting of *E. coli* and other mycobacterial species of clinical importance (*M. chelonae, M. avium, M. intracellulare, M. chimaera, M. tuberculosis*) gave no amplicons.

**Figure 4:**
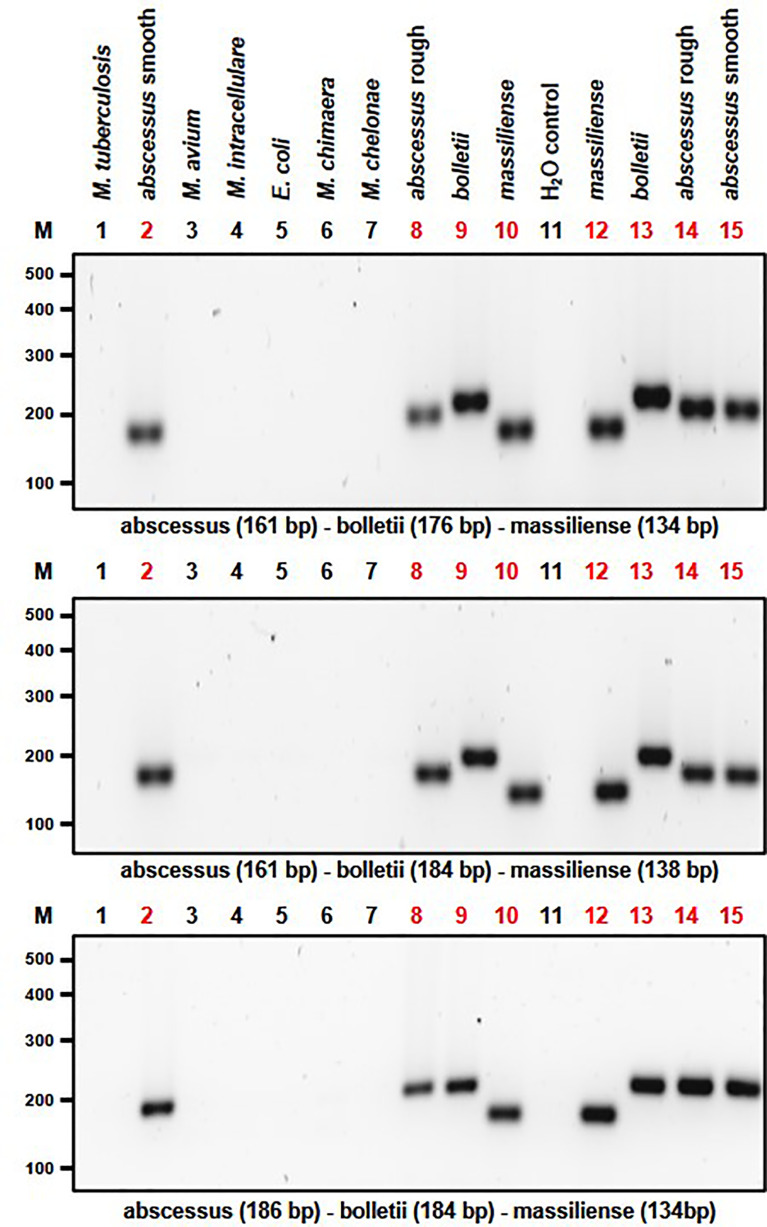
Multiplex PCR assay capable of differentiating MABC subspecies. Multiplex PCR A produced amplicon sizes of 161bp, 176bp and 134bp for *M. abscessus* for both rough and smooth morphotypes, *M. bolletii* and *M. massiliense*, respectively (Top Panel). Multiplex PCR B produced amplicon sizes of 186bp, 184bp and 138bp bands for *M. abscessus* for both rough and smooth morphotypes, *M. bolletii* and *M. massiliense*, respectively (Middle Panel). Multiplex PCR C was able to identify *M. abscessus* for both rough and smooth morphotypes, *M. bolletii* and *M. massiliense* according to their product sizes of 161bp, 184bp and 134bp, respectively (Bottom Panel).

**Figure 5:**
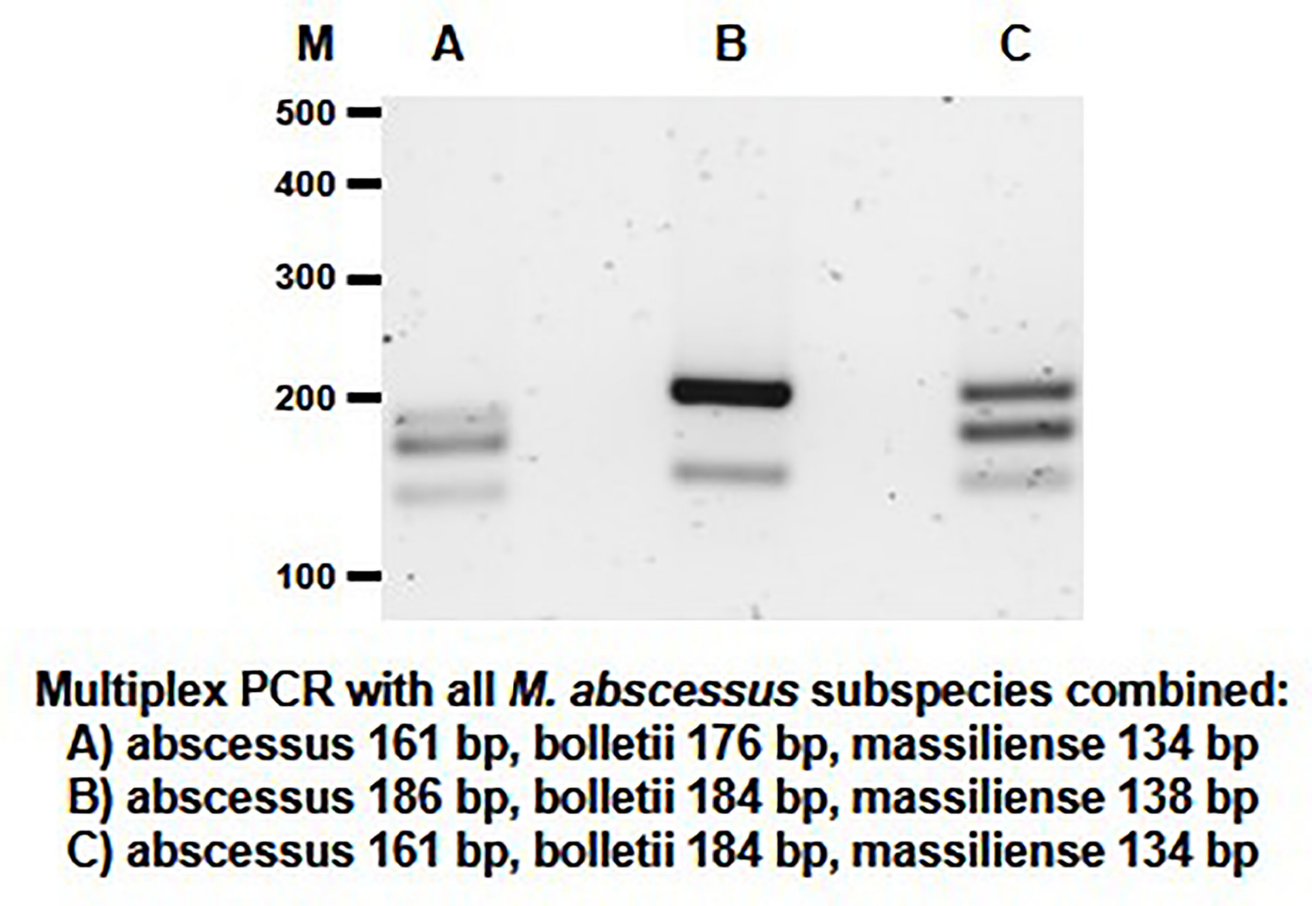
Multiplex PCR assay performed for the detection of the MABC subspecies within a mixed sample. The mixed sample contained the extracted DNA of *M. abscessus*, *M. bolletii*, *M. massiliense* and other bacterial samples (*M. chelonae*, *M. tuberculosis*, *M. avium, M. intracellulare, M. chimaera* and *E. coli)* (controls) in the same tube. The PCR products sizes for the MABC subspecies are shown in the multiplex assays (Lane **A–C)**.

Based on the results of the single PCR assay, we performed multiplex PCR assays using different combinations of the MABC subspecies-specific PCR primers in a multiplex assay. The DNA products were amplified using different combinations of primer pairs (**Table 1** and **Figure 4**). Multiplex PCR A produced PCR products with sizes of 161 bp, 176 bp and 134 bp bands from *M. abscessus*, *M. bolletii* and *M. massiliense*, respectively. Multiplex PCR B produced PCR products with sizes of 186 bp, 184 bp and 138 bp bands for *M. abscessus*, *M. bolletii* and *M. massiliense*, respectively. Finally, multiplex PCR C was able to identify *M. abscessus*, *M. bolletii* and *M. massiliense*, according to their product sizes of 161bp, 184bp and 134bp, respectively. To differentiate *M. abscessus* to the subspecies level, a further multiplex PCR assay was performed that contained a mixture of the individual DNA of all the MABC clinical strains and the controls (*E. coli* and other mycobacterial species of clinical importance) in a tube. The PCR assay accurately identified the three MABC subspecies, without any cross-reactivity detected, despite a mixture of the samples. In lane B it appears to have a single dense molecular band as there is only 2 bp difference (186 vs 184 bp, respectively) between the PCR products for *M. abscessus* and *M. bolletii* (**Figure 5**).

## Discussion

Pulmonary infection with *M. abscessus* is becoming more common worldwide, and this is a significant threat to immunocompromised people, especially cystic fibrosis patients (Degiacomi et al., 2019). Here we have used a pangenome-based approach to to successfully differentiate the three subspecies of MABC isolated from clinical samples. The primary value of this approach is to use the selected genes concurrently to rapidly distinguish the subspecies based on the different sizes of the amplicons. Whilst commercially available diagnostic methods such as MALDI-TOF (Saleeb et al., 2011) and DNA probes [Inno LiPA Mycobacteria (Innogenetics) or GenoType NTM-DR assay (Hain Lifesciences)] (Tortoli et al., 2010) are able to identify *M. abscessus* subspecies, these methods are expensive and have a comparatively longer turnaround time.

Several molecular assays have been developed for the identification of MABC, however, some of these assays have showed inaccuracies in separating the three subspecies. The general molecular target used for differentiation of mycobacterial species is the 16S rRNA gene. However, diagnostic methods based on the 16S RNA sequence are unable to sub-speciate MABC, due to the lack of sequence diversity (Clarridge, 2004). Therefore, alternative genes (*hsp65* and *rpoB*) have been used as targets for the identification of mycobacteria species. It has been reported that based on combinational genotypic analysis of the internal transcribed spacer (ITS) regions, *hsp65* and *rpoB* genes, two of the subspecies (*M. abscessus* and *M. massiliense*) have been separated (Nakanaga et al., 2014). Furthermore, the use of the *rpoB*-based methods has showed that there is an increased risk of misidentification of the MABC due to lateral gene transfer of *rpoB* alleles (Kim et al., 2019).

Phylogenetic approaches have been used for subspecies classification; this has been achieved using sequencing analysis with multiple housekeeping genes (Tan et al., 2013). Nevertheless, this methodology of using multiple gene targets is expensive and time consuming. In addition, it has been reported that *M. massiliense* has been misidentified as *M. abscessus* due to similarity in gene features (Tan et al., 2013). In addition, evolutionary trees have been used to understand the relationship between microevolutionary processes and the diversification of MABC at the subspecies level (Tan et al., 2017). Similarly, another phylogenetic approach used a large dataset of the MABC genomes to understand the relationship between dominant circulating clones and drug resistance evolution in cystic fibrosis and non-cystic fibrosis patients around the world (Bronson et al., 2021). Phylogenetic analysis has been used as an accurate method to classify the subspecies of MABC using whole genome sequencing. Even though whole genome sequencing is the ‘gold standard’ approach for the identification of the MABC, it is an expensive method and there is a need for other cost-effective tools to be developed.

As an alternative of using multiple genes, a single gene target (*gnd*) has been used to identify the three MABC subspecies, however this gene target misidentified *M. massiliense* as *M. abscessus* (Ng and Ngeow, 2020). Due to the presence of an inducible functional erythromycin resistance methylase (*erm*41) gene that confers macrolide resistance within the subspecies, this gene has also been used as a target in identifying MABC. Nevertheless, this target has also showed discrepancies as only two of the three subspecies (*M. abscessus* and *M. massiliense*) have been differentiated (Mase et al., 2019). In addition, some *M. massiliense* strains have been identified as having an intact *erm*(41) gene instead of the expected deleted gene (Shallom et al., 2013). These previous studies show that the gene targets used lack the specificity to discriminate between all the subspecies, therefore limiting their utility for diagnostic applications. Another study used Subspecies-Specific Sequence Detection (SSSD) method based on DNA hybridization. However, the labelled probes (for *M. bolletii* and *M. massiliense*) identified the respective strain sequences but cross-reacted with a related *Mycobacterium* species: *M. chelonae* (Minias et al., 2020). Genotypic characterization of the 16S rRNA sequences have shown that there is 4bp difference between *M. abscessus* and *M. chelonae* but they both have identical hypervariable region A sequences (Brown-Elliott and Wallace, 2002; Leao et al., 2009). It is important to distinguish between *M. abscessus* and *M. chelonae* as they have both been associated with lung disease (Ko et al., 2013; Pease and Alvarez, 2021). However, treatment for *M. chelonae* is less complicated compared with *M. abscessus* as it is more susceptible to traditional antibiotics, i.e. clarithromycin (Ko et al., 2013).

In contrast, we have developed PCR assays that are able to distinguish between all three subspecies of MABC simultaneously. The Multiplex C assay which included all three subspecies and the other bacterial samples with all the primers in one tube would be the recommended assay to use. This is because the amplicon sizes are the easiest to distinguish from one another (with no cross reactivity) and the results are therefore easier to interpret. We found only two other reports of successful discrimination between the MABC subspecies using multiplex PCR DNA; the first following chromatography (Yoshida et al., 2021), and the second combining multiplex assay with molecular beacon probes to distinguish between the *M. abscessus* subspecies and determine antibiotic susceptibility (Marras et al., 2021). However, our assay has the advantage of reducing the processing steps to a single PCR reaction, which is cost-effective and reduces the turnaround time. In addition, we have included *M. chelonae* and other mycobacterial species of clinical importance, which cause or have been associated with chronic lung infections to evaluate the specificity and sensitivity of our assay and found the primers used in our assay produced no false reactions to these other mycobacteria. Another advantage of our PCR assays is the ease of adapting its application to other diagnostic tools such as Quantitative PCR (qPCR) due to the amplicon sizes. This allows for the addition of probes or intercalating dyes which will allow for the differentiation of the subspecies of MABC which is more cost effective than whole genome sequencing.

These results promise that, with further validation on a larger sample of clinical isolates, our test may provide a rapid, inexpensive, and accurate test for introduction into diagnostic laboratories worldwide. Rapid and unambiguous differentiation of MABC subspecies will provide improved disease surveillance and will inform antibiotic therapy.

## Data Availability Statement

The datasets presented in this study can be found in online repositories. The names of the repository/repositories and accession number(s) can be found in the article/**Supplementary Material**.

## Author Contributions

All the authors contributed to the conception and design of the study. SA, FM and MP provided the clinical strains used within the study. WA, AV, JJ, SA, MD, and FM contributed to the acquisition and analysis of data. WA, AV, MC and SHW contributed to the writing of the manuscript.

## Funding

This work was supported by the Engineering and Physical Sciences Research Council (EP/S51391X/1).

## Conflict of Interest

The authors declare that the research was conducted in the absence of any commercial or financial relationships that could be construed as a potential conflict of interest.

## Publisher’s Note

All claims expressed in this article are solely those of the authors and do not necessarily represent those of their affiliated organizations, or those of the publisher, the editors and the reviewers. Any product that may be evaluated in this article, or claim that may be made by its manufacturer, is not guaranteed or endorsed by the publisher.
