# Supplementary material for: The Use of Comparative Genomic Analysis for the Development of Subspecies-Specific PCR Assays for Mycobacterium abscessus
Source: Front Cell Infect Microbiol. 2022 Mar 28;12:816615. doi: 10.3389/fcimb.2022.816615 (PMC8995789; doi:10.3389/fcimb.2022.816615)
Supplement: Supplementary file 1 [file DataSheet_1.pdf]

Supplementary Figure S1

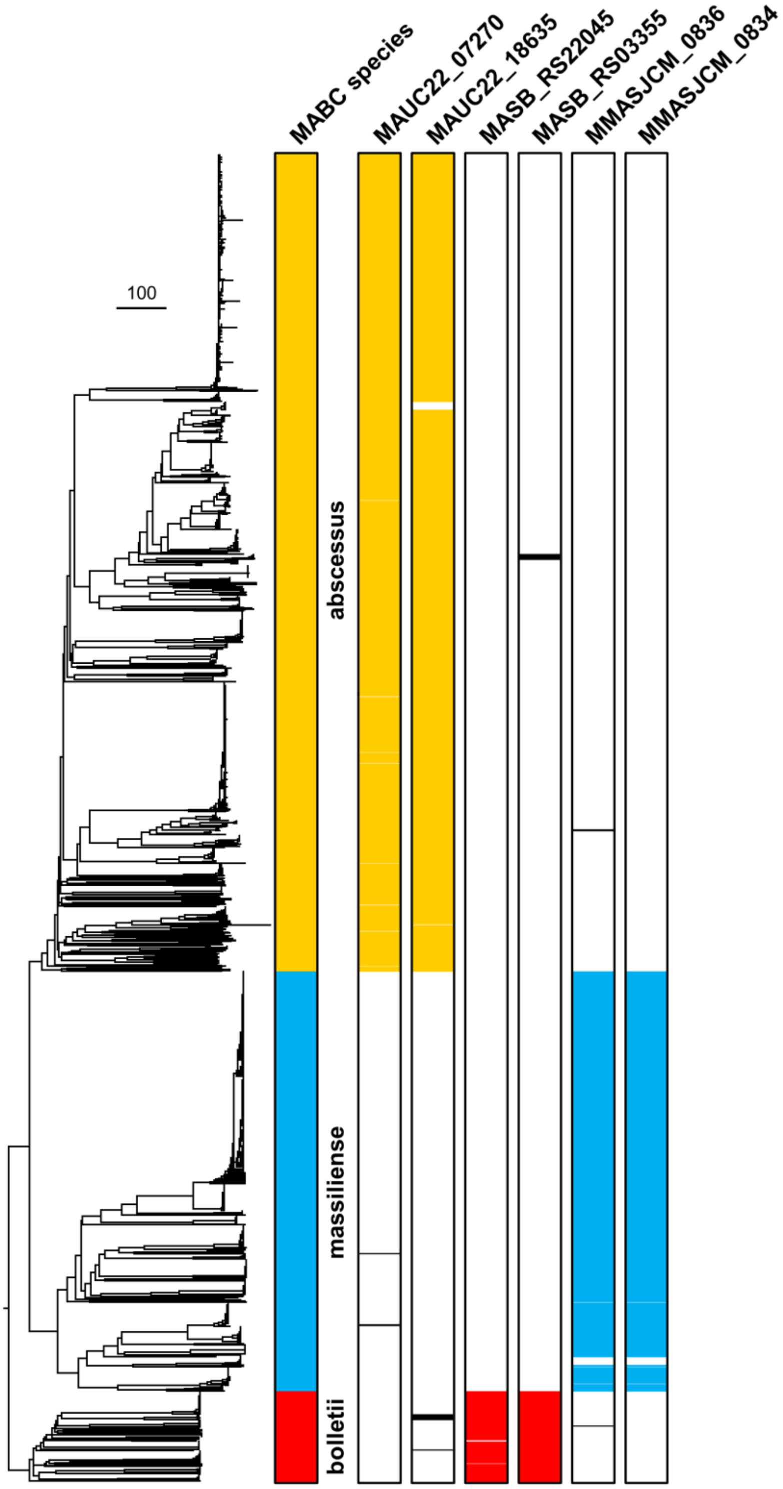

Figure S1. Phylogenetic tree of 1,663 genomes from the *Mycobacterium abscessus* complex (MABC), based on core genome MLST. The three clusters representing the subspecies *M. abscessus* subsp. *abscessus* (orange, abscessus), *M. abscessus* subsp. *bolletii* (red, bolletii) and *M. abscessus* subsp. *massiliense* (blue, massiliense) are indicated in the first bar. The other bars show the *in silico* PCR predictions with the MABC subspecies-specific PCRs (Tables 1 & 2). Orange, red and blue lines represent a match of the PCR with the relevant MABC subspecies, a black line represent a predicted PCR-positivity outside the associated MABC subspecies.

Supplementary Figure S2

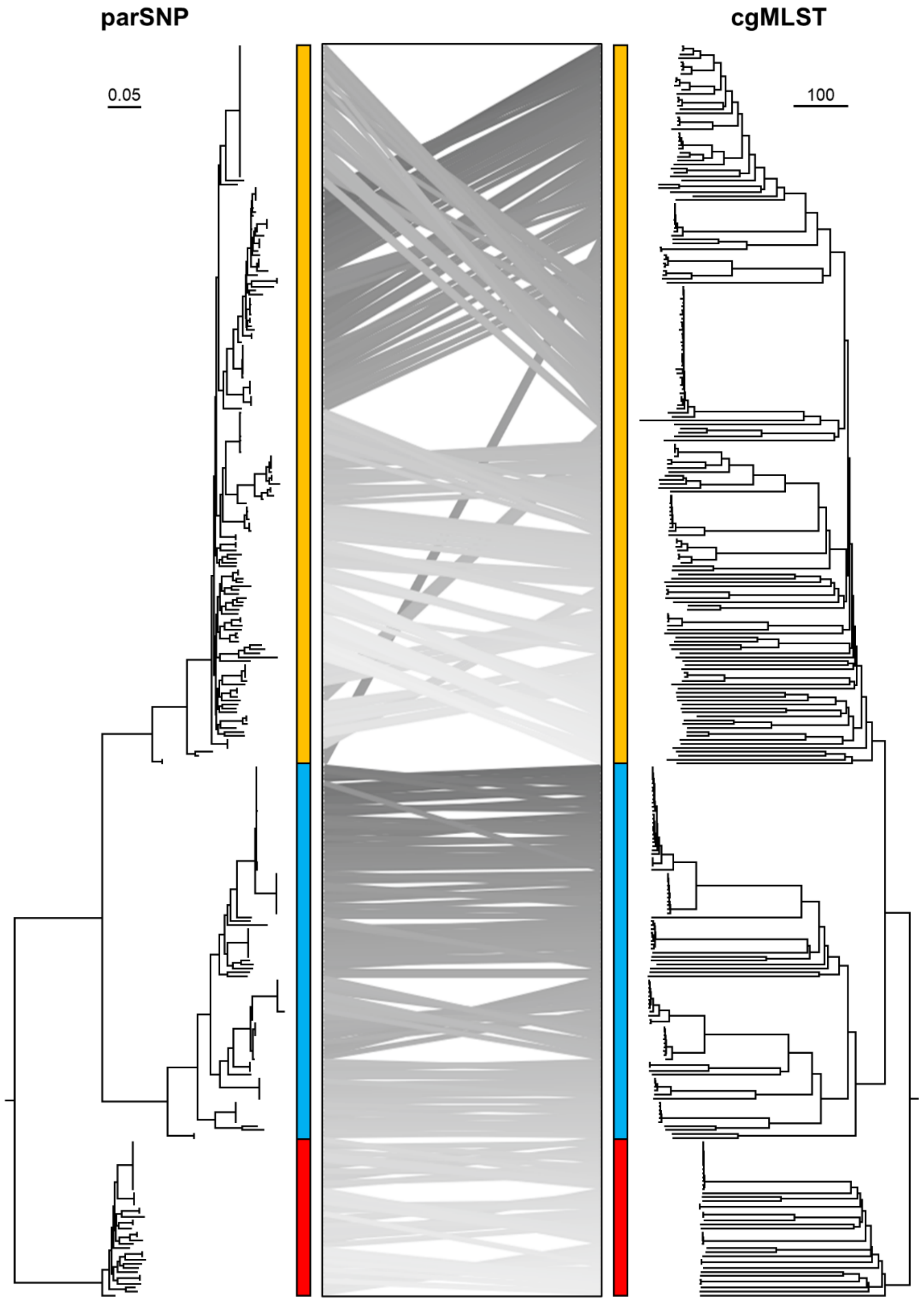

Figure S2. Tanglegram comparing phylogenetic trees derived from 318 genomes of the *Mycobacterium abscessus* complex (MABC), showing that clustering to identify the MABC subspecies is comparable between the two methods, but that within the MABC subspecies there are differences in clustering. The tree on the left is based on core genome single nucleotide polymorphisms, the tree on the right is based on core genome MLST. The three clusters representing the subspecies *M. abscessus* subsp. *abscessus* (orange), *M. abscessus* subsp. *bolletii* (red) and *M. abscessus* subsp. *massiliense* (blue) are indicated in the colour bars.

Table S1. Information of *Mycobacterium abscessus* Complex genomes used in this study, including their in silico PCR outcome with the 6 sets of PCR primers.

SNP[1]	cgMLST[1]	Code[2]	Organism/Name Genbank[3]	Strain	Assembly[4]	Source	MAUC22_07270	MAUC22_18635	MASB_RS22045	MASB_RS03355	MMASJCM_0836	MMASJCM_0834
1	245	Mbaa0288	M abscessus subsp abscessus	445	GCA_900138775	Respiratory system	Positive	Positive	negative	negative	negative	negative
2	262	Mbaa0783	M abscessus subsp abscessus	A189	GCA_002802045	lavage fluid	Positive	Positive	negative	negative	negative	negative
3	263	Mbaa0454	M abscessus subsp abscessus	703	GCA_900139255	Respiratory system	Positive	Positive	negative	negative	negative	negative
4	272	Mbaa0830	M abscessus subsp abscessus	A59	GCA_002802125	sputum	Positive	Positive	negative	negative	negative	negative
5	271	Mbaa0075	M abscessus subsp abscessus	86	GCA_002800775	lavage fluid	Positive	Positive	negative	negative	negative	negative
6	277	Mbaa1343	M abscessus	A213	GCA_003071925	sputum	Positive	Positive	negative	negative	negative	negative
7	283	Mbaa0325	M abscessus subsp abscessus	508	GCA_900137925	Respiratory system	Positive	Positive	negative	negative	negative	negative
8	294	Mbaa1661	M abscessus	UC95	GCA_001187745	human-associated habitat	Positive	Positive	negative	negative	negative	negative
9	246	Mbaa0366	M abscessus subsp abscessus	592	GCA_900139825	Respiratory system	Positive	Positive	negative	negative	negative	negative
10	207	Mbaa1337	M abscessus	6G-0212	GCA_000270645	sputum	Positive	Positive	negative	negative	negative	negative
11	184	Mbaa1311	M abscessus	1948	GCA_000523855	sputum	Positive	Positive	negative	negative	negative	negative
12	208	Mbaa1338	M abscessus	6G-0728-R	GCA_000270605	sputum	Positive	Positive	negative	negative	negative	negative
13	209	Mbaa1340	M abscessus	6G-1108	GCA_000271145	sputum	Positive	Positive	negative	negative	negative	negative
14	201	Mbaa0423	M abscessus subsp abscessus	665	GCA_900139195	Respiratory system	Positive	Positive	negative	negative	negative	negative
15	187	Mbaa1497	M abscessus	MAB_110811_2726	GCA_000523935	sputum	Positive	Positive	negative	negative	negative	negative
16	185	Mbaa1310	M abscessus	1518	GCA_000523895	sputum	Positive	Positive	negative	negative	negative	negative
17	60	Mbaa1359	M abscessus	FLAC013	GCA_001606295	Homo sapiens	Positive	Positive	negative	negative	negative	negative
18	55	Mbaa0409	M abscessus subsp abscessus	648	GCA_900140255	Respiratory system	Positive	Positive	negative	negative	negative	negative
19	54	Mbaa0394	M abscessus subsp abscessus	627	GCA_900139895	Respiratory system	Positive	Positive	negative	negative	negative	negative
20	221	Mbaa0087	M abscessus subsp abscessus	103	GCA_900135615	Respiratory system	Positive	Positive	negative	negative	negative	negative
21	35	Mbaa0191	M abscessus subsp abscessus	291	GCA_900131915	Respiratory system	Positive	Positive	negative	negative	negative	negative
22	189	Mbaa1477	M abscessus	GZ002	GCA_004028015	Homo sapiens	Positive	Positive	negative	negative	negative	negative
23	190	Mbaa1341	M abscessus	7C	GCA_003586385	Not available	Positive	Positive	negative	negative	negative	negative
24	192	Mbaa1499	M abscessus	NCTC13031	GCA_900453225	Not available	Positive	Positive	negative	negative	negative	negative
25	191	Mbaa0835	M abscessus subsp abscessus	ATCC-19977	GCA_000069185	Not available	Positive	Positive	negative	negative	negative	negative
26	64	Mbaa0422	M abscessus subsp abscessus	664	GCA_900140095	Respiratory system	Positive	Positive	negative	negative	negative	negative
27	107	Mbaa1478	M abscessus	GZ0-1	GCA_004027995	Homo sapiens	Positive	Positive	negative	negative	negative	negative
28	119	Mbaa1503	M abscessus	PAP004	GCA_001213565	sputum	Positive	Positive	negative	negative	negative	negative
29	70	Mbaa1363	M abscessus	FLAC029	GCA_001606315	Homo sapiens	Positive	Positive	negative	negative	negative	negative
30	69	Mbaa1362	M abscessus	FLAC028	GCA_001677155	Homo sapiens	Positive	Positive	negative	negative	negative	negative
31	80	Mbaa0793	M abscessus subsp abscessus	A243	GCA_002800545	sputum	Positive	Positive	negative	negative	negative	negative
32	159	Mbaa0068	M abscessus subsp abscessus	72	GCA_900135555	Respiratory system	Positive	Positive	negative	negative	negative	negative
33	218	Mbaa1317	M abscessus	3A-0810-R	GCA_000271105	bronchial alveolar lavage	Positive	Positive	negative	negative	negative	negative
34	219	Mbaa1314	M abscessus	3A-0122-R	GCA_000270845	sputum	Positive	Positive	negative	negative	negative	negative
35	296	Mbaa0091	M abscessus subsp abscessus	108	GCA_900136685	Respiratory system	Positive	Positive	negative	negative	negative	negative
36	310	Mbaa0017	M abscessus subsp abscessus	16	GCA_900139395	Respiratory system	Positive	Positive	negative	negative	negative	negative
37	383	Mbaa0200	M abscessus subsp abscessus	314	GCA_900132135	Respiratory system	Positive	Positive	negative	negative	negative	negative
38	393	Mbaa0190	M abscessus subsp abscessus	289	GCA_002801965	sputum	Positive	Positive	negative	negative	negative	negative
39	386	Mbaa0800	M abscessus subsp abscessus	A297	GCA_002800225	sputum	Positive	Positive	negative	negative	negative	negative
40	398	Mbaa0021	M abscessus subsp abscessus	21	GCA_002800385	lavage fluid	Positive	Positive	negative	negative	negative	negative
41	331	Mbaa0381	M abscessus subsp abscessus	610	GCA_900139075	Respiratory system	Positive	Positive	negative	negative	negative	negative
42	330	Mbaa0426	M abscessus subsp abscessus	669	GCA_900139165	Respiratory system	Positive	Positive	negative	negative	negative	negative
43	339	Mbaa0786	M abscessus subsp abscessus	A213	GCA_002800875	sputum	Positive	Positive	negative	negative	negative	negative
44	337	Mbaa0318	M abscessus subsp abscessus	501	GCA_900136485	Respiratory system	Positive	Positive	negative	negative	negative	negative
45	317	Mbaa0782	M abscessus subsp abscessus	A183	GCA_002800235	sputum	Positive	negative	negative	negative	negative	negative
46	320	Mbaa0775	M abscessus subsp abscessus	6382	GCA_002800275	lavage fluid	Positive	negative	negative	negative	negative	negative
47	318	Mbaa0810	M abscessus subsp abscessus	A330	GCA_002800205	lavage fluid	Positive	negative	negative	negative	negative	negative
48	326	Mbaa0171	M abscessus subsp abscessus	254	GCA_002801975	lavage fluid	Positive	Positive	negative	negative	negative	negative
49	328	Mbaa0831	M abscessus subsp abscessus	A69	GCA_002800255	sputum	Positive	Positive	negative	negative	negative	negative
50	355	Mbaa0269	M abscessus subsp abscessus	420	GCA_900134005	Respiratory system	Positive	Positive	negative	negative	negative	negative
51	360	Mbaa0232	M abscessus subsp abscessus	371	GCA_900133265	Respiratory system	Positive	Positive	negative	negative	negative	negative

52	350	Mbaa0235	M abscessus subsp abscessus	374	GCA_900133295	Respiratory system	Positive	Positive	negative	negative	negative	negative
53	411	Mbaa0044	M abscessus subsp abscessus	44	GCA_900135305	Respiratory system	Positive	Positive	negative	negative	negative	negative
54	348	Mbaa0304	M abscessus subsp abscessus	475	GCA_900138835	Respiratory system	Positive	Positive	negative	negative	negative	negative
55	402	Mbaa0143	M abscessus subsp abscessus	214	GCA_900130915	Respiratory system	Positive	Positive	negative	negative	negative	negative
56	399	Mbaa0148	M abscessus subsp abscessus	217	GCA_900130935	Respiratory system	Positive	Positive	negative	negative	negative	negative
57	401	Mbaa0836	M abscessus subsp abscessus	Bamboo	GCA_002072635	Sputum	Positive	Positive	negative	negative	negative	negative
58	404	Mbaa0019	M abscessus subsp abscessus	19	GCA_900135115	Respiratory system	Positive	Positive	negative	negative	negative	negative
59	514	Mbaa0435	M abscessus subsp abscessus	679	GCA_900139705	Respiratory system	Positive	Positive	negative	negative	negative	negative
60	508	Mbaa0261	M abscessus subsp abscessus	408	GCA_900138995	Respiratory system	Positive	Positive	Positive	negative	negative	negative
61	502	Mbaa0285	M abscessus subsp abscessus	439	GCA_900134725	Respiratory system	Positive	Positive	Positive	negative	negative	negative
62	509	Mbaa0756	M abscessus subsp abscessus	3476	GCA_002800315	sputum	Positive	Positive	negative	negative	negative	negative
63	499	Mbaa0163	M abscessus subsp abscessus	242	GCA_900131275	Respiratory system	Positive	Positive	negative	negative	negative	negative
64	540	Mbaa1482	M abscessus	M152	GCA_000280615	sputum	Positive	Positive	negative	negative	negative	negative
65	468	Mbaa0129	M abscessus subsp abscessus	180	GCA_900130595	Respiratory system	Positive	Positive	negative	negative	negative	negative
66	455	Mbaa0287	M abscessus subsp abscessus	444	GCA_900134805	Respiratory system	Positive	Positive	negative	negative	negative	negative
67	452	Mbaa0186	M abscessus subsp abscessus	281	GCA_900131675	Respiratory system	Positive	Positive	negative	negative	negative	negative
68	461	Mbaa0215	M abscessus subsp abscessus	340	GCA_900132855	Respiratory system	Positive	Positive	negative	negative	negative	negative
69	467	Mbaa0174	M abscessus subsp abscessus	258	GCA_900131415	Respiratory system	Positive	Positive	negative	negative	negative	negative
70	470	Mbaa0003	M abscessus subsp abscessus	2	GCA_900138755	Respiratory system	Positive	Positive	negative	negative	negative	negative
71	438	Mbaa0302	M abscessus subsp abscessus	472	GCA_900138785	Respiratory system	Positive	Positive	negative	negative	negative	negative
72	449	Mbaa0109	M abscessus subsp abscessus	140	GCA_900139615	Respiratory system	Positive	Positive	negative	negative	negative	negative
73	424	Mbaa0351	M abscessus subsp abscessus	575	GCA_900139045	Respiratory system	Positive	Positive	negative	negative	negative	negative
74	496	Mbaa0777	M abscessus subsp abscessus	06_0524	GCA_001942565	seakrait	Positive	Positive	negative	negative	negative	negative
75	495	Mbaa1489	M abscessus	M94	GCA_000257245	sputum	Positive	Positive	negative	negative	negative	negative
76	498	Mbaa0139	M abscessus subsp abscessus	209	GCA_900140335	Respiratory system	Positive	Positive	negative	negative	negative	negative
77	567	Mbaa0124	M abscessus subsp abscessus	172	GCA_900130495	Respiratory system	Positive	Positive	negative	negative	negative	negative
78	560	Mbaa0127	M abscessus subsp abscessus	176	GCA_900130555	Respiratory system	Positive	Positive	negative	negative	negative	negative
79	563	Mbaa0176	M abscessus subsp abscessus	264	GCA_900140435	Respiratory system	Positive	Positive	negative	negative	negative	negative
80	562	Mbaa0159	M abscessus subsp abscessus	233	GCA_900131195	Respiratory system	Positive	Positive	negative	negative	negative	negative
81	566	Mbaa0173	M abscessus subsp abscessus	255	GCA_900131395	Respiratory system	Positive	Positive	negative	negative	negative	negative
82	565	Mbaa0161	M abscessus subsp abscessus	235	GCA_900131185	Respiratory system	Positive	Positive	negative	negative	negative	negative
83	561	Mbaa0130	M abscessus subsp abscessus	181	GCA_900130605	Respiratory system	Positive	Positive	negative	negative	negative	negative
84	564	Mbaa0165	M abscessus subsp abscessus	245	GCA_900131285	Respiratory system	Positive	Positive	negative	negative	negative	negative
85	568	Mbaa1305	M abscessus	21	GCA_000523835	sputum	Positive	Positive	negative	negative	negative	negative
86	546	Mbaa0799	M abscessus subsp abscessus	A274	GCA_002801095	sputum	Positive	Positive	negative	negative	negative	negative
87	545	Mbaa0114	M abscessus subsp abscessus	146	GCA_002802645	sputum	Positive	Positive	negative	negative	negative	negative
88	548	Mbaa0277	M abscessus subsp abscessus	429	GCA_900134985	Respiratory system	Positive	Positive	negative	negative	negative	negative
89	547	Mbaa0278	M abscessus subsp abscessus	430	GCA_900135005	Respiratory system	Positive	Positive	negative	negative	negative	negative
90	553	Mbaa0144	M abscessus subsp abscessus	215	GCA_002801165	sputum	Positive	Positive	negative	negative	negative	negative
91	552	Mbaa0787	M abscessus subsp abscessus	A215	GCA_002801115	sputum	Positive	Positive	negative	negative	negative	negative
92	551	Mbaa0811	M abscessus subsp abscessus	A337	GCA_002802525	sputum	Positive	Positive	negative	negative	negative	negative
93	571	Mbaa0099	M abscessus subsp abscessus	122	GCA_900136815	Respiratory system	Positive	Positive	negative	negative	negative	negative
94	737	Mbaa0085	M abscessus subsp abscessus	101	GCA_900135575	Respiratory system	Positive	Positive	negative	negative	negative	negative
95	824	Mbaa0041	M abscessus subsp abscessus	40	GCA_900135255	Respiratory system	Positive	Positive	negative	negative	negative	negative
96	761	Mbaa0323	M abscessus subsp abscessus	506	GCA_900136545	Respiratory system	Positive	Positive	negative	negative	negative	negative
97	760	Mbaa0839	M abscessus subsp abscessus	Patient_2_S1	GCA_900683455	Not available	Positive	Positive	negative	negative	negative	negative
98	741	Mbaa0175	M abscessus subsp abscessus	263	GCA_900131765	Respiratory system	Positive	Positive	negative	negative	negative	negative
99	740	Mbaa0837	M abscessus subsp abscessus	MA667	GCA_004025145	sputum	Positive	Positive	negative	negative	negative	negative
100	681	Mbaa0316	M abscessus subsp abscessus	498	GCA_900136565	Respiratory system	Positive	Positive	negative	negative	negative	negative
101	764	Mbaa0838	M abscessus subsp abscessus	Patient_1_S1	GCA_900683445	Not available	negative	Positive	negative	negative	negative	negative
102	751	Mbaa0364	M abscessus subsp abscessus	590	GCA_900139965	Respiratory system	Positive	Positive	negative	negative	negative	negative
103	755	Mbaa1361	M abscessus	FLAC015	GCA_002142845	Homo sapiens	Positive	Positive	negative	negative	negative	negative
104	818	Mbaa0346	M abscessus subsp abscessus	550	GCA_900138005	Respiratory system	Positive	Positive	negative	negative	negative	negative

105	827	Mbaa0297	M abscessus subsp abscessus	462	GCA_900138745	Respiratory system	Positive	Positive	negative	negative	negative	negative
106	825	Mbaa0268	M abscessus subsp abscessus	419	GCA_900139015	Respiratory system	Positive	Positive	negative	negative	negative	negative
107	831	Mbaa0217	M abscessus subsp abscessus	343	GCA_900132775	Respiratory system	Positive	Positive	negative	negative	negative	negative
108	826	Mbaa0290	M abscessus subsp abscessus	447	GCA_900134825	Respiratory system	Positive	Positive	negative	negative	negative	negative
109	844	Mbaa0070	M abscessus subsp abscessus	74	GCA_900133505	Respiratory system	Positive	Positive	negative	negative	negative	negative
110	842	Mbaa0816	M abscessus subsp abscessus	A377	GCA_002800415	lavage fluid	Positive	Positive	negative	negative	negative	negative
111	840	Mbaa1424	M abscessus	G164	GCA_003582435	sputum	Positive	Positive	negative	negative	negative	negative
112	843	Mbaa0779	M abscessus subsp abscessus	A175	GCA_002800605	sputum	Positive	Positive	negative	negative	negative	negative
113	868	Mbaa0078	M abscessus subsp abscessus	89	GCA_900139515	Respiratory system	Positive	Positive	negative	negative	negative	negative
114	859	Mbaa0308	M abscessus subsp abscessus	484	GCA_900138895	Respiratory system	Positive	Positive	negative	negative	negative	negative
115	865	Mbaa0784	M abscessus subsp abscessus	A197	GCA_002800585	lavage fluid	Positive	Positive	negative	negative	negative	negative
116	862	Mbaa0107	M abscessus subsp abscessus	131	GCA_900139535	Respiratory system	Positive	Positive	negative	negative	negative	negative
117	869	Mbaa1660	M abscessus	UC22	GCA_001050395	pulmonary disease	Positive	Positive	negative	negative	negative	negative
118	872	Mbaa1309	M abscessus	206	GCA_003072365	sputum	Positive	Positive	negative	negative	negative	negative
119	870	Mbaa0815	M abscessus subsp abscessus	A373	GCA_002802485	lavage fluid	Positive	Positive	negative	negative	negative	negative
120	881	Mbaa0105	M abscessus subsp abscessus	129	GCA_002801245	sputum	Positive	Positive	negative	negative	negative	negative
121	884	Mbaa1365	M abscessus	FLAC031	GCA_001606335	Homo sapiens	Positive	Positive	negative	negative	negative	negative
122	887	Mbaa0334	M abscessus subsp abscessus	522	GCA_900140165	Respiratory system	Positive	Positive	negative	negative	negative	negative
123	886	Mbaa0328	M abscessus subsp abscessus	513	GCA_900140145	Respiratory system	Positive	Positive	negative	negative	negative	negative
124	888	Mbaa0301	M abscessus subsp abscessus	470	GCA_900138765	Respiratory system	Positive	Positive	negative	negative	negative	negative
125	899	Mbaa0812	M abscessus subsp abscessus	A35	GCA_002802435	sputum	Positive	Positive	negative	negative	negative	negative
126	905	Mbaa0189	M abscessus subsp abscessus	288	GCA_002802615	sputum	Positive	Positive	negative	negative	negative	negative
127	660	Mbaa1321	M abscessus	4S-0116-R	GCA_000270685	sputum	Positive	Positive	negative	negative	negative	negative
128	658	Mbaa1326	M abscessus	4S-0726-RB	GCA_000271265	sputum	Positive	Positive	negative	negative	negative	negative
129	653	Mbaa0470	M abscessus subsp abscessus	722	GCA_900139295	Respiratory system	Positive	Positive	negative	negative	negative	negative
130	907	Mbaa0182	M abscessus subsp abscessus	277	GCA_900131645	Respiratory system	Positive	Positive	negative	negative	negative	negative
131	906	Mbaa0395	M abscessus subsp abscessus	629	GCA_900140815	Respiratory system	Positive	Positive	negative	negative	negative	negative
132	908	Mbaa0121	M abscessus subsp abscessus	165	GCA_900130265	Respiratory system	Positive	Positive	negative	negative	negative	negative
133	909	Mbaa0794	M abscessus subsp abscessus	A244	GCA_002801575	sputum	Positive	Positive	negative	negative	negative	negative
134	1014	Mbaa0023	M abscessus subsp abscessus	22	GCA_002802765	sputum	Positive	Positive	negative	negative	negative	negative
135	1011	Mbaa0797	M abscessus subsp abscessus	A255	GCA_002802695	lavage fluid	Positive	Positive	negative	negative	negative	negative
136	1006	Mbaa0431	M abscessus subsp abscessus	674	GCA_900139155	Respiratory system	Positive	Positive	negative	negative	negative	negative
137	1008	Mbaa0128	M abscessus subsp abscessus	178	GCA_002801685	lavage fluid	Positive	Positive	negative	negative	negative	negative
138	996	Mbaa1421	M abscessus	G160	GCA_003071705	Bronchoalveolar lavage	Positive	Positive	negative	negative	negative	negative
139	997	Mbaa0814	M abscessus subsp abscessus	A353	GCA_002802655	sputum	Positive	Positive	negative	negative	negative	negative
140	1000	Mbaa1447	M abscessus	G201	GCA_003582005	sputum	Positive	Positive	negative	negative	negative	negative
141	987	Mbaa0164	M abscessus subsp abscessus	244	GCA_002800855	sputum	Positive	Positive	negative	negative	negative	negative
142	985	Mbaa0823	M abscessus subsp abscessus	A477	GCA_002802295	sputum	Positive	Positive	negative	negative	negative	negative
143	983	Mbaa0805	M abscessus subsp abscessus	A315	GCA_002801425	lavage fluid	Positive	Positive	negative	negative	negative	negative
144	975	Mbaa0789	M abscessus subsp abscessus	A218	GCA_002801435	sputum	Positive	Positive	negative	negative	negative	negative
145	992	Mbaa0432	M abscessus subsp abscessus	675	GCA_900139205	Respiratory system	Positive	Positive	negative	negative	negative	negative
146	977	Mbaa0840	M abscessus subsp abscessus	Z58	GCA_000611915	pneumonia patient	Positive	Positive	negative	negative	negative	negative
147	978	Mbaa1663	M abscessus	Z61	GCA_000611935	pneumonia patient	Positive	Positive	negative	negative	negative	negative
148	980	Mbaa0796	M abscessus subsp abscessus	A25	GCA_002801015	sputum	Positive	Positive	negative	negative	negative	negative
149	981	Mbaa0791	M abscessus subsp abscessus	A232	GCA_002801105	sputum	Positive	Positive	negative	negative	negative	negative
150	960	Mbaa0746	M abscessus subsp abscessus	1866	GCA_002802845	lavage fluid	Positive	Positive	negative	negative	negative	negative
151	958	Mbaa0755	M abscessus subsp abscessus	3384	GCA_002801345	lavage fluid	Positive	Positive	negative	negative	negative	negative
152	969	Mbaa0807	M abscessus subsp abscessus	A321	GCA_002802335	lavage fluid	Positive	Positive	negative	negative	negative	negative
153	1021	Mbaa0771	M abscessus subsp abscessus	5725	GCA_002802685	lavage fluid	Positive	Positive	negative	negative	negative	negative
154	1024	Mbaa0745	M abscessus subsp abscessus	1791	GCA_002802585	sputum	Positive	Positive	negative	negative	negative	negative
155	1020	Mbaa0809	M abscessus subsp abscessus	A329	GCA_002802415	sputum	Positive	Positive	negative	negative	negative	negative
156	1022	Mbaa0817	M abscessus subsp abscessus	A378	GCA_002801585	sputum	Positive	Positive	negative	negative	negative	negative
157	1018	Mbaa0750	M abscessus subsp abscessus	2767	GCA_002802205	sputum	negative	Positive	negative	negative	negative	negative

158	598	Mbaa0073	M abscessus subsp abscessus	79	GCA_900134165	Respiratory system	Positive	Positive	negative	negative	negative	negative
159	583	Mbaa0781	M abscessus subsp abscessus	A182	GCA_002800805	lavage fluid	Positive	Positive	negative	negative	negative	negative
160	607	Mbaa0029	M abscessus subsp abscessus	27	GCA_002800965	lavage fluid	Positive	Positive	negative	negative	negative	negative
161	573	Mbaa0311	M abscessus subsp abscessus	488	GCA_900138945	Respiratory system	Positive	Positive	negative	negative	negative	negative
162	612	Mbaa0020	M abscessus subsp abscessus	20	GCA_900135155	Respiratory system	Positive	Positive	negative	negative	negative	negative
163	1016	Mbaa1490	M abscessus	MAB_030201_1061	GCA_000523775	bronchoalveolar lavage	Positive	Positive	negative	negative	negative	negative
164	640	Mbaa0392	M abscessus subsp abscessus	625	GCA_900140795	Respiratory system	Positive	Positive	negative	negative	negative	negative
165	637	Mbaa0429	M abscessus subsp abscessus	672	GCA_900141025	Respiratory system	Positive	Positive	negative	negative	negative	negative
166	632	Mbaa0380	M abscessus subsp abscessus	608	GCA_900139815	Respiratory system	Positive	Positive	negative	negative	negative	negative
167	642	Mbaa0828	M abscessus subsp abscessus	A54	GCA_002801255	sputum	Positive	Positive	negative	negative	negative	negative
168	941	Mbaa1488	M abscessus	M93	GCA_000257225	sputum	negative	Positive	negative	negative	negative	negative
169	943	Mbaa0185	M abscessus subsp abscessus	280	GCA_900131665	Respiratory system	Positive	Positive	negative	negative	negative	negative
170	942	Mbaa0207	M abscessus subsp abscessus	329	GCA_900140415	Respiratory system	Positive	Positive	negative	negative	negative	negative
171	931	Mbaa0352	M abscessus subsp abscessus	576	GCA_900141285	Respiratory system	Positive	Positive	negative	negative	negative	negative
172	929	Mbaa1370	M abscessus	FLAC049	GCA_001610655	Homo sapiens	Positive	Positive	negative	negative	negative	negative
173	932	Mbaa0455	M abscessus subsp abscessus	706	GCA_900139675	Respiratory system	Positive	Positive	negative	negative	negative	negative
174	928	Mbaa0224	M abscessus subsp abscessus	355	GCA_900132665	Respiratory system	Positive	Positive	negative	negative	negative	negative
175	933	Mbaa0758	M abscessus subsp abscessus	3617	GCA_002801355	lavage fluid	Positive	Positive	negative	negative	negative	negative
176	661	Mbaa0137	M abscessus subsp abscessus	205	GCA_002801325	sputum	Positive	Positive	negative	negative	negative	negative
177	913	Mbaa0339	M abscessus subsp abscessus	533	GCA_900140245	Respiratory system	Positive	Positive	negative	negative	negative	negative
178	914	Mbaa0338	M abscessus subsp abscessus	531	GCA_900139925	Respiratory system	Positive	Positive	negative	negative	negative	negative
179	911	Mbaa0340	M abscessus subsp abscessus	534	GCA_900139945	Respiratory system	Positive	Positive	negative	negative	negative	negative
180	534	Mbaa0153	M abscessus subsp abscessus	223	GCA_900131075	Respiratory system	Positive	Positive	negative	negative	negative	negative
181	535	Mbaa0239	M abscessus subsp abscessus	379	GCA_900133325	Respiratory system	Positive	Positive	negative	negative	negative	negative
182	521	Mbaa0233	M abscessus subsp abscessus	372	GCA_900133275	Respiratory system	Positive	Positive	negative	negative	negative	negative
183	526	Mbaa0135	M abscessus subsp abscessus	196	GCA_900140325	Respiratory system	Positive	Positive	negative	negative	negative	negative
184	1178	Mbam1044	M abscessus subsp massiliense	307	GCA_900132075	Respiratory system	negative	negative	negative	negative	Positive	Positive
185	1214	Mbam1052	M abscessus subsp massiliense	346	GCA_900132815	Respiratory system	negative	negative	negative	negative	Positive	Positive
186	1193	Mbam1495	M abscessus	MAB_091912_2455	GCA_000523915	Homo sapiens	negative	negative	negative	negative	Positive	Positive
187	1187	Mbam0943	M abscessus subsp bolletii	2B-0107	GCA_000270945	sputum	negative	negative	negative	negative	Positive	Positive
188	1288	Mbam1492	M abscessus	MAB_082312_2258	GCA_000500165	sputum	negative	negative	negative	negative	Positive	Positive
189	1188	Mbam0944	M abscessus subsp bolletii	2B-0307	GCA_000270765	sputum	negative	negative	negative	negative	Positive	Positive
190	1190	Mbam0947	M abscessus subsp bolletii	2B-0912-S	GCA_000270785	sputum	negative	negative	negative	negative	Positive	Positive
191	1189	Mbam0945	M abscessus subsp bolletii	2B-0626	GCA_000270905	bronchial alveolar lavage	negative	negative	negative	negative	Positive	Positive
192	1195	Mbam1226	M abscessus subsp massiliense	3166	GCA_002800055	sputum	negative	negative	negative	negative	Positive	Positive
193	1196	Mbam1253	M abscessus subsp massiliense	A126	GCA_002801765	sputum	negative	negative	negative	negative	Positive	Positive
194	1218	Mbam1064	M abscessus subsp massiliense	451	GCA_900134865	Respiratory system	negative	negative	negative	negative	Positive	Positive
195	1198	Mbam1385	M abscessus	G110	GCA_003582945	sputum	negative	negative	negative	negative	Positive	Positive
196	1166	Mbam1274	M abscessus subsp massiliense	A63	GCA_002799645	sputum	negative	negative	negative	negative	Positive	Positive
197	1094	Mbam0998	M abscessus subsp massiliense	192	GCA_900130745	Respiratory system	negative	negative	negative	negative	Positive	Positive
198	1100	Mbam1356	M abscessus	FLAC007	GCA_001606255	Homo sapiens	negative	negative	negative	negative	Positive	Positive
199	1095	Mbam1353	M abscessus	FLAC004	GCA_001606215	Homo sapiens	negative	negative	negative	negative	Positive	Positive
200	1083	Mbam1050	M abscessus subsp massiliense	326	GCA_900132385	Respiratory system	negative	negative	negative	negative	Positive	Positive
201	1090	Mbam1367	M abscessus	FLAC045	GCA_001610615	Homo sapiens	negative	negative	negative	negative	Positive	Positive
202	1107	Mbam0996	M abscessus subsp massiliense	186	GCA_900130625	Respiratory system	negative	negative	negative	negative	Positive	Positive
203	1025	Mbam1223	M abscessus subsp massiliense	1178	GCA_900138495	Respiratory system	negative	negative	negative	negative	Positive	Positive
204	1102	Mbam0966	M abscessus subsp massiliense	111	GCA_002800155	lavage fluid	negative	negative	negative	negative	Positive	Positive
205	1127	Mbam0953	M abscessus subsp bolletii	M18	GCA_000261085	lymph node	negative	negative	negative	negative	Positive	Positive
206	1128	Mbam1046	M abscessus subsp massiliense	309	GCA_900132105	Respiratory system	negative	negative	negative	negative	Positive	Positive
207	1274	Mbam0960	M abscessus subsp massiliense	60	GCA_002801905	sputum	negative	negative	negative	negative	Positive	Positive
208	1270	Mbam0951	M abscessus subsp bolletii	CRM-0020	GCA_000418575	skin	negative	negative	negative	negative	Positive	Positive
209	1271	Mbam0952	M abscessus subsp bolletii	INCQS-00594	GCA_000363915	surgical-site abscess	negative	negative	negative	negative	Positive	Positive
210	1273	Mbam1301	M abscessus subsp massiliense	GO-06	GCA_000277775	Not available	negative	negative	negative	negative	Positive	Positive

211	1301	Mbam0940	M abscessus subsp bolletii	1S-152-0914	GCA_000271285	bronchial alveolar lavage	negative	negative	negative	negative	Positive	Positive
212	1291	Mbam0941	M abscessus subsp bolletii	1S-153-0915	GCA_000270885	sputum	negative	negative	negative	negative	Positive	Positive
213	1292	Mbam1060	M abscessus subsp massiliense	416	GCA_900134025	Respiratory system	negative	negative	negative	negative	Positive	Positive
214	1313	Mbam1265	M abscessus subsp massiliense	A295	GCA_002799775	sputum	negative	negative	negative	negative	Positive	Positive
215	1310	Mbam1225	M abscessus subsp massiliense	3106	GCA_002801875	sputum	negative	negative	negative	negative	Positive	Positive
216	1297	Mbam1229	M abscessus subsp massiliense	4070	GCA_002800025	sputum	negative	negative	negative	negative	Positive	Positive
217	1305	Mbam1081	M abscessus subsp massiliense	535	GCA_900139755	Respiratory system	negative	negative	negative	negative	Positive	Positive
218	1303	Mbam1467	M abscessus	G85	GCA_003071885	Bronchoalveolar lavage	negative	negative	negative	negative	Positive	Positive
219	1323	Mbam0956	M abscessus subsp massiliense	26	GCA_900135175	Respiratory system	negative	negative	negative	negative	Positive	Positive
220	1304	Mbam1264	M abscessus subsp massiliense	A289	GCA_002801735	sputum	negative	negative	negative	negative	Positive	Positive
221	1309	Mbam1042	M abscessus subsp massiliense	303	GCA_900132035	Respiratory system	negative	negative	negative	negative	Positive	Positive
222	1385	Mbam1485	M abscessus	M159	GCA_000261105	sputum	negative	negative	negative	negative	Positive	Positive
223	1342	Mbam0938	M abscessus subsp bolletii	50594	GCA_000445035	Not available	negative	negative	negative	negative	Positive	Positive
224	1433	Mbam1479	M abscessus	M115	GCA_000260595	sputum	negative	negative	negative	negative	Positive	Positive
225	1360	Mbam1071	M abscessus subsp massiliense	483	GCA_900138915	Respiratory system	negative	negative	negative	negative	Positive	Positive
226	1368	Mbam0969	M abscessus subsp massiliense	115	GCA_900136755	Respiratory system	negative	negative	negative	negative	Positive	Positive
227	1369	Mbam1483	M abscessus	M154	GCA_000260575	sputum	negative	negative	negative	negative	Positive	Positive
228	1355	Mbam1272	M abscessus subsp massiliense	A47	GCA_002799795	sputum	negative	negative	negative	negative	Positive	Positive
229	1343	Mbam0974	M abscessus subsp massiliense	132	GCA_900139585	Respiratory system	negative	negative	negative	negative	Positive	Positive
230	1357	Mbam1231	M abscessus subsp massiliense	5620	GCA_002799995	lavage fluid	negative	negative	negative	negative	Positive	Positive
231	1358	Mbam1484	M abscessus	M156	GCA_000280635	sputum	negative	negative	negative	negative	Positive	Positive
232	1359	Mbam1486	M abscessus	M172	GCA_000261125	sputum	negative	negative	negative	negative	Positive	Positive
233	1430	Mbam1260	M abscessus subsp massiliense	A247	GCA_002799855	sputum	negative	negative	negative	negative	Positive	Positive
234	1429	Mbam1087	M abscessus subsp massiliense	616	GCA_900140735	Respiratory system	negative	negative	negative	negative	Positive	Positive
235	1432	Mbam1076	M abscessus subsp massiliense	499	GCA_900136495	Respiratory system	negative	negative	negative	negative	Positive	Positive
236	1438	Mbam1065	M abscessus subsp massiliense	452	GCA_900134835	Respiratory system	negative	negative	negative	negative	Positive	Positive
237	1439	Mbam1303	M abscessus subsp massiliense	Myco1poa	GCA_003861345	sputum	negative	negative	negative	negative	negative	negative
238	1443	Mbam1298	M abscessus subsp massiliense	FLAC047	GCA_002140035	Homo sapiens	negative	negative	negative	negative	Positive	Positive
239	1442	Mbam1299	M abscessus subsp massiliense	GD01A	GCA_004209815	Homo sapiens	negative	negative	negative	negative	Positive	Positive
240	1441	Mbam1300	M abscessus subsp massiliense	GD01B	GCA_004209835	Homo sapiens	negative	negative	negative	negative	Positive	Positive
241	1440	Mbam1069	M abscessus subsp massiliense	478	GCA_900138855	Respiratory system	negative	negative	negative	negative	Positive	Positive
242	1460	Mbam1364	M abscessus	FLAC030	GCA_001677175	Homo sapiens	negative	negative	negative	negative	Positive	Positive
243	1459	Mbam1098	M abscessus subsp massiliense	677	GCA_900141085	Respiratory system	negative	negative	negative	negative	Positive	Positive
244	1447	Mbam1101	M abscessus subsp massiliense	688	GCA_900139795	Respiratory system	negative	negative	negative	negative	Positive	Positive
245	1453	Mbam1369	M abscessus	FLAC048	GCA_001610635	Homo sapiens	negative	negative	negative	negative	Positive	Positive
246	1467	Mbam1099	M abscessus subsp massiliense	681	GCA_900141135	Respiratory system	Positive	negative	negative	negative	Positive	Positive
247	1490	Mbam1302	M abscessus subsp massiliense	JCM-15300	GCA_000497265	Not available	negative	negative	negative	negative	Positive	Positive
248	1489	Mbam1297	M abscessus subsp massiliense	CIP-108297	GCA_001792625	sputum, bronchial lavage	negative	negative	negative	negative	Positive	Positive
249	1479	Mbam1047	M abscessus subsp massiliense	312	GCA_900132125	Respiratory system	negative	negative	negative	negative	Positive	Positive
250	1481	Mbam1041	M abscessus subsp massiliense	301	GCA_900132025	Respiratory system	negative	negative	negative	negative	Positive	Positive
251	1480	Mbam1051	M abscessus subsp massiliense	335	GCA_900132475	Respiratory system	negative	negative	negative	negative	Positive	Positive
252	1482	Mbam1000	M abscessus subsp massiliense	197	GCA_900130765	Respiratory system	negative	negative	negative	negative	Positive	Positive
253	1474	Mbam0961	M abscessus subsp massiliense	60	GCA_900135445	Respiratory system	negative	negative	negative	negative	Positive	Positive
254	1484	Mbam0957	M abscessus subsp massiliense	58	GCA_900135425	Respiratory system	negative	negative	negative	negative	Positive	Positive
255	1470	Mbam0959	M abscessus subsp massiliense	59	GCA_900133445	Respiratory system	negative	negative	negative	negative	Positive	Positive
256	1471	Mbam1019	M abscessus subsp massiliense	247	GCA_900131315	Respiratory system	negative	negative	negative	negative	Positive	Positive
257	1472	Mbam1067	M abscessus subsp massiliense	474	GCA_900138795	Respiratory system	negative	negative	negative	negative	Positive	Positive
258	1483	Mbam1073	M abscessus subsp massiliense	492	GCA_900136455	Respiratory system	negative	negative	negative	negative	Positive	Positive
259	1504	Mbam0981	M abscessus subsp massiliense	152	GCA_900139625	Respiratory system	negative	negative	negative	negative	Positive	Positive
260	1501	Mbam1014	M abscessus subsp massiliense	237	GCA_900131225	Respiratory system	negative	negative	negative	negative	Positive	Positive
261	1500	Mbam0982	M abscessus subsp massiliense	153	GCA_900130355	Respiratory system	negative	negative	negative	negative	Positive	Positive
262	1505	Mbam1376	M abscessus	FLAC055	GCA_001677215	Homo sapiens	negative	negative	negative	negative	Positive	Positive
263	1512	Mbam1333	M abscessus	5S-1212	GCA_000271025	sputum	negative	negative	negative	negative	negative	negative

264	1520	Mbam0937	M abscessus subsp bolletii	1513	GCA_000523875	sputum	negative	negative	negative	negative	negative	negative
265	1513	Mbam1328	M abscessus	5S-0421	GCA_000271225	sputum	negative	negative	negative	negative	negative	negative
266	1514	Mbam1327	M abscessus	5S-0304	GCA_000271205	sputum	negative	negative	negative	negative	negative	negative
267	1516	Mbam0971	M abscessus subsp massiliense	124	GCA_900136825	Respiratory system	negative	negative	negative	negative	negative	negative
268	1517	Mbam0970	M abscessus subsp massiliense	123	GCA_900136785	Respiratory system	negative	negative	negative	negative	negative	negative
269	1526	Mbam1230	M abscessus subsp massiliense	5601	GCA_002799985	sputum	negative	negative	negative	negative	Positive	Positive
270	1529	Mbam1397	M abscessus	G124	GCA_003582785	sputum	negative	negative	negative	negative	Positive	Positive
271	1527	Mbam1259	M abscessus subsp massiliense	A228	GCA_002799875	sputum	negative	negative	negative	negative	Positive	Positive
272	1537	Mbam1062	M abscessus subsp massiliense	441	GCA_900138735	Respiratory system	negative	negative	negative	negative	Positive	Positive
273	1521	Mbam1074	M abscessus subsp massiliense	495	GCA_900136465	Respiratory system	negative	negative	negative	negative	Positive	Positive
274	1531	Mbam1354	M abscessus	FLAC005	GCA_001606235	Homo sapiens	negative	negative	negative	negative	Positive	Positive
275	1550	Mbam1355	M abscessus	FLAC006	GCA_001677095	Homo sapiens	negative	negative	negative	negative	Positive	Positive
276	1549	Mbam1480	M abscessus	M139	GCA_000280595	sputum	negative	negative	negative	negative	Positive	Positive
277	1435	Mbam0955	M abscessus subsp massiliense	12	GCA_900139355	Respiratory system	negative	negative	negative	negative	Positive	Positive
278	1434	Mbam0954	M abscessus subsp massiliense	6	GCA_900139445	Respiratory system	negative	negative	negative	negative	Positive	Positive
279	1571	Mbab0865	M abscessus subsp bolletii	286	GCA_900131745	Respiratory system	negative	negative	Positive	Positive	negative	negative
280	1556	Mbab0891	M abscessus subsp bolletii	465	GCA_900134995	Respiratory system	negative	negative	Positive	Positive	negative	negative
281	1552	Mbab0857	M abscessus subsp bolletii	225	GCA_900131095	Respiratory system	negative	negative	Positive	Positive	negative	negative
282	1566	Mbab0878	M abscessus subsp bolletii	363	GCA_900132745	Respiratory system	negative	negative	Positive	Positive	negative	negative
283	1573	Mbab0881	M abscessus subsp bolletii	390	GCA_900133415	Respiratory system	negative	negative	Positive	Positive	negative	negative
284	1574	Mbab0882	M abscessus subsp bolletii	405	GCA_900133925	Respiratory system	negative	negative	Positive	Positive	negative	negative
285	1555	Mbab0887	M abscessus subsp bolletii	453	GCA_900134845	Respiratory system	negative	negative	Positive	Positive	negative	negative
286	1551	Mbab0855	M abscessus subsp bolletii	218	GCA_900131035	Respiratory system	negative	negative	Positive	Positive	negative	negative
287	1567	Mbab0874	M abscessus subsp bolletii	327	GCA_900132405	Respiratory system	negative	negative	Positive	Positive	negative	negative
288	1554	Mbab0871	M abscessus subsp bolletii	317	GCA_900132305	Respiratory system	negative	negative	Positive	Positive	negative	negative
289	1553	Mbab0869	M abscessus subsp bolletii	313	GCA_900132145	Respiratory system	negative	negative	Positive	Positive	negative	negative
290	1568	Mbab0849	M abscessus subsp bolletii	146	GCA_900130295	Respiratory system	negative	negative	Positive	Positive	negative	negative
291	1563	Mbab0853	M abscessus subsp bolletii	191	GCA_900130735	Respiratory system	negative	negative	Positive	Positive	negative	negative
292	1617	Mbab0876	M abscessus subsp bolletii	342	GCA_900132715	Respiratory system	negative	negative	Positive	Positive	negative	negative
293	1618	Mbab0860	M abscessus subsp bolletii	234	GCA_900131215	Respiratory system	negative	negative	Positive	Positive	negative	negative
294	1619	Mbab0866	M abscessus subsp bolletii	288	GCA_900131885	Respiratory system	negative	negative	Positive	Positive	negative	negative
295	1621	Mbab0856	M abscessus subsp bolletii	224	GCA_900131105	Respiratory system	negative	negative	Positive	Positive	negative	negative
296	1604	Mbab0886	M abscessus subsp bolletii	449	GCA_900134785	Respiratory system	negative	negative	Positive	Positive	negative	negative
297	1605	Mbab0890	M abscessus subsp bolletii	459	GCA_900134905	Respiratory system	negative	negative	Positive	Positive	negative	negative
298	1624	Mbab1487	M abscessus	M24	GCA_000260615	bronchoalveolar lavage	negative	Positive	Positive	Positive	negative	negative
299	1609	Mbab0863	M abscessus subsp bolletii	265	GCA_900131555	Respiratory system	negative	negative	Positive	Positive	negative	negative
300	1610	Mbab0850	M abscessus subsp bolletii	171	GCA_900130485	Respiratory system	negative	negative	Positive	Positive	negative	negative
301	1603	Mbab0892	M abscessus subsp bolletii	479	GCA_900138865	Respiratory system	negative	negative	Positive	Positive	negative	negative
302	1596	Mbab0842	M abscessus subsp bolletii	36	GCA_900135235	Respiratory system	negative	negative	Positive	Positive	negative	negative
303	1597	Mbab0843	M abscessus subsp bolletii	37	GCA_900135225	Respiratory system	negative	negative	Positive	Positive	negative	negative
304	1598	Mbab1352	M abscessus	FLAC003	GCA_001606195	Homo sapiens	negative	negative	Positive	Positive	negative	negative
305	1586	Mbab0895	M abscessus subsp bolletii	515	GCA_900140075	Respiratory system	negative	Positive	Positive	Positive	negative	negative
306	1601	Mbab0848	M abscessus subsp bolletii	138	GCA_900139635	Respiratory system	negative	negative	Positive	Positive	negative	negative
307	1642	Mbab0859	M abscessus subsp bolletii	228	GCA_900131135	Respiratory system	negative	negative	Positive	Positive	negative	negative
308	1643	Mbab0858	M abscessus subsp bolletii	227	GCA_900131125	Respiratory system	negative	negative	Positive	Positive	negative	negative
309	1640	Mbab0896	M abscessus subsp bolletii	516	GCA_900140135	Respiratory system	negative	negative	Positive	Positive	negative	negative
310	1645	Mbab0901	M abscessus subsp bolletii	542	GCA_900137935	Respiratory system	negative	negative	Positive	Positive	negative	negative
311	1641	Mbab1476	M abscessus	GO_160526	GCA_003068665	mammary gland tissue	negative	negative	Positive	negative	negative	negative
312	1644	Mbab1498	M abscessus	NCTC10882	GCA_900453325	Not available	negative	negative	Positive	Positive	negative	negative
313	1654	Mbab0950	M abscessus subsp bolletii	CIP108541	GCA_002086525	Not available	negative	negative	Positive	Positive	negative	negative
314	1653	Mbab0949	M abscessus subsp bolletii	BD	GCA_003609715	Not available	negative	negative	Positive	Positive	negative	negative
315	1663	Mbab0902	M abscessus subsp bolletii	695	GCA_900141195	Respiratory system	negative	negative	Positive	Positive	negative	negative
316	1613	Mbab0893	M abscessus subsp bolletii	511	GCA_900140055	Respiratory system	negative	negative	Positive	negative	negative	negative

317	1612	Mbab0897	M abscessus subsp bolletii	523	GCA_900140175	Respiratory system	negative	negative	Positive	negative	negative	negative
318	1611	Mbab0879	M abscessus subsp bolletii	366	GCA_900132765	Respiratory system	negative	negative	Positive	Positive	negative	negative
#N/A	700	Mbaa0001	M abscessus subsp abscessus	1	GCA_900139315	Respiratory system	Positive	Positive	negative	negative	negative	negative
#N/A	986	Mbaa0002	M abscessus subsp abscessus	2	GCA_002802325	sputum	Positive	Positive	negative	negative	negative	negative
#N/A	1023	Mbaa0004	M abscessus subsp abscessus	3	GCA_002802815	sputum	Positive	Positive	negative	negative	negative	negative
#N/A	839	Mbaa0005	M abscessus subsp abscessus	4	GCA_900139405	Respiratory system	Positive	Positive	negative	negative	negative	negative
#N/A	435	Mbaa0006	M abscessus subsp abscessus	5	GCA_900139465	Respiratory system	Positive	Positive	negative	negative	negative	negative
#N/A	699	Mbaa0007	M abscessus subsp abscessus	7	GCA_900139455	Respiratory system	Positive	Positive	negative	negative	negative	negative
#N/A	91	Mbaa0008	M abscessus subsp abscessus	8	GCA_900139425	Respiratory system	Positive	Positive	negative	negative	negative	negative
#N/A	196	Mbaa0009	M abscessus subsp abscessus	9	GCA_002802255	lavage fluid	Positive	Positive	negative	negative	negative	negative
#N/A	705	Mbaa0010	M abscessus subsp abscessus	9	GCA_900139495	Respiratory system	Positive	Positive	negative	negative	negative	negative
#N/A	56	Mbaa0011	M abscessus subsp abscessus	10	GCA_900139345	Respiratory system	Positive	Positive	negative	negative	negative	negative
#N/A	749	Mbaa0012	M abscessus subsp abscessus	11	GCA_900139325	Respiratory system	Positive	Positive	negative	negative	negative	negative
#N/A	351	Mbaa0013	M abscessus subsp abscessus	13	GCA_900139365	Respiratory system	Positive	Positive	negative	negative	negative	negative
#N/A	974	Mbaa0014	M abscessus subsp abscessus	14	GCA_002801565	sputum	negative	Positive	negative	negative	negative	negative
#N/A	251	Mbaa0015	M abscessus subsp abscessus	14	GCA_900139335	Respiratory system	Positive	Positive	negative	negative	negative	negative
#N/A	836	Mbaa0016	M abscessus subsp abscessus	15	GCA_900139375	Respiratory system	Positive	Positive	negative	negative	negative	negative
#N/A	95	Mbaa0018	M abscessus subsp abscessus	18	GCA_900139415	Respiratory system	Positive	Positive	negative	negative	negative	negative
#N/A	754	Mbaa0022	M abscessus subsp abscessus	21	GCA_900135185	Respiratory system	Positive	Positive	negative	negative	negative	negative
#N/A	745	Mbaa0024	M abscessus subsp abscessus	22	GCA_900135135	Respiratory system	Positive	Positive	negative	negative	negative	negative
#N/A	668	Mbaa0025	M abscessus subsp abscessus	23	GCA_900135125	Respiratory system	Positive	Positive	negative	negative	negative	negative
#N/A	957	Mbaa0026	M abscessus subsp abscessus	24	GCA_002801695	sputum	Positive	Positive	negative	negative	negative	negative
#N/A	418	Mbaa0027	M abscessus subsp abscessus	24	GCA_900135145	Respiratory system	Positive	Positive	negative	negative	negative	negative
#N/A	497	Mbaa0028	M abscessus subsp abscessus	25	GCA_900135195	Respiratory system	Positive	Positive	negative	negative	negative	negative
#N/A	964	Mbaa0030	M abscessus subsp abscessus	28	GCA_002801535	sputum	Positive	Positive	negative	negative	negative	negative
#N/A	434	Mbaa0031	M abscessus subsp abscessus	28	GCA_900135215	Respiratory system	negative	Positive	negative	negative	negative	negative
#N/A	1012	Mbaa0032	M abscessus subsp abscessus	29	GCA_002802775	lavage fluid	Positive	Positive	negative	negative	negative	negative
#N/A	416	Mbaa0033	M abscessus subsp abscessus	30	GCA_900136595	Respiratory system	Positive	Positive	negative	negative	negative	negative
#N/A	542	Mbaa0034	M abscessus subsp abscessus	31	GCA_900136625	Respiratory system	Positive	Positive	negative	negative	negative	negative
#N/A	420	Mbaa0035	M abscessus subsp abscessus	32	GCA_900136615	Respiratory system	Positive	Positive	negative	negative	negative	negative
#N/A	423	Mbaa0036	M abscessus subsp abscessus	33	GCA_900135205	Respiratory system	Positive	Positive	negative	negative	negative	negative
#N/A	421	Mbaa0037	M abscessus subsp abscessus	34	GCA_900135165	Respiratory system	Positive	Positive	negative	negative	negative	negative
#N/A	422	Mbaa0038	M abscessus subsp abscessus	35	GCA_900134095	Respiratory system	Positive	Positive	negative	negative	negative	negative
#N/A	675	Mbaa0039	M abscessus subsp abscessus	38	GCA_900135245	Respiratory system	Positive	Positive	negative	negative	negative	negative
#N/A	678	Mbaa0040	M abscessus subsp abscessus	39	GCA_900135265	Respiratory system	Positive	Positive	negative	negative	negative	negative
#N/A	823	Mbaa0042	M abscessus subsp abscessus	41	GCA_900135275	Respiratory system	Positive	Positive	negative	negative	negative	negative
#N/A	409	Mbaa0043	M abscessus subsp abscessus	43	GCA_900135295	Respiratory system	Positive	Positive	negative	negative	negative	negative
#N/A	406	Mbaa0045	M abscessus subsp abscessus	45	GCA_900135315	Respiratory system	Positive	Positive	negative	negative	negative	negative
#N/A	127	Mbaa0046	M abscessus subsp abscessus	46	GCA_900135325	Respiratory system	Positive	Positive	negative	negative	negative	negative
#N/A	128	Mbaa0047	M abscessus subsp abscessus	47	GCA_900135335	Respiratory system	Positive	Positive	negative	negative	negative	negative
#N/A	129	Mbaa0048	M abscessus subsp abscessus	49	GCA_900135355	Respiratory system	Positive	Positive	negative	negative	negative	negative
#N/A	130	Mbaa0049	M abscessus subsp abscessus	50	GCA_900135365	Respiratory system	Positive	Positive	negative	negative	negative	negative
#N/A	131	Mbaa0050	M abscessus subsp abscessus	51	GCA_900135345	Respiratory system	Positive	Positive	negative	negative	negative	negative
#N/A	132	Mbaa0051	M abscessus subsp abscessus	52	GCA_900134085	Respiratory system	Positive	Positive	negative	negative	negative	negative
#N/A	157	Mbaa0052	M abscessus subsp abscessus	53	GCA_900134115	Respiratory system	Positive	Positive	negative	negative	negative	negative
#N/A	148	Mbaa0053	M abscessus subsp abscessus	54	GCA_900135375	Respiratory system	Positive	Positive	negative	negative	negative	negative
#N/A	149	Mbaa0054	M abscessus subsp abscessus	55	GCA_900135385	Respiratory system	Positive	Positive	negative	negative	negative	negative
#N/A	133	Mbaa0055	M abscessus subsp abscessus	56	GCA_900135395	Respiratory system	Positive	Positive	negative	negative	negative	negative
#N/A	998	Mbaa0056	M abscessus subsp abscessus	57	GCA_002801405	sputum	Positive	Positive	negative	negative	negative	negative
#N/A	574	Mbaa0057	M abscessus subsp abscessus	61	GCA_900135435	Respiratory system	Positive	Positive	negative	negative	negative	negative
#N/A	177	Mbaa0058	M abscessus subsp abscessus	62	GCA_900135415	Respiratory system	Positive	Positive	negative	negative	negative	negative

#N/A	172	Mbaa0059	M abscessus subsp abscessus	63	GCA_900135455	Respiratory system	Positive	Positive	negative	negative	negative	negative
#N/A	176	Mbaa0060	M abscessus subsp abscessus	64	GCA_900135465	Respiratory system	Positive	Positive	negative	negative	negative	negative
#N/A	166	Mbaa0061	M abscessus subsp abscessus	65	GCA_900135495	Respiratory system	Positive	Positive	negative	negative	negative	negative
#N/A	160	Mbaa0062	M abscessus subsp abscessus	66	GCA_900135485	Respiratory system	Positive	Positive	negative	negative	negative	negative
#N/A	167	Mbaa0063	M abscessus subsp abscessus	67	GCA_900135515	Respiratory system	Positive	Positive	negative	negative	negative	negative
#N/A	168	Mbaa0064	M abscessus subsp abscessus	68	GCA_900135475	Respiratory system	Positive	Positive	negative	negative	negative	negative
#N/A	169	Mbaa0065	M abscessus subsp abscessus	69	GCA_900135505	Respiratory system	Positive	Positive	negative	negative	negative	negative
#N/A	170	Mbaa0066	M abscessus subsp abscessus	70	GCA_900135535	Respiratory system	Positive	Positive	negative	negative	negative	negative
#N/A	161	Mbaa0067	M abscessus subsp abscessus	71	GCA_900135525	Respiratory system	Positive	Positive	negative	negative	negative	negative
#N/A	134	Mbaa0069	M abscessus subsp abscessus	73	GCA_900134125	Respiratory system	Positive	Positive	negative	negative	negative	negative
#N/A	575	Mbaa0071	M abscessus subsp abscessus	77	GCA_900134145	Respiratory system	Positive	Positive	negative	negative	negative	negative
#N/A	576	Mbaa0072	M abscessus subsp abscessus	78	GCA_900134135	Respiratory system	Positive	Positive	negative	negative	negative	negative
#N/A	544	Mbaa0074	M abscessus subsp abscessus	85	GCA_900139435	Respiratory system	Positive	Positive	negative	negative	negative	negative
#N/A	307	Mbaa0076	M abscessus subsp abscessus	87	GCA_900139485	Respiratory system	Positive	Positive	negative	negative	negative	negative
#N/A	759	Mbaa0077	M abscessus subsp abscessus	88	GCA_900139475	Respiratory system	Positive	Positive	negative	negative	negative	negative
#N/A	785	Mbaa0079	M abscessus subsp abscessus	94	GCA_900135635	Respiratory system	Positive	Positive	negative	negative	negative	negative
#N/A	772	Mbaa0080	M abscessus subsp abscessus	95	GCA_900135545	Respiratory system	Positive	Positive	negative	negative	negative	negative
#N/A	687	Mbaa0081	M abscessus subsp abscessus	96	GCA_900135565	Respiratory system	Positive	Positive	negative	negative	negative	negative
#N/A	342	Mbaa0082	M abscessus subsp abscessus	97	GCA_900136645	Respiratory system	Positive	Positive	negative	negative	negative	negative
#N/A	433	Mbaa0083	M abscessus subsp abscessus	98	GCA_900136675	Respiratory system	Positive	Positive	negative	negative	negative	negative
#N/A	717	Mbaa0084	M abscessus subsp abscessus	100	GCA_900135585	Respiratory system	Positive	Positive	negative	negative	negative	negative
#N/A	718	Mbaa0086	M abscessus subsp abscessus	102	GCA_900135595	Respiratory system	Positive	Positive	negative	negative	negative	negative
#N/A	719	Mbaa0088	M abscessus subsp abscessus	104	GCA_900135625	Respiratory system	Positive	Positive	negative	negative	negative	negative
#N/A	720	Mbaa0089	M abscessus subsp abscessus	105	GCA_900135605	Respiratory system	Positive	Positive	negative	negative	negative	negative
#N/A	848	Mbaa0090	M abscessus subsp abscessus	106	GCA_900136665	Respiratory system	Positive	Positive	negative	negative	Positive	negative
#N/A	830	Mbaa0092	M abscessus subsp abscessus	109	GCA_900136705	Respiratory system	Positive	Positive	negative	negative	negative	negative
#N/A	265	Mbaa0093	M abscessus subsp abscessus	110	GCA_900136695	Respiratory system	Positive	Positive	negative	negative	negative	negative
#N/A	193	Mbaa0094	M abscessus subsp abscessus	113	GCA_002800765	lavage fluid	Positive	Positive	negative	negative	negative	negative
#N/A	762	Mbaa0095	M abscessus subsp abscessus	117	GCA_900136745	Respiratory system	Positive	Positive	negative	negative	negative	negative
#N/A	252	Mbaa0096	M abscessus subsp abscessus	119	GCA_900136765	Respiratory system	Positive	Positive	negative	negative	negative	negative
#N/A	432	Mbaa0097	M abscessus subsp abscessus	120	GCA_900136775	Respiratory system	Positive	Positive	negative	negative	negative	negative
#N/A	572	Mbaa0098	M abscessus subsp abscessus	121	GCA_900136795	Respiratory system	Positive	Positive	negative	negative	negative	negative
#N/A	984	Mbaa0100	M abscessus subsp abscessus	123	GCA_002801525	sputum	Positive	Positive	negative	negative	negative	negative
#N/A	443	Mbaa0101	M abscessus subsp abscessus	125	GCA_900136805	Respiratory system	Positive	Positive	negative	negative	negative	negative
#N/A	444	Mbaa0102	M abscessus subsp abscessus	126	GCA_900136845	Respiratory system	Positive	Positive	negative	negative	negative	negative
#N/A	445	Mbaa0103	M abscessus subsp abscessus	127	GCA_900136835	Respiratory system	Positive	Positive	negative	negative	negative	negative
#N/A	446	Mbaa0104	M abscessus subsp abscessus	128	GCA_900136855	Respiratory system	Positive	Positive	negative	negative	negative	negative
#N/A	874	Mbaa0106	M abscessus subsp abscessus	129	GCA_900139505	Respiratory system	Positive	Positive	negative	negative	negative	negative
#N/A	875	Mbaa0108	M abscessus subsp abscessus	136	GCA_900139565	Respiratory system	Positive	Positive	negative	negative	negative	negative
#N/A	880	Mbaa0110	M abscessus subsp abscessus	141	GCA_900136885	Respiratory system	Positive	Positive	negative	negative	negative	negative
#N/A	222	Mbaa0111	M abscessus subsp abscessus	142	GCA_900136865	Respiratory system	Positive	Positive	negative	negative	negative	negative
#N/A	385	Mbaa0112	M abscessus subsp abscessus	143	GCA_900136905	Respiratory system	Positive	Positive	negative	negative	negative	negative
#N/A	750	Mbaa0113	M abscessus subsp abscessus	144	GCA_900136895	Respiratory system	negative	Positive	negative	negative	negative	negative
#N/A	838	Mbaa0115	M abscessus subsp abscessus	148	GCA_900130315	Respiratory system	Positive	Positive	negative	negative	negative	negative
#N/A	694	Mbaa0116	M abscessus subsp abscessus	157	GCA_900130245	Respiratory system	Positive	Positive	negative	negative	negative	negative
#N/A	709	Mbaa0117	M abscessus subsp abscessus	160	GCA_900130395	Respiratory system	Positive	Positive	negative	negative	negative	negative
#N/A	715	Mbaa0118	M abscessus subsp abscessus	161	GCA_900130255	Respiratory system	Positive	Positive	negative	negative	negative	negative
#N/A	710	Mbaa0119	M abscessus subsp abscessus	162	GCA_900130415	Respiratory system	Positive	Positive	negative	negative	negative	negative
#N/A	711	Mbaa0120	M abscessus subsp abscessus	163	GCA_900130425	Respiratory system	Positive	Positive	negative	negative	negative	negative
#N/A	531	Mbaa0122	M abscessus subsp abscessus	166	GCA_900130445	Respiratory system	Positive	Positive	negative	negative	negative	negative
#N/A	532	Mbaa0123	M abscessus subsp abscessus	170	GCA_900130475	Respiratory system	Positive	Positive	negative	negative	negative	negative
#N/A	739	Mbaa0125	M abscessus subsp abscessus	173	GCA_900130505	Respiratory system	Positive	Positive	negative	negative	negative	negative
#N/A	538	Mbaa0126	M abscessus subsp abscessus	175	GCA_900130525	Respiratory system	Positive	Positive	negative	negative	negative	negative

#N/A	458	Mbaa0131	M abscessus subsp abscessus	188	GCA_900130655	Respiratory system	Positive	Positive	negative	negative	negative	negative
#N/A	486	Mbaa0132	M abscessus subsp abscessus	189	GCA_900130645	Respiratory system	Positive	Positive	negative	negative	negative	negative
#N/A	529	Mbaa0133	M abscessus subsp abscessus	194	GCA_900140315	Respiratory system	Positive	Positive	negative	negative	negative	negative
#N/A	527	Mbaa0134	M abscessus subsp abscessus	195	GCA_900140345	Respiratory system	Positive	Positive	negative	negative	negative	negative
#N/A	530	Mbaa0136	M abscessus subsp abscessus	199	GCA_900130785	Respiratory system	Positive	Positive	negative	negative	negative	negative
#N/A	704	Mbaa0138	M abscessus subsp abscessus	205	GCA_900130845	Respiratory system	Positive	Positive	negative	negative	negative	negative
#N/A	528	Mbaa0140	M abscessus subsp abscessus	210	GCA_900130875	Respiratory system	Positive	Positive	negative	negative	negative	negative
#N/A	716	Mbaa0141	M abscessus subsp abscessus	212	GCA_900130895	Respiratory system	Positive	Positive	negative	negative	negative	negative
#N/A	898	Mbaa0142	M abscessus subsp abscessus	213	GCA_002801215	lavage fluid	Positive	Positive	negative	negative	negative	negative
#N/A	400	Mbaa0145	M abscessus subsp abscessus	215	GCA_900130925	Respiratory system	Positive	Positive	negative	negative	negative	negative
#N/A	78	Mbaa0146	M abscessus subsp abscessus	216	GCA_002800715	sputum	Positive	Positive	negative	negative	negative	negative
#N/A	703	Mbaa0147	M abscessus subsp abscessus	216	GCA_900130955	Respiratory system	Positive	Positive	negative	negative	negative	negative
#N/A	990	Mbaa0149	M abscessus subsp abscessus	220	GCA_002800945	sputum	Positive	Positive	negative	negative	negative	negative
#N/A	790	Mbaa0150	M abscessus subsp abscessus	221	GCA_900131055	Respiratory system	Positive	Positive	negative	negative	negative	negative
#N/A	341	Mbaa0151	M abscessus subsp abscessus	222	GCA_002801985	lavage fluid	Positive	Positive	negative	negative	negative	negative
#N/A	723	Mbaa0152	M abscessus subsp abscessus	222	GCA_900131085	Respiratory system	Positive	Positive	negative	negative	negative	negative
#N/A	200	Mbaa0154	M abscessus subsp abscessus	227	GCA_002800725	sputum	Positive	Positive	negative	negative	negative	negative
#N/A	454	Mbaa0155	M abscessus subsp abscessus	229	GCA_900131145	Respiratory system	Positive	Positive	negative	negative	negative	negative
#N/A	462	Mbaa0156	M abscessus subsp abscessus	230	GCA_900131155	Respiratory system	Positive	Positive	negative	negative	negative	negative
#N/A	533	Mbaa0157	M abscessus subsp abscessus	231	GCA_900131165	Respiratory system	Positive	Positive	negative	negative	negative	negative
#N/A	453	Mbaa0158	M abscessus subsp abscessus	232	GCA_900131175	Respiratory system	Positive	Positive	negative	negative	negative	negative
#N/A	988	Mbaa0160	M abscessus subsp abscessus	234	GCA_002800955	sputum	Positive	Positive	negative	negative	negative	negative
#N/A	724	Mbaa0162	M abscessus subsp abscessus	239	GCA_900131245	Respiratory system	Positive	Positive	negative	negative	negative	negative
#N/A	732	Mbaa0166	M abscessus subsp abscessus	248	GCA_900140375	Respiratory system	Positive	Positive	negative	negative	negative	negative
#N/A	463	Mbaa0167	M abscessus subsp abscessus	250	GCA_900131335	Respiratory system	Positive	Positive	negative	negative	negative	negative
#N/A	465	Mbaa0168	M abscessus subsp abscessus	251	GCA_900131345	Respiratory system	Positive	Positive	negative	negative	negative	negative
#N/A	464	Mbaa0169	M abscessus subsp abscessus	252	GCA_900131355	Respiratory system	Positive	Positive	negative	negative	negative	negative
#N/A	456	Mbaa0170	M abscessus subsp abscessus	253	GCA_900131365	Respiratory system	Positive	Positive	negative	negative	negative	negative
#N/A	735	Mbaa0172	M abscessus subsp abscessus	254	GCA_900131375	Respiratory system	Positive	Positive	negative	negative	negative	negative
#N/A	362	Mbaa0177	M abscessus subsp abscessus	267	GCA_900140355	Respiratory system	Positive	Positive	negative	negative	negative	negative
#N/A	734	Mbaa0178	M abscessus subsp abscessus	269	GCA_900131575	Respiratory system	Positive	Positive	negative	negative	negative	negative
#N/A	812	Mbaa0179	M abscessus subsp abscessus	270	GCA_900131595	Respiratory system	Positive	Positive	negative	negative	negative	negative
#N/A	363	Mbaa0180	M abscessus subsp abscessus	272	GCA_900131605	Respiratory system	Positive	Positive	negative	negative	negative	negative
#N/A	364	Mbaa0181	M abscessus subsp abscessus	273	GCA_900131625	Respiratory system	Positive	Positive	negative	negative	negative	negative
#N/A	689	Mbaa0183	M abscessus subsp abscessus	279	GCA_900140395	Respiratory system	Positive	Positive	negative	negative	negative	negative
#N/A	336	Mbaa0184	M abscessus subsp abscessus	280	GCA_002800365	lavage fluid	Positive	Positive	negative	negative	negative	negative
#N/A	365	Mbaa0187	M abscessus subsp abscessus	283	GCA_900131705	Respiratory system	Positive	Positive	negative	negative	negative	negative
#N/A	522	Mbaa0188	M abscessus subsp abscessus	285	GCA_900131715	Respiratory system	Positive	Positive	negative	negative	negative	negative
#N/A	375	Mbaa0192	M abscessus subsp abscessus	293	GCA_900131925	Respiratory system	Positive	Positive	negative	negative	negative	negative
#N/A	457	Mbaa0193	M abscessus subsp abscessus	296	GCA_900131965	Respiratory system	Positive	Positive	negative	negative	negative	negative
#N/A	516	Mbaa0194	M abscessus subsp abscessus	297	GCA_900132005	Respiratory system	Positive	Positive	negative	negative	negative	negative
#N/A	525	Mbaa0195	M abscessus subsp abscessus	298	GCA_900131975	Respiratory system	Positive	Positive	negative	negative	negative	negative
#N/A	523	Mbaa0196	M abscessus subsp abscessus	299	GCA_900132015	Respiratory system	Positive	Positive	negative	negative	negative	negative
#N/A	466	Mbaa0197	M abscessus subsp abscessus	302	GCA_900131985	Respiratory system	Positive	Positive	negative	negative	negative	negative
#N/A	577	Mbaa0198	M abscessus subsp abscessus	304	GCA_900132045	Respiratory system	Positive	Positive	negative	negative	negative	negative
#N/A	698	Mbaa0199	M abscessus subsp abscessus	311	GCA_900132115	Respiratory system	Positive	Positive	negative	negative	negative	negative
#N/A	376	Mbaa0201	M abscessus subsp abscessus	315	GCA_900132155	Respiratory system	Positive	Positive	negative	negative	negative	negative
#N/A	517	Mbaa0202	M abscessus subsp abscessus	318	GCA_900132315	Respiratory system	Positive	Positive	negative	negative	negative	negative
#N/A	701	Mbaa0203	M abscessus subsp abscessus	319	GCA_900132335	Respiratory system	Positive	Positive	negative	negative	negative	negative
#N/A	459	Mbaa0204	M abscessus subsp abscessus	320	GCA_900132325	Respiratory system	Positive	Positive	negative	negative	negative	negative
#N/A	366	Mbaa0205	M abscessus subsp abscessus	323	GCA_900132365	Respiratory system	Positive	Positive	negative	negative	negative	negative
#N/A	736	Mbaa0206	M abscessus subsp abscessus	328	GCA_900132415	Respiratory system	Positive	Positive	negative	negative	negative	negative
#N/A	693	Mbaa0208	M abscessus subsp abscessus	330	GCA_900132435	Respiratory system	Positive	Positive	negative	negative	negative	negative

#N/A	518	Mbaa0209	M abscessus subsp abscessus	332	GCA_900132445	Respiratory system	Positive	Positive	negative	negative	negative	negative
#N/A	45	Mbaa0210	M abscessus subsp abscessus	333	GCA_900132425	Respiratory system	Positive	Positive	negative	negative	negative	negative
#N/A	377	Mbaa0211	M abscessus subsp abscessus	336	GCA_900132505	Respiratory system	Positive	Positive	negative	negative	negative	negative
#N/A	695	Mbaa0212	M abscessus subsp abscessus	337	GCA_900132835	Respiratory system	Positive	Positive	negative	negative	negative	negative
#N/A	367	Mbaa0213	M abscessus subsp abscessus	338	GCA_900140405	Respiratory system	Positive	Positive	negative	negative	negative	negative
#N/A	368	Mbaa0214	M abscessus subsp abscessus	339	GCA_900132865	Respiratory system	Positive	Positive	negative	negative	negative	negative
#N/A	447	Mbaa0216	M abscessus subsp abscessus	341	GCA_900132615	Respiratory system	Positive	Positive	negative	negative	negative	negative
#N/A	767	Mbaa0218	M abscessus subsp abscessus	345	GCA_900132805	Respiratory system	Positive	Positive	negative	negative	negative	negative
#N/A	714	Mbaa0219	M abscessus subsp abscessus	348	GCA_900132845	Respiratory system	Positive	Positive	negative	negative	negative	negative
#N/A	369	Mbaa0220	M abscessus subsp abscessus	349	GCA_900135105	Respiratory system	Positive	Positive	negative	negative	negative	negative
#N/A	811	Mbaa0221	M abscessus subsp abscessus	350	GCA_900132595	Respiratory system	Positive	Positive	negative	negative	negative	negative
#N/A	460	Mbaa0222	M abscessus subsp abscessus	351	GCA_900132585	Respiratory system	Positive	Positive	negative	negative	negative	negative
#N/A	32	Mbaa0223	M abscessus subsp abscessus	353	GCA_900132655	Respiratory system	Positive	Positive	negative	negative	negative	negative
#N/A	696	Mbaa0225	M abscessus subsp abscessus	357	GCA_900132675	Respiratory system	Positive	Positive	negative	negative	negative	negative
#N/A	729	Mbaa0226	M abscessus subsp abscessus	358	GCA_900132685	Respiratory system	Positive	Positive	negative	negative	negative	negative
#N/A	733	Mbaa0227	M abscessus subsp abscessus	361	GCA_900132735	Respiratory system	Positive	Positive	negative	negative	negative	negative
#N/A	727	Mbaa0228	M abscessus subsp abscessus	364	GCA_900132755	Respiratory system	Positive	Positive	negative	negative	negative	negative
#N/A	519	Mbaa0229	M abscessus subsp abscessus	365	GCA_900133205	Respiratory system	Positive	Positive	negative	negative	negative	negative
#N/A	728	Mbaa0230	M abscessus subsp abscessus	367	GCA_900133225	Respiratory system	Positive	Positive	negative	negative	negative	negative
#N/A	448	Mbaa0231	M abscessus subsp abscessus	370	GCA_900133255	Respiratory system	Positive	Positive	negative	negative	negative	negative
#N/A	358	Mbaa0234	M abscessus subsp abscessus	373	GCA_900133285	Respiratory system	Positive	Positive	negative	negative	negative	negative
#N/A	88	Mbaa0236	M abscessus subsp abscessus	375	GCA_900133305	Respiratory system	Positive	Positive	negative	negative	negative	negative
#N/A	520	Mbaa0237	M abscessus subsp abscessus	376	GCA_900138985	Respiratory system	Positive	Positive	negative	negative	negative	negative
#N/A	378	Mbaa0238	M abscessus subsp abscessus	378	GCA_900133315	Respiratory system	Positive	Positive	negative	negative	negative	negative
#N/A	712	Mbaa0240	M abscessus subsp abscessus	380	GCA_900133375	Respiratory system	Positive	Positive	negative	negative	negative	negative
#N/A	524	Mbaa0241	M abscessus subsp abscessus	381	GCA_900133355	Respiratory system	Positive	Positive	negative	negative	negative	negative
#N/A	721	Mbaa0242	M abscessus subsp abscessus	382	GCA_900133365	Respiratory system	Positive	Positive	negative	negative	negative	negative
#N/A	31	Mbaa0243	M abscessus subsp abscessus	384	GCA_900133345	Respiratory system	Positive	Positive	negative	negative	negative	negative
#N/A	343	Mbaa0244	M abscessus subsp abscessus	385	GCA_900133385	Respiratory system	Positive	Positive	negative	negative	negative	negative
#N/A	370	Mbaa0245	M abscessus subsp abscessus	386	GCA_900133465	Respiratory system	Positive	Positive	negative	negative	negative	negative
#N/A	503	Mbaa0246	M abscessus subsp abscessus	387	GCA_900133425	Respiratory system	Positive	Positive	Positive	negative	negative	negative
#N/A	504	Mbaa0247	M abscessus subsp abscessus	388	GCA_900133405	Respiratory system	Positive	Positive	Positive	negative	negative	negative
#N/A	536	Mbaa0248	M abscessus subsp abscessus	389	GCA_900133395	Respiratory system	Positive	Positive	negative	negative	negative	negative
#N/A	730	Mbaa0249	M abscessus subsp abscessus	391	GCA_900133435	Respiratory system	Positive	Positive	negative	negative	negative	negative
#N/A	725	Mbaa0250	M abscessus subsp abscessus	394	GCA_900133855	Respiratory system	Positive	Positive	negative	negative	negative	negative
#N/A	41	Mbaa0251	M abscessus subsp abscessus	397	GCA_900133845	Respiratory system	Positive	Positive	negative	negative	negative	negative
#N/A	90	Mbaa0252	M abscessus subsp abscessus	398	GCA_900133895	Respiratory system	Positive	Positive	negative	negative	negative	negative
#N/A	371	Mbaa0253	M abscessus subsp abscessus	399	GCA_900133885	Respiratory system	Positive	Positive	negative	negative	negative	negative
#N/A	379	Mbaa0254	M abscessus subsp abscessus	400	GCA_900133905	Respiratory system	Positive	Positive	negative	negative	negative	negative
#N/A	380	Mbaa0255	M abscessus subsp abscessus	401	GCA_900134065	Respiratory system	Positive	Positive	negative	negative	negative	negative
#N/A	697	Mbaa0256	M abscessus subsp abscessus	402	GCA_900133915	Respiratory system	Positive	Positive	negative	negative	negative	negative
#N/A	726	Mbaa0257	M abscessus subsp abscessus	403	GCA_900134055	Respiratory system	Positive	Positive	negative	negative	negative	negative
#N/A	766	Mbaa0258	M abscessus subsp abscessus	404	GCA_900134015	Respiratory system	Positive	Positive	negative	negative	negative	negative
#N/A	372	Mbaa0259	M abscessus subsp abscessus	406	GCA_900133955	Respiratory system	Positive	Positive	negative	negative	negative	negative
#N/A	381	Mbaa0260	M abscessus subsp abscessus	407	GCA_900133935	Respiratory system	Positive	Positive	negative	negative	negative	negative
#N/A	494	Mbaa0262	M abscessus subsp abscessus	409	GCA_900133945	Respiratory system	Positive	Positive	negative	negative	negative	negative
#N/A	36	Mbaa0263	M abscessus subsp abscessus	410	GCA_900133975	Respiratory system	Positive	Positive	negative	negative	negative	negative
#N/A	702	Mbaa0264	M abscessus subsp abscessus	413	GCA_900133965	Respiratory system	Positive	Positive	negative	negative	negative	negative
#N/A	356	Mbaa0265	M abscessus subsp abscessus	414	GCA_900133985	Respiratory system	Positive	Positive	negative	negative	negative	negative
#N/A	85	Mbaa0266	M abscessus subsp abscessus	415	GCA_900133995	Respiratory system	Positive	Positive	negative	negative	negative	negative
#N/A	361	Mbaa0267	M abscessus subsp abscessus	418	GCA_900134045	Respiratory system	Positive	Positive	negative	negative	negative	negative
#N/A	259	Mbaa0270	M abscessus subsp abscessus	421	GCA_900135045	Respiratory system	Positive	Positive	negative	negative	negative	negative
#N/A	236	Mbaa0271	M abscessus subsp abscessus	422	GCA_900135075	Respiratory system	Positive	Positive	negative	negative	negative	negative

#N/A	241	Mbaa0272	M abscessus subsp abscessus	423	GCA_900135065	Respiratory system	Positive	Positive	negative	negative	negative	negative
#N/A	237	Mbaa0273	M abscessus subsp abscessus	424	GCA_900135085	Respiratory system	Positive	Positive	negative	negative	negative	negative
#N/A	238	Mbaa0274	M abscessus subsp abscessus	425	GCA_900135095	Respiratory system	Positive	Positive	negative	negative	negative	negative
#N/A	239	Mbaa0275	M abscessus subsp abscessus	426	GCA_900134695	Respiratory system	Positive	Positive	negative	negative	negative	negative
#N/A	338	Mbaa0276	M abscessus subsp abscessus	427	GCA_900134775	Respiratory system	Positive	Positive	negative	negative	negative	negative
#N/A	731	Mbaa0279	M abscessus subsp abscessus	431	GCA_900135015	Respiratory system	Positive	Positive	negative	negative	negative	negative
#N/A	242	Mbaa0280	M abscessus subsp abscessus	433	GCA_900135025	Respiratory system	Positive	Positive	negative	negative	negative	negative
#N/A	255	Mbaa0281	M abscessus subsp abscessus	434	GCA_900135055	Respiratory system	Positive	Positive	negative	negative	negative	negative
#N/A	373	Mbaa0282	M abscessus subsp abscessus	435	GCA_900134705	Respiratory system	Positive	Positive	negative	negative	negative	negative
#N/A	382	Mbaa0283	M abscessus subsp abscessus	436	GCA_900134715	Respiratory system	Positive	Positive	negative	negative	negative	negative
#N/A	374	Mbaa0284	M abscessus subsp abscessus	437	GCA_900134745	Respiratory system	Positive	Positive	negative	negative	negative	negative
#N/A	722	Mbaa0286	M abscessus subsp abscessus	440	GCA_900134735	Respiratory system	Positive	Positive	negative	negative	negative	negative
#N/A	345	Mbaa0289	M abscessus subsp abscessus	446	GCA_900134795	Respiratory system	Positive	Positive	negative	negative	negative	negative
#N/A	86	Mbaa0291	M abscessus subsp abscessus	450	GCA_900134815	Respiratory system	Positive	Positive	negative	negative	negative	negative
#N/A	743	Mbaa0292	M abscessus subsp abscessus	454	GCA_900134875	Respiratory system	Positive	Positive	negative	negative	negative	negative
#N/A	344	Mbaa0293	M abscessus subsp abscessus	455	GCA_900134895	Respiratory system	Positive	Positive	negative	negative	negative	negative
#N/A	10	Mbaa0294	M abscessus subsp abscessus	456	GCA_900134855	Respiratory system	Positive	Positive	negative	negative	negative	negative
#N/A	37	Mbaa0295	M abscessus subsp abscessus	460	GCA_900134935	Respiratory system	Positive	Positive	negative	negative	negative	negative
#N/A	40	Mbaa0296	M abscessus subsp abscessus	461	GCA_900134945	Respiratory system	Positive	Positive	negative	negative	negative	negative
#N/A	8	Mbaa0298	M abscessus subsp abscessus	464	GCA_900134955	Respiratory system	Positive	Positive	negative	negative	negative	negative
#N/A	667	Mbaa0299	M abscessus subsp abscessus	467	GCA_900134975	Respiratory system	Positive	Positive	negative	negative	negative	negative
#N/A	115	Mbaa0300	M abscessus subsp abscessus	469	GCA_900136415	Respiratory system	Positive	Positive	negative	negative	negative	negative
#N/A	244	Mbaa0303	M abscessus subsp abscessus	473	GCA_900138815	Respiratory system	Positive	Positive	negative	negative	negative	negative
#N/A	346	Mbaa0305	M abscessus subsp abscessus	477	GCA_900138825	Respiratory system	Positive	Positive	negative	negative	negative	negative
#N/A	757	Mbaa0306	M abscessus subsp abscessus	480	GCA_900138885	Respiratory system	Positive	Positive	negative	negative	negative	negative
#N/A	756	Mbaa0307	M abscessus subsp abscessus	481	GCA_900138875	Respiratory system	Positive	Positive	negative	negative	negative	negative
#N/A	602	Mbaa0309	M abscessus subsp abscessus	486	GCA_900138935	Respiratory system	Positive	Positive	negative	negative	negative	negative
#N/A	821	Mbaa0310	M abscessus subsp abscessus	487	GCA_900138925	Respiratory system	Positive	Positive	negative	negative	negative	negative
#N/A	240	Mbaa0312	M abscessus subsp abscessus	489	GCA_900138965	Respiratory system	Positive	Positive	negative	negative	negative	negative
#N/A	306	Mbaa0313	M abscessus subsp abscessus	491	GCA_900136435	Respiratory system	Positive	Positive	negative	negative	negative	negative
#N/A	87	Mbaa0314	M abscessus subsp abscessus	493	GCA_900136445	Respiratory system	Positive	Positive	negative	negative	negative	negative
#N/A	820	Mbaa0315	M abscessus subsp abscessus	494	GCA_900136475	Respiratory system	Positive	Positive	negative	negative	negative	negative
#N/A	784	Mbaa0317	M abscessus subsp abscessus	500	GCA_900136515	Respiratory system	Positive	Positive	negative	negative	negative	negative
#N/A	106	Mbaa0319	M abscessus subsp abscessus	502	GCA_900136505	Respiratory system	Positive	Positive	negative	negative	negative	negative
#N/A	742	Mbaa0320	M abscessus subsp abscessus	503	GCA_900136555	Respiratory system	Positive	Positive	negative	negative	negative	negative
#N/A	93	Mbaa0321	M abscessus subsp abscessus	504	GCA_900136535	Respiratory system	Positive	Positive	negative	negative	negative	negative
#N/A	783	Mbaa0322	M abscessus subsp abscessus	505	GCA_900136575	Respiratory system	Positive	Positive	negative	negative	negative	negative
#N/A	284	Mbaa0324	M abscessus subsp abscessus	507	GCA_900136585	Respiratory system	Positive	Positive	negative	negative	negative	negative
#N/A	412	Mbaa0326	M abscessus subsp abscessus	509	GCA_900136605	Respiratory system	Positive	Positive	negative	negative	negative	negative
#N/A	763	Mbaa0327	M abscessus subsp abscessus	510	GCA_900135645	Respiratory system	Positive	Positive	negative	negative	negative	negative
#N/A	340	Mbaa0329	M abscessus subsp abscessus	514	GCA_900139875	Respiratory system	Positive	Positive	negative	negative	negative	negative
#N/A	332	Mbaa0330	M abscessus subsp abscessus	517	GCA_900140125	Respiratory system	Positive	Positive	negative	negative	negative	negative
#N/A	483	Mbaa0331	M abscessus subsp abscessus	518	GCA_900140115	Respiratory system	Positive	Positive	negative	negative	negative	negative
#N/A	866	Mbaa0332	M abscessus subsp abscessus	520	GCA_900140085	Respiratory system	Positive	Positive	negative	negative	negative	negative
#N/A	775	Mbaa0333	M abscessus subsp abscessus	521	GCA_900140105	Respiratory system	Positive	Positive	negative	negative	negative	negative
#N/A	670	Mbaa0335	M abscessus subsp abscessus	527	GCA_900140185	Respiratory system	Positive	Positive	negative	negative	negative	negative
#N/A	599	Mbaa0336	M abscessus subsp abscessus	529	GCA_900139785	Respiratory system	Positive	Positive	negative	negative	negative	negative
#N/A	235	Mbaa0337	M abscessus subsp abscessus	530	GCA_900140195	Respiratory system	Positive	Positive	negative	negative	negative	negative
#N/A	915	Mbaa0341	M abscessus subsp abscessus	539	GCA_900140005	Respiratory system	Positive	Positive	negative	negative	negative	negative
#N/A	912	Mbaa0342	M abscessus subsp abscessus	540	GCA_900140555	Respiratory system	Positive	Positive	negative	negative	negative	negative
#N/A	33	Mbaa0343	M abscessus subsp abscessus	541	GCA_900140675	Respiratory system	Positive	Positive	negative	negative	negative	negative
#N/A	713	Mbaa0344	M abscessus subsp abscessus	548	GCA_900136945	Respiratory system	Positive	Positive	negative	negative	negative	negative
#N/A	781	Mbaa0345	M abscessus subsp abscessus	549	GCA_900137955	Respiratory system	Positive	Positive	negative	negative	negative	negative

#N/A	61	Mbaa0347	M abscessus subsp abscessus	552	GCA_900136925	Respiratory system	Positive	Positive	negative	negative	negative	negative
#N/A	112	Mbaa0348	M abscessus subsp abscessus	553	GCA_900137965	Respiratory system	Positive	Positive	negative	negative	negative	negative
#N/A	758	Mbaa0349	M abscessus subsp abscessus	573	GCA_900141055	Respiratory system	Positive	Positive	negative	negative	negative	negative
#N/A	329	Mbaa0350	M abscessus subsp abscessus	574	GCA_900141175	Respiratory system	Positive	Positive	negative	negative	negative	negative
#N/A	125	Mbaa0353	M abscessus subsp abscessus	577	GCA_900140425	Respiratory system	Positive	Positive	negative	negative	negative	negative
#N/A	425	Mbaa0354	M abscessus subsp abscessus	578	GCA_900140465	Respiratory system	Positive	Positive	negative	negative	negative	negative
#N/A	885	Mbaa0355	M abscessus subsp abscessus	579	GCA_900140485	Respiratory system	Positive	Positive	negative	negative	negative	negative
#N/A	630	Mbaa0356	M abscessus subsp abscessus	580	GCA_900140475	Respiratory system	Positive	Positive	negative	negative	negative	negative
#N/A	662	Mbaa0357	M abscessus subsp abscessus	581	GCA_900140505	Respiratory system	Positive	Positive	negative	negative	negative	negative
#N/A	53	Mbaa0358	M abscessus subsp abscessus	582	GCA_900139025	Respiratory system	Positive	Positive	negative	negative	negative	negative
#N/A	57	Mbaa0359	M abscessus subsp abscessus	583	GCA_900140045	Respiratory system	Positive	Positive	negative	negative	negative	negative
#N/A	492	Mbaa0360	M abscessus subsp abscessus	584	GCA_900140455	Respiratory system	Positive	Positive	negative	negative	negative	negative
#N/A	46	Mbaa0361	M abscessus subsp abscessus	587	GCA_900140515	Respiratory system	Positive	Positive	negative	negative	negative	negative
#N/A	124	Mbaa0362	M abscessus subsp abscessus	588	GCA_900139035	Respiratory system	Positive	Positive	negative	negative	negative	negative
#N/A	482	Mbaa0363	M abscessus subsp abscessus	589	GCA_900140525	Respiratory system	Positive	Positive	negative	negative	negative	negative
#N/A	930	Mbaa0365	M abscessus subsp abscessus	591	GCA_900140545	Respiratory system	Positive	Positive	negative	negative	negative	negative
#N/A	391	Mbaa0367	M abscessus subsp abscessus	593	GCA_900139065	Respiratory system	Positive	Positive	negative	negative	negative	negative
#N/A	392	Mbaa0368	M abscessus subsp abscessus	594	GCA_900140565	Respiratory system	Positive	Positive	negative	negative	negative	negative
#N/A	636	Mbaa0369	M abscessus subsp abscessus	595	GCA_900140575	Respiratory system	Positive	Positive	negative	negative	negative	negative
#N/A	28	Mbaa0370	M abscessus subsp abscessus	596	GCA_900140595	Respiratory system	Positive	Positive	negative	negative	negative	negative
#N/A	247	Mbaa0371	M abscessus subsp abscessus	597	GCA_900140605	Respiratory system	Positive	Positive	negative	negative	negative	negative
#N/A	638	Mbaa0372	M abscessus subsp abscessus	599	GCA_900139055	Respiratory system	Positive	Positive	negative	negative	negative	negative
#N/A	47	Mbaa0373	M abscessus subsp abscessus	600	GCA_900140615	Respiratory system	Positive	Positive	negative	negative	negative	negative
#N/A	205	Mbaa0374	M abscessus subsp abscessus	601	GCA_900140625	Respiratory system	Positive	Positive	negative	negative	negative	negative
#N/A	113	Mbaa0375	M abscessus subsp abscessus	602	GCA_900140645	Respiratory system	Positive	Positive	negative	negative	negative	negative
#N/A	746	Mbaa0376	M abscessus subsp abscessus	604	GCA_900140665	Respiratory system	Positive	Positive	negative	negative	negative	negative
#N/A	916	Mbaa0377	M abscessus subsp abscessus	605	GCA_900139885	Respiratory system	Positive	Positive	negative	negative	negative	negative
#N/A	3	Mbaa0378	M abscessus subsp abscessus	606	GCA_900140685	Respiratory system	Positive	Positive	negative	negative	negative	negative
#N/A	505	Mbaa0379	M abscessus subsp abscessus	607	GCA_900140655	Respiratory system	Positive	Positive	Positive	negative	negative	negative
#N/A	48	Mbaa0382	M abscessus subsp abscessus	611	GCA_900140725	Respiratory system	Positive	Positive	negative	negative	negative	negative
#N/A	123	Mbaa0383	M abscessus subsp abscessus	612	GCA_900140705	Respiratory system	Positive	Positive	negative	negative	negative	negative
#N/A	917	Mbaa0384	M abscessus subsp abscessus	613	GCA_900140695	Respiratory system	Positive	Positive	negative	negative	negative	negative
#N/A	609	Mbaa0385	M abscessus subsp abscessus	614	GCA_900139735	Respiratory system	Positive	Positive	negative	negative	negative	negative
#N/A	206	Mbaa0386	M abscessus subsp abscessus	615	GCA_900140715	Respiratory system	Positive	Positive	negative	negative	negative	negative
#N/A	89	Mbaa0387	M abscessus subsp abscessus	617	GCA_900139085	Respiratory system	Positive	Positive	negative	negative	negative	negative
#N/A	122	Mbaa0388	M abscessus subsp abscessus	619	GCA_900140745	Respiratory system	Positive	Positive	negative	negative	negative	negative
#N/A	30	Mbaa0389	M abscessus subsp abscessus	621	GCA_900140785	Respiratory system	Positive	Positive	negative	negative	negative	negative
#N/A	7	Mbaa0390	M abscessus subsp abscessus	622	GCA_900140805	Respiratory system	Positive	Positive	negative	negative	negative	negative
#N/A	506	Mbaa0391	M abscessus subsp abscessus	623	GCA_900139765	Respiratory system	Positive	Positive	Positive	negative	negative	negative
#N/A	648	Mbaa0393	M abscessus subsp abscessus	626	GCA_900139955	Respiratory system	Positive	Positive	negative	negative	negative	negative
#N/A	120	Mbaa0396	M abscessus subsp abscessus	630	GCA_900140835	Respiratory system	Positive	Positive	negative	negative	negative	negative
#N/A	669	Mbaa0397	M abscessus subsp abscessus	632	GCA_900139685	Respiratory system	Positive	Positive	negative	negative	negative	negative
#N/A	13	Mbaa0398	M abscessus subsp abscessus	633	GCA_900140855	Respiratory system	Positive	Positive	negative	negative	negative	negative
#N/A	1007	Mbaa0399	M abscessus subsp abscessus	634	GCA_900140825	Respiratory system	Positive	Positive	negative	negative	negative	negative
#N/A	1003	Mbaa0400	M abscessus subsp abscessus	636	GCA_900140885	Respiratory system	Positive	Positive	negative	negative	negative	negative
#N/A	49	Mbaa0401	M abscessus subsp abscessus	638	GCA_900140905	Respiratory system	Positive	Positive	negative	negative	negative	negative
#N/A	4	Mbaa0402	M abscessus subsp abscessus	639	GCA_900140875	Respiratory system	Positive	Positive	negative	negative	negative	negative
#N/A	650	Mbaa0403	M abscessus subsp abscessus	640	GCA_900139695	Respiratory system	Positive	Positive	negative	negative	negative	negative
#N/A	674	Mbaa0404	M abscessus subsp abscessus	641	GCA_900139105	Respiratory system	Positive	Positive	negative	negative	negative	negative
#N/A	484	Mbaa0405	M abscessus subsp abscessus	643	GCA_900139805	Respiratory system	Positive	Positive	negative	negative	negative	negative
#N/A	249	Mbaa0406	M abscessus subsp abscessus	644	GCA_900139115	Respiratory system	Positive	Positive	negative	negative	negative	negative
#N/A	248	Mbaa0407	M abscessus subsp abscessus	645	GCA_900140925	Respiratory system	Positive	Positive	negative	negative	negative	negative
#N/A	673	Mbaa0408	M abscessus subsp abscessus	646	GCA_900140895	Respiratory system	Positive	Positive	negative	negative	negative	negative

#N/A	121	Mbaa0410	M abscessus subsp abscessus	649	GCA_900140915	Respiratory system	Positive	Positive	negative	negative	negative	negative
#N/A	647	Mbaa0411	M abscessus subsp abscessus	650	GCA_900140955	Respiratory system	Positive	Positive	negative	negative	negative	negative
#N/A	500	Mbaa0412	M abscessus subsp abscessus	652	GCA_900140945	Respiratory system	Positive	Positive	negative	negative	negative	negative
#N/A	266	Mbaa0413	M abscessus subsp abscessus	653	GCA_900139135	Respiratory system	Positive	Positive	negative	negative	negative	negative
#N/A	254	Mbaa0414	M abscessus subsp abscessus	654	GCA_900140935	Respiratory system	Positive	Positive	negative	negative	negative	negative
#N/A	253	Mbaa0415	M abscessus subsp abscessus	655	GCA_900140965	Respiratory system	Positive	Positive	negative	negative	negative	negative
#N/A	50	Mbaa0416	M abscessus subsp abscessus	656	GCA_900139125	Respiratory system	Positive	Positive	negative	negative	negative	negative
#N/A	631	Mbaa0417	M abscessus subsp abscessus	657	GCA_900140975	Respiratory system	Positive	Positive	negative	negative	negative	negative
#N/A	635	Mbaa0418	M abscessus subsp abscessus	658	GCA_900140985	Respiratory system	Positive	Positive	negative	negative	negative	negative
#N/A	195	Mbaa0419	M abscessus subsp abscessus	660	GCA_900140995	Respiratory system	Positive	Positive	negative	negative	negative	negative
#N/A	543	Mbaa0420	M abscessus subsp abscessus	661	GCA_900141005	Respiratory system	Positive	Positive	negative	negative	negative	negative
#N/A	135	Mbaa0421	M abscessus subsp abscessus	662	GCA_900139185	Respiratory system	Positive	Positive	negative	negative	negative	negative
#N/A	51	Mbaa0424	M abscessus subsp abscessus	667	GCA_900139935	Respiratory system	Positive	Positive	negative	negative	negative	negative
#N/A	1	Mbaa0425	M abscessus subsp abscessus	668	GCA_900139975	Respiratory system	Positive	Positive	negative	negative	negative	negative
#N/A	203	Mbaa0427	M abscessus subsp abscessus	670	GCA_900140015	Respiratory system	Positive	Positive	negative	negative	negative	negative
#N/A	390	Mbaa0428	M abscessus subsp abscessus	671	GCA_900141035	Respiratory system	Positive	Positive	negative	negative	negative	negative
#N/A	671	Mbaa0430	M abscessus subsp abscessus	673	GCA_900141065	Respiratory system	Positive	Positive	negative	negative	negative	negative
#N/A	81	Mbaa0433	M abscessus subsp abscessus	676	GCA_900139175	Respiratory system	Positive	Positive	negative	negative	negative	negative
#N/A	515	Mbaa0434	M abscessus subsp abscessus	678	GCA_900141075	Respiratory system	Positive	Positive	negative	negative	negative	negative
#N/A	663	Mbaa0436	M abscessus subsp abscessus	680	GCA_900141105	Respiratory system	Positive	Positive	negative	negative	negative	negative
#N/A	62	Mbaa0437	M abscessus subsp abscessus	683	GCA_900140285	Respiratory system	Positive	Positive	negative	negative	negative	negative
#N/A	611	Mbaa0438	M abscessus subsp abscessus	684	GCA_900141125	Respiratory system	Positive	Positive	negative	negative	negative	negative
#N/A	82	Mbaa0439	M abscessus subsp abscessus	685	GCA_900141095	Respiratory system	Positive	Positive	negative	negative	negative	negative
#N/A	397	Mbaa0440	M abscessus subsp abscessus	686	GCA_900140065	Respiratory system	Positive	Positive	negative	negative	negative	negative
#N/A	186	Mbaa0441	M abscessus subsp abscessus	687	GCA_900140265	Respiratory system	Positive	Positive	negative	negative	negative	negative
#N/A	114	Mbaa0442	M abscessus subsp abscessus	689	GCA_900141115	Respiratory system	Positive	Positive	negative	negative	negative	negative
#N/A	882	Mbaa0443	M abscessus subsp abscessus	690	GCA_900141145	Respiratory system	Positive	Positive	negative	negative	negative	negative
#N/A	752	Mbaa0444	M abscessus subsp abscessus	691	GCA_900141155	Respiratory system	Positive	Positive	negative	negative	negative	negative
#N/A	651	Mbaa0445	M abscessus subsp abscessus	692	GCA_900139215	Respiratory system	Positive	Positive	negative	negative	negative	negative
#N/A	202	Mbaa0446	M abscessus subsp abscessus	693	GCA_900141165	Respiratory system	Positive	Positive	negative	negative	negative	negative
#N/A	270	Mbaa0447	M abscessus subsp abscessus	696	GCA_900141205	Respiratory system	Positive	Positive	negative	negative	negative	negative
#N/A	672	Mbaa0448	M abscessus subsp abscessus	697	GCA_900139235	Respiratory system	Positive	Positive	negative	negative	negative	negative
#N/A	396	Mbaa0449	M abscessus subsp abscessus	698	GCA_900141255	Respiratory system	Positive	Positive	negative	negative	negative	negative
#N/A	395	Mbaa0450	M abscessus subsp abscessus	699	GCA_900139245	Respiratory system	Positive	Positive	negative	negative	negative	negative
#N/A	634	Mbaa0451	M abscessus subsp abscessus	700	GCA_900141215	Respiratory system	Positive	Positive	negative	negative	negative	negative
#N/A	59	Mbaa0452	M abscessus subsp abscessus	701	GCA_900141265	Respiratory system	Positive	Positive	negative	negative	negative	negative
#N/A	267	Mbaa0453	M abscessus subsp abscessus	702	GCA_900141235	Respiratory system	Positive	Positive	negative	negative	negative	negative
#N/A	645	Mbaa0456	M abscessus subsp abscessus	708	GCA_900141305	Respiratory system	Positive	Positive	negative	negative	negative	negative
#N/A	63	Mbaa0457	M abscessus subsp abscessus	709	GCA_900139285	Respiratory system	Positive	Positive	negative	negative	negative	negative
#N/A	126	Mbaa0458	M abscessus subsp abscessus	710	GCA_900140025	Respiratory system	Positive	Positive	negative	negative	negative	negative
#N/A	52	Mbaa0459	M abscessus subsp abscessus	711	GCA_900141325	Respiratory system	Positive	Positive	negative	negative	negative	negative
#N/A	394	Mbaa0460	M abscessus subsp abscessus	712	GCA_900141335	Respiratory system	Positive	Positive	negative	negative	negative	negative
#N/A	747	Mbaa0461	M abscessus subsp abscessus	713	GCA_900141295	Respiratory system	Positive	Positive	negative	negative	negative	negative
#N/A	646	Mbaa0462	M abscessus subsp abscessus	714	GCA_900140035	Respiratory system	Positive	Positive	negative	negative	negative	negative
#N/A	649	Mbaa0463	M abscessus subsp abscessus	715	GCA_900139855	Respiratory system	Positive	Positive	negative	negative	negative	negative
#N/A	204	Mbaa0464	M abscessus subsp abscessus	716	GCA_900139845	Respiratory system	Positive	Positive	negative	negative	negative	negative
#N/A	268	Mbaa0465	M abscessus subsp abscessus	717	GCA_900141345	Respiratory system	Positive	Positive	negative	negative	negative	negative
#N/A	58	Mbaa0466	M abscessus subsp abscessus	718	GCA_900139725	Respiratory system	Positive	Positive	negative	negative	negative	negative
#N/A	883	Mbaa0467	M abscessus subsp abscessus	719	GCA_900139265	Respiratory system	Positive	Positive	negative	negative	negative	negative
#N/A	250	Mbaa0468	M abscessus subsp abscessus	720	GCA_900139745	Respiratory system	Positive	Positive	negative	negative	negative	negative
#N/A	92	Mbaa0469	M abscessus subsp abscessus	721	GCA_900139275	Respiratory system	Positive	Positive	negative	negative	negative	negative
#N/A	388	Mbaa0471	M abscessus subsp abscessus	723	GCA_900139865	Respiratory system	Positive	Positive	negative	negative	negative	negative
#N/A	389	Mbaa0472	M abscessus subsp abscessus	724	GCA_900141315	Respiratory system	Positive	Positive	negative	negative	negative	negative

#N/A	269	Mbaa0473	M abscessus subsp abscessus	725	GCA_900139305	Respiratory system		Positive	Positive	negative	negative	negative	negative
#N/A	229	Mbaa0474	M abscessus subsp abscessus	741	GCA_900130965	Respiratory system		Positive	Positive	negative	negative	negative	negative
#N/A	224	Mbaa0475	M abscessus subsp abscessus	742	GCA_900131435	Respiratory system		Positive	Positive	negative	negative	negative	negative
#N/A	226	Mbaa0476	M abscessus subsp abscessus	743	GCA_900130985	Respiratory system		Positive	Positive	negative	negative	negative	negative
#N/A	103	Mbaa0477	M abscessus subsp abscessus	744	GCA_900134195	Respiratory system		Positive	Positive	negative	negative	negative	negative
#N/A	97	Mbaa0478	M abscessus subsp abscessus	745	GCA_900135815	Respiratory system		Positive	Positive	negative	negative	negative	negative
#N/A	100	Mbaa0479	M abscessus subsp abscessus	746	GCA_900136935	Respiratory system		Positive	Positive	negative	negative	negative	negative
#N/A	99	Mbaa0480	M abscessus subsp abscessus	747	GCA_900136915	Respiratory system		Positive	Positive	negative	negative	negative	negative
#N/A	639	Mbaa0481	M abscessus subsp abscessus	756	GCA_900135655	Respiratory system		Positive	Positive	negative	negative	negative	negative
#N/A	939	Mbaa0482	M abscessus subsp abscessus	757	GCA_900130565	Respiratory system		Positive	Positive	negative	negative	negative	negative
#N/A	633	Mbaa0483	M abscessus subsp abscessus	758	GCA_900135665	Respiratory system		Positive	Positive	negative	negative	negative	negative
#N/A	1001	Mbaa0484	M abscessus subsp abscessus	759	GCA_900135675	Respiratory system		Positive	Positive	negative	negative	negative	negative
#N/A	629	Mbaa0485	M abscessus subsp abscessus	760	GCA_900131445	Respiratory system		Positive	Positive	negative	negative	negative	negative
#N/A	624	Mbaa0486	M abscessus subsp abscessus	761	GCA_900131475	Respiratory system		Positive	Positive	negative	negative	negative	negative
#N/A	627	Mbaa0487	M abscessus subsp abscessus	762	GCA_900131805	Respiratory system		Positive	Positive	negative	negative	negative	negative
#N/A	623	Mbaa0488	M abscessus subsp abscessus	763	GCA_900131825	Respiratory system		Positive	Positive	negative	negative	negative	negative
#N/A	625	Mbaa0489	M abscessus subsp abscessus	764	GCA_900132515	Respiratory system		Positive	Positive	negative	negative	negative	negative
#N/A	626	Mbaa0490	M abscessus subsp abscessus	765	GCA_900133545	Respiratory system		Positive	Positive	negative	negative	negative	negative
#N/A	621	Mbaa0491	M abscessus subsp abscessus	766	GCA_900134185	Respiratory system		Positive	Positive	negative	negative	negative	negative
#N/A	628	Mbaa0492	M abscessus subsp abscessus	767	GCA_900135685	Respiratory system		Positive	Positive	negative	negative	negative	negative
#N/A	622	Mbaa0493	M abscessus subsp abscessus	768	GCA_900136955	Respiratory system		Positive	Positive	negative	negative	negative	negative
#N/A	5	Mbaa0494	M abscessus subsp abscessus	769	GCA_900130235	Respiratory system		Positive	Positive	negative	negative	negative	negative
#N/A	183	Mbaa0495	M abscessus subsp abscessus	770	GCA_900130685	Respiratory system		Positive	Positive	negative	negative	negative	negative
#N/A	181	Mbaa0496	M abscessus subsp abscessus	771	GCA_900130695	Respiratory system		Positive	Positive	negative	negative	negative	negative
#N/A	180	Mbaa0497	M abscessus subsp abscessus	772	GCA_900131465	Respiratory system		Positive	Positive	negative	negative	negative	negative
#N/A	182	Mbaa0498	M abscessus subsp abscessus	773	GCA_900132175	Respiratory system		Positive	Positive	negative	negative	negative	negative
#N/A	6	Mbaa0499	M abscessus subsp abscessus	774	GCA_900130705	Respiratory system		Positive	Positive	negative	negative	negative	negative
#N/A	654	Mbaa0500	M abscessus subsp abscessus	775	GCA_900133455	Respiratory system		Positive	Positive	negative	negative	negative	negative
#N/A	228	Mbaa0501	M abscessus subsp abscessus	776	GCA_900131785	Respiratory system		Positive	Positive	negative	negative	negative	negative
#N/A	227	Mbaa0502	M abscessus subsp abscessus	777	GCA_900132205	Respiratory system		Positive	Positive	negative	negative	negative	negative
#N/A	282	Mbaa0503	M abscessus subsp abscessus	778	GCA_900133515	Respiratory system		Positive	Positive	negative	negative	negative	negative
#N/A	281	Mbaa0504	M abscessus subsp abscessus	779	GCA_900134155	Respiratory system		Positive	Positive	negative	negative	negative	negative
#N/A	852	Mbaa0505	M abscessus subsp abscessus	780	GCA_900134175	Respiratory system		Positive	Positive	negative	negative	negative	negative
#N/A	851	Mbaa0506	M abscessus subsp abscessus	781	GCA_900134205	Respiratory system		Positive	Positive	negative	negative	negative	negative
#N/A	850	Mbaa0507	M abscessus subsp abscessus	782	GCA_900135845	Respiratory system		Positive	Positive	negative	negative	negative	negative
#N/A	849	Mbaa0508	M abscessus subsp abscessus	783	GCA_900137085	Respiratory system		Positive	Positive	negative	negative	negative	negative
#N/A	260	Mbaa0509	M abscessus subsp abscessus	793	GCA_900131005	Respiratory system		Positive	Positive	negative	negative	negative	negative
#N/A	178	Mbaa0510	M abscessus subsp abscessus	801	GCA_900130725	Respiratory system		Positive	Positive	negative	negative	negative	negative
#N/A	179	Mbaa0511	M abscessus subsp abscessus	802	GCA_900131015	Respiratory system		Positive	Positive	negative	negative	negative	negative
#N/A	810	Mbaa0512	M abscessus subsp abscessus	803	GCA_900133495	Respiratory system		Positive	Positive	negative	negative	negative	negative
#N/A	304	Mbaa0513	M abscessus subsp abscessus	804	GCA_900135705	Respiratory system		Positive	Positive	negative	negative	negative	negative
#N/A	301	Mbaa0514	M abscessus subsp abscessus	806	GCA_900136975	Respiratory system		Positive	Positive	negative	negative	negative	negative
#N/A	298	Mbaa0515	M abscessus subsp abscessus	807	GCA_900135725	Respiratory system		Positive	Positive	negative	negative	negative	negative
#N/A	299	Mbaa0516	M abscessus subsp abscessus	808	GCA_900135765	Respiratory system		Positive	Positive	negative	negative	negative	negative
#N/A	300	Mbaa0517	M abscessus subsp abscessus	809	GCA_900136985	Respiratory system		Positive	Positive	negative	negative	negative	negative
#N/A	302	Mbaa0518	M abscessus subsp abscessus	810	GCA_900136995	Respiratory system		Positive	Positive	negative	negative	negative	negative
#N/A	12	Mbaa0519	M abscessus subsp abscessus	811	GCA_900137005	Respiratory system		Positive	Positive	negative	negative	negative	negative
#N/A	101	Mbaa0520	M abscessus subsp abscessus	814	GCA_900134225	Respiratory system		Positive	Positive	negative	negative	negative	negative
#N/A	102	Mbaa0521	M abscessus subsp abscessus	815	GCA_900135775	Respiratory system		Positive	Positive	negative	negative	negative	negative
#N/A	295	Mbaa0522	M abscessus subsp abscessus	816	GCA_900132485	Respiratory system		Positive	Positive	negative	negative	negative	negative
#N/A	618	Mbaa0523	M abscessus subsp abscessus	817	GCA_900132525	Respiratory system		Positive	Positive	negative	negative	negative	negative
#N/A	617	Mbaa0524	M abscessus subsp abscessus	818	GCA_900132935	Respiratory system		Positive	Positive	negative	negative	negative	negative
#N/A	614	Mbaa0525	M abscessus subsp abscessus	819	GCA_900134255	Respiratory system		Positive	Positive	negative	negative	negative	negative

#N/A	616	Mbaa0526	M abscessus subsp abscessus	820	GCA_900135785	Respiratory system	Positive	Positive	negative	negative	negative	negative
#N/A	615	Mbaa0527	M abscessus subsp abscessus	821	GCA_900137045	Respiratory system	Positive	Positive	negative	negative	negative	negative
#N/A	613	Mbaa0528	M abscessus subsp abscessus	822	GCA_900137065	Respiratory system	Positive	Positive	negative	negative	negative	negative
#N/A	104	Mbaa0529	M abscessus subsp abscessus	823	GCA_900132875	Respiratory system	Positive	Positive	negative	negative	negative	negative
#N/A	105	Mbaa0530	M abscessus subsp abscessus	824	GCA_900133525	Respiratory system	Positive	Positive	negative	negative	negative	negative
#N/A	927	Mbaa0531	M abscessus subsp abscessus	827	GCA_900137015	Respiratory system	Positive	Positive	negative	negative	negative	negative
#N/A	926	Mbaa0532	M abscessus subsp abscessus	828	GCA_900132905	Respiratory system	Positive	Positive	negative	negative	negative	negative
#N/A	925	Mbaa0533	M abscessus subsp abscessus	829	GCA_900133535	Respiratory system	Positive	Positive	negative	negative	negative	negative
#N/A	920	Mbaa0534	M abscessus subsp abscessus	830	GCA_900134215	Respiratory system	Positive	Positive	negative	negative	negative	negative
#N/A	918	Mbaa0535	M abscessus subsp abscessus	831	GCA_900134245	Respiratory system	Positive	Positive	negative	negative	negative	negative
#N/A	921	Mbaa0536	M abscessus subsp abscessus	832	GCA_900135795	Respiratory system	Positive	Positive	negative	negative	negative	negative
#N/A	924	Mbaa0537	M abscessus subsp abscessus	833	GCA_900137035	Respiratory system	Positive	Positive	negative	negative	negative	negative
#N/A	922	Mbaa0538	M abscessus subsp abscessus	834	GCA_900137075	Respiratory system	Positive	Positive	negative	negative	negative	negative
#N/A	923	Mbaa0539	M abscessus subsp abscessus	835	GCA_900138025	Respiratory system	Positive	Positive	negative	negative	negative	negative
#N/A	2	Mbaa0540	M abscessus subsp abscessus	836	GCA_900137115	Respiratory system	Positive	Positive	negative	negative	negative	negative
#N/A	934	Mbaa0541	M abscessus subsp abscessus	841	GCA_900131485	Respiratory system	Positive	Positive	negative	negative	negative	negative
#N/A	9	Mbaa0542	M abscessus subsp abscessus	844	GCA_900137995	Respiratory system	Positive	Positive	negative	negative	negative	negative
#N/A	234	Mbaa0543	M abscessus subsp abscessus	851	GCA_900132995	Respiratory system	Positive	Positive	negative	negative	negative	negative
#N/A	232	Mbaa0544	M abscessus subsp abscessus	852	GCA_900132915	Respiratory system	Positive	Positive	negative	negative	negative	negative
#N/A	230	Mbaa0545	M abscessus subsp abscessus	853	GCA_900133555	Respiratory system	Positive	Positive	negative	negative	negative	negative
#N/A	231	Mbaa0546	M abscessus subsp abscessus	854	GCA_900133565	Respiratory system	Positive	Positive	negative	negative	negative	negative
#N/A	233	Mbaa0547	M abscessus subsp abscessus	855	GCA_900135805	Respiratory system	Positive	Positive	negative	negative	negative	negative
#N/A	44	Mbaa0548	M abscessus subsp abscessus	857	GCA_900135855	Respiratory system	Positive	Positive	negative	negative	negative	negative
#N/A	43	Mbaa0549	M abscessus subsp abscessus	858	GCA_900135825	Respiratory system	Positive	Positive	negative	negative	negative	negative
#N/A	1002	Mbaa0550	M abscessus subsp abscessus	859	GCA_900137055	Respiratory system	Positive	Positive	negative	negative	negative	negative
#N/A	491	Mbaa0551	M abscessus subsp abscessus	876	GCA_900134265	Respiratory system	Positive	Positive	negative	negative	negative	negative
#N/A	488	Mbaa0552	M abscessus subsp abscessus	877	GCA_900134305	Respiratory system	Positive	Positive	negative	negative	negative	negative
#N/A	489	Mbaa0553	M abscessus subsp abscessus	878	GCA_900135935	Respiratory system	Positive	Positive	negative	negative	negative	negative
#N/A	619	Mbaa0554	M abscessus subsp abscessus	880	GCA_900132555	Respiratory system	Positive	Positive	negative	negative	negative	negative
#N/A	620	Mbaa0555	M abscessus subsp abscessus	881	GCA_900132545	Respiratory system	Positive	Positive	negative	negative	negative	negative
#N/A	490	Mbaa0556	M abscessus subsp abscessus	887	GCA_900132255	Respiratory system	Positive	Positive	negative	negative	negative	negative
#N/A	116	Mbaa0557	M abscessus subsp abscessus	888	GCA_900132605	Respiratory system	Positive	Positive	negative	negative	negative	negative
#N/A	938	Mbaa0558	M abscessus subsp abscessus	889	GCA_900131045	Respiratory system	Positive	Positive	negative	negative	negative	negative
#N/A	935	Mbaa0559	M abscessus subsp abscessus	890	GCA_900131495	Respiratory system	Positive	Positive	negative	negative	negative	negative
#N/A	937	Mbaa0560	M abscessus subsp abscessus	891	GCA_900131845	Respiratory system	Positive	Positive	negative	negative	negative	negative
#N/A	559	Mbaa0561	M abscessus subsp abscessus	892	GCA_900141385	Respiratory system	Positive	Positive	negative	negative	negative	negative
#N/A	795	Mbaa0562	M abscessus subsp abscessus	894	GCA_900141365	Respiratory system	Positive	Positive	negative	negative	negative	negative
#N/A	578	Mbaa0563	M abscessus subsp abscessus	895	GCA_900141445	Respiratory system	Positive	Positive	negative	negative	negative	negative
#N/A	220	Mbaa0564	M abscessus subsp abscessus	896	GCA_900141435	Respiratory system	Positive	Positive	negative	negative	negative	negative
#N/A	507	Mbaa0565	M abscessus subsp abscessus	897	GCA_900141375	Respiratory system	Positive	Positive	Positive	negative	negative	negative
#N/A	11	Mbaa0566	M abscessus subsp abscessus	898	GCA_900141395	Respiratory system	Positive	Positive	negative	negative	negative	negative
#N/A	437	Mbaa0567	M abscessus subsp abscessus	900	GCA_900141405	Respiratory system	Positive	Positive	negative	negative	negative	negative
#N/A	194	Mbaa0568	M abscessus subsp abscessus	901	GCA_900141455	Respiratory system	Positive	Positive	negative	negative	negative	negative
#N/A	431	Mbaa0569	M abscessus subsp abscessus	904	GCA_900138065	Respiratory system	Positive	Positive	negative	negative	negative	negative
#N/A	691	Mbaa0570	M abscessus subsp abscessus	907	GCA_900133005	Respiratory system	Positive	Positive	negative	negative	negative	negative
#N/A	469	Mbaa0571	M abscessus subsp abscessus	908	GCA_900133035	Respiratory system	Positive	Positive	negative	negative	negative	negative
#N/A	27	Mbaa0572	M abscessus subsp abscessus	910	GCA_900133075	Respiratory system	Positive	Positive	negative	negative	negative	negative
#N/A	29	Mbaa0573	M abscessus subsp abscessus	913	GCA_900132945	Respiratory system	Positive	Positive	negative	negative	negative	negative
#N/A	156	Mbaa0574	M abscessus subsp abscessus	914	GCA_900132955	Respiratory system	Positive	Positive	negative	negative	negative	negative
#N/A	21	Mbaa0575	M abscessus subsp abscessus	915	GCA_900132965	Respiratory system	Positive	Positive	negative	negative	negative	negative
#N/A	834	Mbaa0576	M abscessus subsp abscessus	916	GCA_900133095	Respiratory system	Positive	Positive	negative	negative	negative	negative
#N/A	738	Mbaa0577	M abscessus subsp abscessus	917	GCA_900133085	Respiratory system	Positive	Positive	negative	negative	negative	negative
#N/A	38	Mbaa0578	M abscessus subsp abscessus	918	GCA_900133115	Respiratory system	Positive	Positive	negative	negative	negative	negative

#N/A	604	Mbaa0579	M abscessus subsp abscessus	921	GCA_900133125	Respiratory system		Positive	Positive	negative	negative	negative	negative
#N/A	832	Mbaa0580	M abscessus subsp abscessus	929	GCA_900133815	Respiratory system		Positive	Positive	negative	negative	negative	negative
#N/A	39	Mbaa0581	M abscessus subsp abscessus	930	GCA_900133835	Respiratory system		Positive	Positive	negative	negative	negative	negative
#N/A	861	Mbaa0582	M abscessus subsp abscessus	931	GCA_900133655	Respiratory system		Positive	Positive	negative	negative	negative	negative
#N/A	692	Mbaa0583	M abscessus subsp abscessus	935	GCA_900133665	Respiratory system		Positive	Positive	negative	negative	negative	negative
#N/A	858	Mbaa0584	M abscessus subsp abscessus	936	GCA_900133645	Respiratory system		Positive	Positive	negative	negative	negative	negative
#N/A	136	Mbaa0585	M abscessus subsp abscessus	937	GCA_900133685	Respiratory system		Positive	Positive	negative	negative	negative	negative
#N/A	828	Mbaa0586	M abscessus subsp abscessus	941	GCA_900133715	Respiratory system		Positive	Positive	negative	negative	negative	negative
#N/A	707	Mbaa0587	M abscessus subsp abscessus	942	GCA_900133705	Respiratory system		Positive	Positive	negative	negative	negative	negative
#N/A	854	Mbaa0588	M abscessus subsp abscessus	944	GCA_900133775	Respiratory system		Positive	Positive	negative	negative	negative	negative
#N/A	137	Mbaa0589	M abscessus subsp abscessus	949	GCA_900133575	Respiratory system		Positive	Positive	negative	negative	negative	negative
#N/A	855	Mbaa0590	M abscessus subsp abscessus	951	GCA_900133805	Respiratory system		Positive	Positive	negative	negative	negative	negative
#N/A	353	Mbaa0591	M abscessus subsp abscessus	952	GCA_900134395	Respiratory system		Positive	Positive	negative	negative	negative	negative
#N/A	94	Mbaa0592	M abscessus subsp abscessus	955	GCA_900134455	Respiratory system		Positive	Positive	negative	negative	negative	negative
#N/A	708	Mbaa0593	M abscessus subsp abscessus	956	GCA_900134435	Respiratory system		Positive	Positive	negative	negative	negative	negative
#N/A	833	Mbaa0594	M abscessus subsp abscessus	961	GCA_900134465	Respiratory system		Positive	Positive	negative	negative	negative	negative
#N/A	150	Mbaa0595	M abscessus subsp abscessus	962	GCA_900134485	Respiratory system		Positive	Positive	negative	negative	negative	negative
#N/A	706	Mbaa0596	M abscessus subsp abscessus	963	GCA_900134505	Respiratory system		Positive	Positive	negative	negative	negative	negative
#N/A	17	Mbaa0597	M abscessus subsp abscessus	964	GCA_900134515	Respiratory system		Positive	Positive	negative	negative	negative	negative
#N/A	857	Mbaa0598	M abscessus subsp abscessus	965	GCA_900134525	Respiratory system		Positive	Positive	negative	negative	negative	negative
#N/A	138	Mbaa0599	M abscessus subsp abscessus	966	GCA_900134345	Respiratory system		Positive	Positive	negative	negative	negative	negative
#N/A	856	Mbaa0600	M abscessus subsp abscessus	970	GCA_900134585	Respiratory system		Positive	Positive	negative	negative	negative	negative
#N/A	603	Mbaa0601	M abscessus subsp abscessus	972	GCA_900134355	Respiratory system		Positive	Positive	negative	negative	negative	negative
#N/A	147	Mbaa0602	M abscessus subsp abscessus	973	GCA_900134555	Respiratory system		Positive	Positive	negative	negative	negative	negative
#N/A	809	Mbaa0603	M abscessus subsp abscessus	975	GCA_900134615	Respiratory system		Positive	Positive	negative	negative	negative	negative
#N/A	579	Mbaa0604	M abscessus subsp abscessus	976	GCA_900134595	Respiratory system		Positive	Positive	negative	negative	negative	negative
#N/A	305	Mbaa0605	M abscessus subsp abscessus	977	GCA_900134625	Respiratory system		Positive	Positive	negative	negative	negative	negative
#N/A	682	Mbaa0606	M abscessus subsp abscessus	978	GCA_900134605	Respiratory system		Positive	Positive	negative	negative	negative	negative
#N/A	774	Mbaa0607	M abscessus subsp abscessus	980	GCA_900135975	Respiratory system		Positive	Positive	negative	negative	negative	negative
#N/A	806	Mbaa0608	M abscessus subsp abscessus	982	GCA_900136015	Respiratory system		Positive	Positive	negative	negative	negative	negative
#N/A	679	Mbaa0609	M abscessus subsp abscessus	984	GCA_900136035	Respiratory system		Positive	Positive	negative	negative	negative	negative
#N/A	151	Mbaa0610	M abscessus subsp abscessus	985	GCA_900136055	Respiratory system		Positive	Positive	negative	negative	negative	negative
#N/A	664	Mbaa0611	M abscessus subsp abscessus	986	GCA_900136045	Respiratory system		Positive	Positive	negative	negative	negative	negative
#N/A	23	Mbaa0612	M abscessus subsp abscessus	987	GCA_900136105	Respiratory system		Positive	Positive	negative	negative	negative	negative
#N/A	139	Mbaa0613	M abscessus subsp abscessus	988	GCA_900136065	Respiratory system		Positive	Positive	negative	negative	negative	negative
#N/A	146	Mbaa0614	M abscessus subsp abscessus	989	GCA_900136075	Respiratory system		Positive	Positive	negative	negative	negative	negative
#N/A	140	Mbaa0615	M abscessus subsp abscessus	990	GCA_900135945	Respiratory system		Positive	Positive	negative	negative	negative	negative
#N/A	174	Mbaa0616	M abscessus subsp abscessus	991	GCA_900136185	Respiratory system		Positive	Positive	negative	negative	negative	negative
#N/A	84	Mbaa0617	M abscessus subsp abscessus	992	GCA_900136095	Respiratory system		Positive	Positive	negative	negative	negative	negative
#N/A	162	Mbaa0618	M abscessus subsp abscessus	993	GCA_900136155	Respiratory system		Positive	Positive	negative	negative	negative	negative
#N/A	175	Mbaa0619	M abscessus subsp abscessus	994	GCA_900136085	Respiratory system		Positive	Positive	negative	negative	negative	negative
#N/A	163	Mbaa0620	M abscessus subsp abscessus	996	GCA_900136115	Respiratory system		Positive	Positive	negative	negative	negative	negative
#N/A	676	Mbaa0621	M abscessus subsp abscessus	997	GCA_900136135	Respiratory system		Positive	Positive	negative	negative	negative	negative
#N/A	152	Mbaa0622	M abscessus subsp abscessus	998	GCA_900136145	Respiratory system		Positive	Positive	negative	negative	negative	negative
#N/A	407	Mbaa0623	M abscessus subsp abscessus	1002	GCA_900136215	Respiratory system		Positive	Positive	negative	negative	negative	negative
#N/A	349	Mbaa0624	M abscessus subsp abscessus	1003	GCA_900135925	Respiratory system		Positive	Positive	negative	negative	negative	negative
#N/A	173	Mbaa0625	M abscessus subsp abscessus	1009	GCA_900136245	Respiratory system		Positive	Positive	negative	negative	negative	negative
#N/A	677	Mbaa0626	M abscessus subsp abscessus	1010	GCA_900136255	Respiratory system		Positive	Positive	negative	negative	negative	negative
#N/A	588	Mbaa0627	M abscessus subsp abscessus	1011	GCA_900136265	Respiratory system		Positive	Positive	negative	negative	negative	negative
#N/A	22	Mbaa0628	M abscessus subsp abscessus	1013	GCA_900136285	Respiratory system		Positive	Positive	negative	negative	negative	negative
#N/A	666	Mbaa0629	M abscessus subsp abscessus	1016	GCA_900136305	Respiratory system		Positive	Positive	negative	negative	negative	negative
#N/A	164	Mbaa0630	M abscessus subsp abscessus	1019	GCA_900136355	Respiratory system		Positive	Positive	negative	negative	negative	negative
#N/A	359	Mbaa0631	M abscessus subsp abscessus	1020	GCA_900137255	Respiratory system		Positive	Positive	negative	negative	negative	negative

#N/A	285	Mbaa0632	M abscessus subsp abscessus	1023	GCA_900137285	Respiratory system	Positive	Positive	negative	negative	negative	negative
#N/A	165	Mbaa0633	M abscessus subsp abscessus	1024	GCA_900137275	Respiratory system	Positive	Positive	negative	negative	negative	negative
#N/A	589	Mbaa0634	M abscessus subsp abscessus	1025	GCA_900137315	Respiratory system	Positive	Positive	negative	negative	negative	negative
#N/A	171	Mbaa0635	M abscessus subsp abscessus	1026	GCA_900137305	Respiratory system	Positive	Positive	negative	negative	negative	negative
#N/A	608	Mbaa0636	M abscessus subsp abscessus	1027	GCA_900137325	Respiratory system	Positive	Positive	negative	negative	negative	negative
#N/A	610	Mbaa0637	M abscessus subsp abscessus	1028	GCA_900137455	Respiratory system	Positive	Positive	negative	negative	negative	negative
#N/A	286	Mbaa0638	M abscessus subsp abscessus	1029	GCA_900137205	Respiratory system	Positive	Positive	negative	negative	negative	negative
#N/A	352	Mbaa0639	M abscessus subsp abscessus	1030	GCA_900137545	Respiratory system	Positive	Positive	negative	negative	negative	negative
#N/A	357	Mbaa0640	M abscessus subsp abscessus	1031	GCA_900137645	Respiratory system	Positive	Positive	negative	negative	negative	negative
#N/A	289	Mbaa0641	M abscessus subsp abscessus	1034	GCA_900137865	Respiratory system	Positive	Positive	negative	negative	negative	negative
#N/A	18	Mbaa0642	M abscessus subsp abscessus	1036	GCA_900137345	Respiratory system	Positive	Positive	negative	negative	negative	negative
#N/A	354	Mbaa0643	M abscessus subsp abscessus	1037	GCA_900137365	Respiratory system	Positive	Positive	negative	negative	negative	negative
#N/A	429	Mbaa0644	M abscessus subsp abscessus	1039	GCA_900137375	Respiratory system	Positive	Positive	negative	negative	negative	negative
#N/A	428	Mbaa0645	M abscessus subsp abscessus	1040	GCA_900137355	Respiratory system	Positive	Positive	negative	negative	negative	negative
#N/A	20	Mbaa0646	M abscessus subsp abscessus	1041	GCA_900137385	Respiratory system	Positive	Positive	negative	negative	negative	negative
#N/A	845	Mbaa0647	M abscessus subsp abscessus	1042	GCA_900137395	Respiratory system	Positive	Positive	negative	negative	negative	negative
#N/A	141	Mbaa0648	M abscessus subsp abscessus	1043	GCA_900137405	Respiratory system	Positive	Positive	negative	negative	negative	negative
#N/A	153	Mbaa0649	M abscessus subsp abscessus	1044	GCA_900137415	Respiratory system	Positive	Positive	negative	negative	negative	negative
#N/A	837	Mbaa0650	M abscessus subsp abscessus	1045	GCA_900137435	Respiratory system	Positive	Positive	negative	negative	negative	negative
#N/A	256	Mbaa0651	M abscessus subsp abscessus	1046	GCA_900137425	Respiratory system	Positive	Positive	negative	negative	negative	negative
#N/A	410	Mbaa0652	M abscessus subsp abscessus	1047	GCA_900137445	Respiratory system	Positive	Positive	negative	negative	negative	negative
#N/A	596	Mbaa0653	M abscessus subsp abscessus	1050	GCA_900137465	Respiratory system	Positive	Positive	negative	negative	negative	negative
#N/A	142	Mbaa0654	M abscessus subsp abscessus	1051	GCA_900137495	Respiratory system	Positive	Positive	negative	negative	negative	negative
#N/A	829	Mbaa0655	M abscessus subsp abscessus	1053	GCA_900137505	Respiratory system	Positive	Positive	negative	negative	negative	negative
#N/A	782	Mbaa0656	M abscessus subsp abscessus	1054	GCA_900137525	Respiratory system	Positive	Positive	negative	negative	negative	negative
#N/A	408	Mbaa0657	M abscessus subsp abscessus	1055	GCA_900137515	Respiratory system	Positive	Positive	negative	negative	negative	negative
#N/A	335	Mbaa0658	M abscessus subsp abscessus	1058	GCA_900137595	Respiratory system	Positive	Positive	negative	negative	negative	negative
#N/A	597	Mbaa0659	M abscessus subsp abscessus	1059	GCA_900137565	Respiratory system	Positive	Positive	negative	negative	negative	negative
#N/A	430	Mbaa0660	M abscessus subsp abscessus	1060	GCA_900137585	Respiratory system	Positive	Positive	negative	negative	negative	negative
#N/A	19	Mbaa0661	M abscessus subsp abscessus	1061	GCA_900137575	Respiratory system	Positive	Positive	negative	negative	negative	negative
#N/A	779	Mbaa0662	M abscessus subsp abscessus	1062	GCA_900137605	Respiratory system	Positive	Positive	negative	negative	negative	negative
#N/A	154	Mbaa0663	M abscessus subsp abscessus	1063	GCA_900137235	Respiratory system	Positive	Positive	negative	negative	negative	negative
#N/A	333	Mbaa0664	M abscessus subsp abscessus	1065	GCA_900137665	Respiratory system	Positive	Positive	negative	negative	negative	negative
#N/A	24	Mbaa0665	M abscessus subsp abscessus	1067	GCA_900137675	Respiratory system	Positive	Positive	negative	negative	negative	negative
#N/A	680	Mbaa0666	M abscessus subsp abscessus	1068	GCA_900137615	Respiratory system	negative	Positive	negative	negative	negative	negative
#N/A	26	Mbaa0667	M abscessus subsp abscessus	1069	GCA_900137655	Respiratory system	Positive	Positive	negative	negative	negative	negative
#N/A	590	Mbaa0668	M abscessus subsp abscessus	1070	GCA_900137225	Respiratory system	Positive	Positive	negative	negative	negative	negative
#N/A	780	Mbaa0669	M abscessus subsp abscessus	1071	GCA_900137685	Respiratory system	Positive	Positive	negative	negative	negative	negative
#N/A	72	Mbaa0670	M abscessus subsp abscessus	1074	GCA_900137715	Respiratory system	Positive	Positive	negative	negative	negative	negative
#N/A	334	Mbaa0671	M abscessus subsp abscessus	1075	GCA_900137765	Respiratory system	Positive	Positive	negative	negative	negative	negative
#N/A	665	Mbaa0672	M abscessus subsp abscessus	1076	GCA_900137755	Respiratory system	Positive	Positive	negative	negative	negative	negative
#N/A	690	Mbaa0673	M abscessus subsp abscessus	1077	GCA_900137735	Respiratory system	Positive	Positive	negative	negative	negative	negative
#N/A	144	Mbaa0674	M abscessus subsp abscessus	1078	GCA_900137745	Respiratory system	Positive	Positive	negative	negative	negative	negative
#N/A	264	Mbaa0675	M abscessus subsp abscessus	1080	GCA_900137795	Respiratory system	Positive	Positive	negative	negative	negative	negative
#N/A	257	Mbaa0676	M abscessus subsp abscessus	1081	GCA_900137125	Respiratory system	Positive	Positive	negative	negative	negative	negative
#N/A	143	Mbaa0677	M abscessus subsp abscessus	1082	GCA_900137805	Respiratory system	Positive	Positive	negative	negative	negative	negative
#N/A	789	Mbaa0678	M abscessus subsp abscessus	1083	GCA_900137785	Respiratory system	Positive	Positive	negative	negative	negative	negative
#N/A	580	Mbaa0679	M abscessus subsp abscessus	1084	GCA_900137815	Respiratory system	Positive	Positive	negative	negative	negative	negative
#N/A	744	Mbaa0680	M abscessus subsp abscessus	1085	GCA_900137825	Respiratory system	Positive	Positive	negative	negative	negative	negative
#N/A	541	Mbaa0681	M abscessus subsp abscessus	1086	GCA_900138285	Respiratory system	Positive	Positive	negative	negative	negative	negative
#N/A	936	Mbaa0682	M abscessus subsp abscessus	1087	GCA_900132625	Respiratory system	Positive	Positive	negative	negative	negative	negative
#N/A	16	Mbaa0683	M abscessus subsp abscessus	1088	GCA_900133175	Respiratory system	Positive	Positive	negative	negative	negative	negative
#N/A	14	Mbaa0684	M abscessus subsp abscessus	1089	GCA_900134365	Respiratory system	Positive	Positive	negative	negative	negative	negative

#N/A	15	Mbaa0685	M abscessus subsp abscessus	1090	GCA_900134645	Respiratory system		Positive	Positive	negative	negative	negative	negative
#N/A	188	Mbaa0686	M abscessus subsp abscessus	1095	GCA_900134665	Respiratory system		Positive	Positive	negative	negative	negative	negative
#N/A	480	Mbaa0687	M abscessus subsp abscessus	1096	GCA_900137845	Respiratory system		Positive	Positive	negative	negative	negative	negative
#N/A	472	Mbaa0688	M abscessus subsp abscessus	1097	GCA_900138275	Respiratory system		Positive	Positive	negative	negative	negative	negative
#N/A	478	Mbaa0689	M abscessus subsp abscessus	1098	GCA_900141565	Respiratory system		Positive	Positive	negative	negative	negative	negative
#N/A	475	Mbaa0690	M abscessus subsp abscessus	1099	GCA_900141535	Respiratory system		Positive	Positive	negative	negative	negative	negative
#N/A	476	Mbaa0691	M abscessus subsp abscessus	1100	GCA_900141545	Respiratory system		Positive	Positive	negative	negative	negative	negative
#N/A	471	Mbaa0692	M abscessus subsp abscessus	1101	GCA_900141485	Respiratory system		Positive	Positive	negative	negative	negative	negative
#N/A	474	Mbaa0693	M abscessus subsp abscessus	1102	GCA_900141575	Respiratory system		Positive	Positive	negative	negative	negative	negative
#N/A	473	Mbaa0694	M abscessus subsp abscessus	1103	GCA_900141585	Respiratory system		Positive	Positive	negative	negative	negative	negative
#N/A	479	Mbaa0695	M abscessus subsp abscessus	1104	GCA_900141495	Respiratory system		Positive	Positive	negative	negative	negative	negative
#N/A	477	Mbaa0696	M abscessus subsp abscessus	1105	GCA_900141615	Respiratory system		Positive	Positive	negative	negative	negative	negative
#N/A	897	Mbaa0697	M abscessus subsp abscessus	1106	GCA_900141555	Respiratory system		Positive	Positive	negative	negative	negative	negative
#N/A	225	Mbaa0698	M abscessus subsp abscessus	1108	GCA_900132295	Respiratory system		Positive	Positive	negative	negative	negative	negative
#N/A	98	Mbaa0699	M abscessus subsp abscessus	1109	GCA_900134375	Respiratory system		Positive	Positive	negative	negative	negative	negative
#N/A	641	Mbaa0700	M abscessus subsp abscessus	1112	GCA_900134385	Respiratory system		Positive	Positive	negative	negative	negative	negative
#N/A	303	Mbaa0701	M abscessus subsp abscessus	1114	GCA_900136365	Respiratory system		Positive	Positive	negative	negative	negative	negative
#N/A	481	Mbaa0702	M abscessus subsp abscessus	1116	GCA_900132575	Respiratory system		Positive	Positive	negative	negative	negative	negative
#N/A	919	Mbaa0703	M abscessus subsp abscessus	1122	GCA_900134675	Respiratory system		Positive	Positive	negative	negative	negative	negative
#N/A	258	Mbaa0704	M abscessus subsp abscessus	1123	GCA_900138145	Respiratory system		Positive	Positive	negative	negative	negative	negative
#N/A	155	Mbaa0705	M abscessus subsp abscessus	1124	GCA_900133215	Respiratory system		Positive	Positive	negative	negative	negative	negative
#N/A	586	Mbaa0706	M abscessus subsp abscessus	1125	GCA_900136385	Respiratory system		Positive	Positive	negative	negative	negative	negative
#N/A	605	Mbaa0707	M abscessus subsp abscessus	1129	GCA_900137895	Respiratory system		Positive	Positive	negative	negative	negative	negative
#N/A	25	Mbaa0708	M abscessus subsp abscessus	1131	GCA_900137975	Respiratory system		Positive	Positive	negative	negative	negative	negative
#N/A	787	Mbaa0709	M abscessus subsp abscessus	1132	GCA_900137265	Respiratory system		Positive	Positive	negative	negative	negative	negative
#N/A	788	Mbaa0710	M abscessus subsp abscessus	1133	GCA_900137915	Respiratory system		Positive	Positive	negative	negative	negative	negative
#N/A	688	Mbaa0711	M abscessus subsp abscessus	1134	GCA_900141685	Respiratory system		Positive	Positive	negative	negative	negative	negative
#N/A	34	Mbaa0712	M abscessus subsp abscessus	1136	GCA_900141525	Respiratory system		Positive	Positive	negative	negative	negative	negative
#N/A	487	Mbaa0713	M abscessus subsp abscessus	1137	GCA_900141695	Respiratory system		Positive	Positive	negative	negative	negative	negative
#N/A	773	Mbaa0714	M abscessus subsp abscessus	1138	GCA_900141645	Respiratory system		Positive	Positive	negative	negative	negative	negative
#N/A	778	Mbaa0715	M abscessus subsp abscessus	1139	GCA_900141675	Respiratory system		Positive	Positive	negative	negative	negative	negative
#N/A	243	Mbaa0716	M abscessus subsp abscessus	1142	GCA_900137215	Respiratory system		Positive	Positive	negative	negative	negative	negative
#N/A	493	Mbaa0717	M abscessus subsp abscessus	1143	GCA_900138265	Respiratory system		Positive	Positive	negative	negative	negative	negative
#N/A	297	Mbaa0718	M abscessus subsp abscessus	1144	GCA_900138045	Respiratory system		Positive	Positive	negative	negative	negative	negative
#N/A	291	Mbaa0719	M abscessus subsp abscessus	1145	GCA_900138015	Respiratory system		Positive	Positive	negative	negative	negative	negative
#N/A	288	Mbaa0720	M abscessus subsp abscessus	1146	GCA_900138175	Respiratory system		Positive	Positive	negative	negative	negative	negative
#N/A	808	Mbaa0721	M abscessus subsp abscessus	1147	GCA_900138205	Respiratory system		Positive	Positive	negative	negative	negative	negative
#N/A	771	Mbaa0722	M abscessus subsp abscessus	1148	GCA_900138185	Respiratory system		Positive	Positive	negative	negative	negative	negative
#N/A	753	Mbaa0723	M abscessus subsp abscessus	1149	GCA_900138195	Respiratory system		Positive	Positive	negative	negative	negative	negative
#N/A	287	Mbaa0724	M abscessus subsp abscessus	1150	GCA_900138035	Respiratory system		Positive	Positive	negative	negative	negative	negative
#N/A	290	Mbaa0725	M abscessus subsp abscessus	1151	GCA_900138215	Respiratory system		Positive	Positive	negative	negative	negative	negative
#N/A	769	Mbaa0726	M abscessus subsp abscessus	1152	GCA_900138255	Respiratory system		Positive	Positive	negative	negative	negative	negative
#N/A	436	Mbaa0727	M abscessus subsp abscessus	1153	GCA_900138225	Respiratory system		Positive	Positive	negative	negative	negative	negative
#N/A	777	Mbaa0728	M abscessus subsp abscessus	1154	GCA_900138095	Respiratory system		Positive	Positive	negative	negative	negative	negative
#N/A	807	Mbaa0729	M abscessus subsp abscessus	1155	GCA_900138245	Respiratory system		Positive	Positive	negative	negative	negative	negative
#N/A	765	Mbaa0730	M abscessus subsp abscessus	1156	GCA_900138235	Respiratory system		Positive	Positive	negative	negative	negative	negative
#N/A	292	Mbaa0731	M abscessus subsp abscessus	1157	GCA_900138055	Respiratory system		Positive	Positive	negative	negative	negative	negative
#N/A	770	Mbaa0732	M abscessus subsp abscessus	1158	GCA_900138075	Respiratory system		Positive	Positive	negative	negative	negative	negative
#N/A	42	Mbaa0733	M abscessus subsp abscessus	1159	GCA_900138115	Respiratory system		Positive	Positive	negative	negative	negative	negative
#N/A	776	Mbaa0734	M abscessus subsp abscessus	1160	GCA_900138105	Respiratory system		Positive	Positive	negative	negative	negative	negative
#N/A	293	Mbaa0735	M abscessus subsp abscessus	1161	GCA_900138125	Respiratory system		Positive	Positive	negative	negative	negative	negative
#N/A	835	Mbaa0736	M abscessus subsp abscessus	1162	GCA_900141595	Respiratory system		Positive	Positive	negative	negative	negative	negative
#N/A	347	Mbaa0737	M abscessus subsp abscessus	1163	GCA_900141465	Respiratory system		Positive	Positive	negative	negative	negative	negative

#N/A	606	Mbaa0738	M abscessus subsp abscessus	1165	GCA_900137175	Respiratory system	Positive	Positive	negative	negative	negative	negative
#N/A	513	Mbaa0739	M abscessus subsp abscessus	1169	GCA_900137145	Respiratory system	Positive	Positive	negative	negative	negative	negative
#N/A	511	Mbaa0740	M abscessus subsp abscessus	1170	GCA_900137165	Respiratory system	Positive	Positive	negative	negative	negative	negative
#N/A	512	Mbaa0741	M abscessus subsp abscessus	1171	GCA_900137155	Respiratory system	Positive	Positive	negative	negative	negative	negative
#N/A	961	Mbaa0742	M abscessus subsp abscessus	1557	GCA_002801495	sputum	Positive	Positive	negative	negative	negative	negative
#N/A	321	Mbaa0743	M abscessus subsp abscessus	1579	GCA_002801945	sputum	Positive	Positive	negative	negative	negative	negative
#N/A	1013	Mbaa0744	M abscessus subsp abscessus	1701	GCA_002802725	lavage fluid	Positive	Positive	negative	negative	negative	negative
#N/A	71	Mbaa0747	M abscessus subsp abscessus	2022	GCA_002800705	sputum	Positive	Positive	negative	negative	negative	negative
#N/A	327	Mbaa0748	M abscessus subsp abscessus	2272	GCA_002800345	sputum	Positive	Positive	negative	negative	negative	negative
#N/A	959	Mbaa0749	M abscessus subsp abscessus	2425	GCA_002802735	sputum	Positive	Positive	negative	negative	negative	negative
#N/A	967	Mbaa0751	M abscessus subsp abscessus	2833	GCA_002802385	sputum	Positive	Positive	negative	negative	negative	negative
#N/A	860	Mbaa0752	M abscessus subsp abscessus	2940	GCA_002800685	lavage fluid	Positive	Positive	negative	negative	negative	negative
#N/A	1005	Mbaa0753	M abscessus subsp abscessus	2950	GCA_002801665	sputum	Positive	Positive	negative	negative	negative	negative
#N/A	325	Mbaa0754	M abscessus subsp abscessus	3374	GCA_002800305	lavage fluid	Positive	Positive	negative	negative	negative	negative
#N/A	989	Mbaa0757	M abscessus subsp abscessus	3478	GCA_002800925	sputum	Positive	Positive	negative	negative	negative	negative
#N/A	585	Mbaa0759	M abscessus subsp abscessus	3671	GCA_002802285	sputum	Positive	Positive	negative	negative	negative	negative
#N/A	955	Mbaa0760	M abscessus subsp abscessus	3739	GCA_002801645	lavage fluid	Positive	Positive	negative	negative	negative	negative
#N/A	501	Mbaa0761	M abscessus subsp abscessus	3820	GCA_002801175	lavage fluid	Positive	Positive	negative	negative	negative	negative
#N/A	894	Mbaa0762	M abscessus subsp abscessus	3998	GCA_002801205	lavage fluid	Positive	Positive	negative	negative	negative	negative
#N/A	450	Mbaa0763	M abscessus subsp abscessus	4542	GCA_002802345	lavage fluid	Positive	Positive	negative	negative	negative	negative
#N/A	581	Mbaa0764	M abscessus subsp abscessus	4883	GCA_002800845	sputum	Positive	Positive	negative	negative	negative	negative
#N/A	582	Mbaa0765	M abscessus subsp abscessus	4886	GCA_002800815	sputum	Positive	Positive	negative	negative	negative	negative
#N/A	643	Mbaa0766	M abscessus subsp abscessus	5295	GCA_002801275	sputum	Positive	Positive	negative	negative	negative	negative
#N/A	1010	Mbaa0767	M abscessus subsp abscessus	5465	GCA_002801365	lavage fluid	Positive	Positive	negative	negative	negative	negative
#N/A	940	Mbaa0768	M abscessus subsp abscessus	5589	GCA_002801265	sputum	Positive	Positive	negative	negative	negative	negative
#N/A	965	Mbaa0769	M abscessus subsp abscessus	5681	GCA_002801485	sputum	Positive	Positive	negative	negative	negative	negative
#N/A	261	Mbaa0770	M abscessus subsp abscessus	5696	GCA_002802165	lavage fluid	Positive	Positive	negative	negative	negative	negative
#N/A	979	Mbaa0772	M abscessus subsp abscessus	5864	GCA_002802595	lavage fluid	Positive	Positive	negative	negative	negative	negative
#N/A	951	Mbaa0773	M abscessus subsp abscessus	6010	GCA_002801605	sputum	Positive	Positive	negative	negative	negative	negative
#N/A	970	Mbaa0774	M abscessus subsp abscessus	6094	GCA_002800885	lavage fluid	Positive	Positive	negative	negative	negative	negative
#N/A	199	Mbaa0776	M abscessus subsp abscessus	6449	GCA_002802135	lavage fluid	Positive	Positive	negative	negative	negative	negative
#N/A	863	Mbaa0778	M abscessus subsp abscessus	A137	GCA_002802075	sputum	Positive	Positive	negative	negative	negative	negative
#N/A	900	Mbaa0780	M abscessus subsp abscessus	A176	GCA_002801005	lavage fluid	Positive	Positive	negative	negative	negative	negative
#N/A	549	Mbaa0785	M abscessus subsp abscessus	A206	GCA_002800555	lavage fluid	Positive	Positive	negative	negative	negative	negative
#N/A	600	Mbaa0788	M abscessus subsp abscessus	A217	GCA_002802225	sputum	Positive	Positive	negative	negative	negative	negative
#N/A	966	Mbaa0790	M abscessus subsp abscessus	A229	GCA_002801465	sputum	Positive	negative	negative	negative	negative	negative
#N/A	904	Mbaa0792	M abscessus subsp abscessus	A233	GCA_002802565	lavage fluid	Positive	Positive	negative	negative	negative	negative
#N/A	273	Mbaa0795	M abscessus subsp abscessus	A249	GCA_002800525	sputum	Positive	Positive	negative	negative	negative	negative
#N/A	77	Mbaa0798	M abscessus subsp abscessus	A266	GCA_002800495	sputum	Positive	Positive	negative	negative	negative	negative
#N/A	652	Mbaa0801	M abscessus subsp abscessus	A305	GCA_002801085	sputum	Positive	Positive	negative	negative	negative	negative
#N/A	451	Mbaa0802	M abscessus subsp abscessus	A309	GCA_002802065	sputum	Positive	Positive	negative	negative	negative	negative
#N/A	853	Mbaa0803	M abscessus subsp abscessus	A311	GCA_002800485	lavage fluid	Positive	Positive	negative	negative	negative	negative
#N/A	65	Mbaa0804	M abscessus subsp abscessus	A312	GCA_002800465	lavage fluid	Positive	Positive	negative	negative	negative	negative
#N/A	878	Mbaa0806	M abscessus subsp abscessus	A317	GCA_002801065	lavage fluid	Positive	Positive	negative	negative	negative	negative
#N/A	889	Mbaa0808	M abscessus subsp abscessus	A324	GCA_002801025	sputum	negative	Positive	negative	negative	negative	negative
#N/A	74	Mbaa0813	M abscessus subsp abscessus	A350	GCA_002800445	sputum	Positive	Positive	negative	negative	negative	negative
#N/A	79	Mbaa0818	M abscessus subsp abscessus	A38	GCA_002800665	sputum	Positive	Positive	negative	negative	negative	negative
#N/A	944	Mbaa0819	M abscessus subsp abscessus	A395	GCA_002802805	lavage fluid	Positive	Positive	negative	negative	negative	negative
#N/A	982	Mbaa0820	M abscessus subsp abscessus	A40	GCA_002802425	lavage fluid	Positive	Positive	negative	negative	negative	negative
#N/A	892	Mbaa0821	M abscessus subsp abscessus	A426	GCA_002802515	sputum	Positive	Positive	negative	negative	negative	negative
#N/A	198	Mbaa0822	M abscessus subsp abscessus	A467	GCA_002800405	sputum	Positive	Positive	negative	negative	negative	negative
#N/A	1019	Mbaa0824	M abscessus subsp abscessus	A478	GCA_002802505	sputum	Positive	Positive	negative	negative	negative	negative
#N/A	274	Mbaa0825	M abscessus subsp abscessus	A482	GCA_002802025	lavage fluid	Positive	Positive	negative	negative	negative	negative

#N/A	557	Mbaa0826	M abscessus subsp abscessus	A49	GCA_002802405	lavage fluid	Positive	Positive	negative	negative	negative	negative
#N/A	67	Mbaa0827	M abscessus subsp abscessus	A51	GCA_002800625	sputum	Positive	Positive	negative	negative	negative	negative
#N/A	275	Mbaa0829	M abscessus subsp abscessus	A58	GCA_002802145	sputum	Positive	Positive	negative	negative	negative	negative
#N/A	846	Mbaa0832	M abscessus subsp abscessus	A73	GCA_002800615	sputum	Positive	Positive	negative	negative	negative	negative
#N/A	96	Mbaa0833	M abscessus subsp abscessus	A79	GCA_002802055	sputum	Positive	Positive	negative	negative	negative	negative
#N/A	403	Mbaa0834	M abscessus subsp abscessus	A8	GCA_002800165	sputum	Positive	Positive	negative	negative	negative	negative
#N/A	197	Mbaa0844	M abscessus subsp bolletii	103	GCA_000524075	sputum	Positive	Positive	negative	negative	negative	negative
#N/A	539	Mbaa0847	M abscessus subsp bolletii	137	GCA_001792645	respiratory	Positive	Positive	negative	negative	negative	negative
#N/A	954	Mbaa1308	M abscessus	199	GCA_003076835	Not available	Positive	Positive	negative	negative	negative	negative
#N/A	485	Mbaa1312	M abscessus	9808	GCA_000332605	wound exudate	Positive	Positive	negative	negative	negative	negative
#N/A	213	Mbaa1313	M abscessus	3A-0119-R	GCA_000270825	sputum	Positive	Positive	negative	negative	negative	negative
#N/A	215	Mbaa1315	M abscessus	3A-0122-S	GCA_000270585	sputum	Positive	Positive	negative	negative	negative	negative
#N/A	217	Mbaa1316	M abscessus	3A-0731	GCA_000270705	sputum	Positive	Positive	negative	negative	negative	negative
#N/A	214	Mbaa1318	M abscessus	3A-0930-R	GCA_000270565	sputum	Positive	Positive	negative	negative	negative	negative
#N/A	216	Mbaa1319	M abscessus	3A-0930-S	GCA_000270725	sputum	Positive	Positive	negative	negative	negative	negative
#N/A	655	Mbaa1322	M abscessus	4S-0116-S	GCA_000270625	sputum	Positive	Positive	negative	negative	negative	negative
#N/A	659	Mbaa1323	M abscessus	4S-0206	GCA_000270745	sputum	Positive	Positive	negative	negative	negative	negative
#N/A	657	Mbaa1324	M abscessus	4S-0303	GCA_000271125	sputum	Positive	Positive	negative	negative	negative	negative
#N/A	656	Mbaa1325	M abscessus	4S-0726-RA	GCA_000271245	sputum	Positive	Positive	negative	negative	negative	negative
#N/A	210	Mbaa1335	M abscessus	6G-0125-R	GCA_000270985	sputum	Positive	Positive	negative	negative	negative	negative
#N/A	211	Mbaa1336	M abscessus	6G-0125-S	GCA_000270865	sputum	Positive	Positive	negative	negative	negative	negative
#N/A	212	Mbaa1339	M abscessus	6G-0728-S	GCA_000271185	sputum	Positive	Positive	negative	negative	negative	negative
#N/A	595	Mbaa1342	M abscessus	A137	GCA_003071695	sputum	Positive	Positive	negative	negative	negative	negative
#N/A	279	Mbaa1344	M abscessus	A247	GCA_003071935	sputum	Positive	Positive	negative	negative	negative	negative
#N/A	145	Mbaa1345	M abscessus	A254	GCA_003072325	Bronchoalveolar lavage	Positive	Positive	negative	negative	negative	negative
#N/A	76	Mbaa1346	M abscessus	A305	GCA_003072425	sputum	Positive	Positive	negative	negative	negative	negative
#N/A	308	Mbaa1347	M abscessus	A315	GCA_003072385	sputum	Positive	Positive	negative	negative	negative	negative
#N/A	550	Mbaa1348	M abscessus	A321	GCA_003072405	sputum	Positive	Positive	negative	negative	negative	negative
#N/A	73	Mbaa1349	M abscessus	A73	GCA_003071685	Bronchoalveolar lavage	Positive	Positive	negative	negative	negative	negative
#N/A	569	Mbaa1350	M abscessus	CF	GCA_000333695	Not available	Positive	Positive	negative	negative	negative	negative
#N/A	786	Mbaa1351	M abscessus	CF34-14-1	GCA_004010025	Homo sapiens	Positive	Positive	negative	negative	negative	negative
#N/A	66	Mbaa1360	M abscessus	FLAC014	GCA_002142865	Homo sapiens	Positive	Positive	negative	negative	negative	negative
#N/A	117	Mbaa1368	M abscessus	FLAC046	GCA_001677195	Homo sapiens	Positive	Positive	negative	negative	negative	negative
#N/A	118	Mbaa1374	M abscessus	FLAC053	GCA_002142775	Homo sapiens	Positive	Positive	negative	negative	negative	negative
#N/A	901	Mbaa1378	M abscessus	G102	GCA_003583085	sputum	Positive	Positive	negative	negative	negative	negative
#N/A	950	Mbaa1379	M abscessus	G104	GCA_003583065	sputum	Positive	Positive	negative	negative	negative	negative
#N/A	316	Mbaa1381	M abscessus	G106	GCA_003583025	sputum	Positive	negative	negative	negative	negative	negative
#N/A	537	Mbaa1383	M abscessus	G108	GCA_003582985	Bronchoalveolar lavage	Positive	Positive	negative	negative	negative	negative
#N/A	949	Mbaa1384	M abscessus	G109	GCA_003582965	sputum	Positive	Positive	negative	negative	negative	negative
#N/A	867	Mbaa1386	M abscessus	G111	GCA_003582905	sputum	Positive	Positive	negative	negative	negative	negative
#N/A	973	Mbaa1387	M abscessus	G112	GCA_003072285	sputum	Positive	Positive	negative	negative	negative	negative
#N/A	570	Mbaa1388	M abscessus	G113	GCA_003582895	sputum	Positive	Positive	negative	negative	negative	negative
#N/A	314	Mbaa1389	M abscessus	G114	GCA_003072235	Bronchoalveolar lavage	Positive	negative	negative	negative	negative	negative
#N/A	910	Mbaa1390	M abscessus	G115	GCA_003582885	Bronchoalveolar lavage	Positive	Positive	negative	negative	negative	negative
#N/A	278	Mbaa1392	M abscessus	G119	GCA_003071865	sputum	Positive	Positive	negative	negative	negative	negative
#N/A	587	Mbaa1393	M abscessus	G120	GCA_003582845	sputum	Positive	Positive	negative	negative	negative	negative
#N/A	592	Mbaa1394	M abscessus	G121	GCA_003582825	Bronchoalveolar lavage	Positive	Positive	negative	negative	negative	negative
#N/A	864	Mbaa1395	M abscessus	G122	GCA_003076815	Not available	Positive	Positive	negative	negative	negative	negative
#N/A	280	Mbaa1396	M abscessus	G123	GCA_003582795	sputum	Positive	Positive	negative	negative	negative	negative
#N/A	601	Mbaa1398	M abscessus	G125	GCA_003582765	sputum	Positive	Positive	negative	negative	negative	negative
#N/A	995	Mbaa1400	M abscessus	G127	GCA_003581705	sputum	Positive	Positive	negative	negative	negative	negative
#N/A	902	Mbaa1401	M abscessus	G128	GCA_003582715	sputum	Positive	Positive	negative	negative	negative	negative
#N/A	644	Mbaa1402	M abscessus	G133	GCA_003582705	sputum	Positive	Positive	negative	negative	negative	negative

#N/A	322	Mbaa1403	M abscessus	G134	GCA_003582685	sputum	Positive	Positive	negative	negative	negative	negative
#N/A	876	Mbaa1406	M abscessus	G139	GCA_003582625	Bronchoalveolar lavage	Positive	Positive	negative	negative	negative	negative
#N/A	877	Mbaa1407	M abscessus	G140	GCA_003582605	sputum	Positive	Positive	negative	negative	negative	negative
#N/A	323	Mbaa1409	M abscessus	G142	GCA_003582575	Bronchoalveolar lavage	Positive	Positive	negative	negative	negative	negative
#N/A	945	Mbaa1410	M abscessus	G143	GCA_003582565	sputum	Positive	Positive	negative	negative	negative	negative
#N/A	890	Mbaa1411	M abscessus	G146	GCA_003582545	Bronchoalveolar lavage	Positive	Positive	negative	negative	negative	negative
#N/A	510	Mbaa1413	M abscessus	G148	GCA_003071765	Bronchoalveolar lavage	Positive	Positive	negative	negative	negative	negative
#N/A	847	Mbaa1414	M abscessus	G149	GCA_003582525	sputum	Positive	Positive	negative	negative	Positive	negative
#N/A	223	Mbaa1415	M abscessus	G152	GCA_003071825	sputum	Positive	Positive	negative	negative	negative	negative
#N/A	893	Mbaa1417	M abscessus	G155	GCA_003582485	sputum	Positive	Positive	negative	negative	negative	negative
#N/A	75	Mbaa1419	M abscessus	G158	GCA_003582445	sputum	Positive	Positive	negative	negative	negative	negative
#N/A	993	Mbaa1420	M abscessus	G159	GCA_003581665	sputum	Positive	Positive	negative	negative	negative	negative
#N/A	441	Mbaa1422	M abscessus	G161	GCA_003072225	sputum	Positive	Positive	negative	negative	negative	negative
#N/A	994	Mbaa1423	M abscessus	G162	GCA_003071805	sputum	Positive	Positive	negative	negative	negative	negative
#N/A	991	Mbaa1425	M abscessus	G169	GCA_003582405	sputum	Positive	Positive	negative	negative	negative	negative
#N/A	315	Mbaa1426	M abscessus	G170	GCA_003071745	sputum	Positive	negative	negative	negative	negative	negative
#N/A	387	Mbaa1428	M abscessus	G174	GCA_003582365	sputum	Positive	Positive	negative	negative	negative	negative
#N/A	971	Mbaa1429	M abscessus	G176	GCA_003582345	sputum	Positive	Positive	negative	negative	negative	negative
#N/A	976	Mbaa1430	M abscessus	G177	GCA_003582325	Bronchoalveolar lavage	Positive	Positive	negative	negative	negative	negative
#N/A	947	Mbaa1431	M abscessus	G178	GCA_003582295	sputum	Positive	Positive	negative	negative	negative	negative
#N/A	324	Mbaa1432	M abscessus	G179	GCA_003582285	sputum	Positive	Positive	negative	negative	negative	negative
#N/A	972	Mbaa1433	M abscessus	G180	GCA_003582265	Bronchoalveolar lavage	Positive	Positive	negative	negative	negative	negative
#N/A	311	Mbaa1434	M abscessus	G181	GCA_003582235	Bronchoalveolar lavage	Positive	negative	negative	negative	negative	negative
#N/A	948	Mbaa1435	M abscessus	G182	GCA_003072205	sputum	Positive	Positive	negative	negative	negative	negative
#N/A	891	Mbaa1436	M abscessus	G184	GCA_003582225	sputum	Positive	Positive	negative	negative	negative	negative
#N/A	384	Mbaa1437	M abscessus	G185	GCA_003582205	sputum	Positive	Positive	negative	negative	negative	negative
#N/A	593	Mbaa1440	M abscessus	G192	GCA_003582145	sputum	Positive	Positive	negative	negative	negative	negative
#N/A	999	Mbaa1441	M abscessus	G193	GCA_003582115	sputum	Positive	Positive	negative	negative	negative	negative
#N/A	313	Mbaa1442	M abscessus	G194	GCA_003582105	sputum	Positive	negative	negative	negative	negative	negative
#N/A	312	Mbaa1443	M abscessus	G195	GCA_003582075	sputum	Positive	negative	negative	negative	negative	negative
#N/A	442	Mbaa1444	M abscessus	G197	GCA_003582055	sputum	Positive	Positive	negative	negative	negative	negative
#N/A	962	Mbaa1445	M abscessus	G198	GCA_003582045	sputum	Positive	Positive	negative	negative	negative	negative
#N/A	319	Mbaa1446	M abscessus	G200	GCA_003582025	sputum	Positive	negative	negative	negative	negative	negative
#N/A	956	Mbaa1449	M abscessus	G204	GCA_003581985	Bronchoalveolar lavage	Positive	Positive	negative	negative	negative	negative
#N/A	968	Mbaa1450	M abscessus	G205	GCA_003581945	sputum	Positive	Positive	negative	negative	negative	negative
#N/A	309	Mbaa1452	M abscessus	G208	GCA_003581935	sputum	Positive	Positive	negative	negative	negative	negative
#N/A	594	Mbaa1453	M abscessus	G213	GCA_003581675	sputum	Positive	Positive	negative	negative	negative	negative
#N/A	871	Mbaa1454	M abscessus	G215	GCA_003581925	sputum	Positive	Positive	negative	negative	negative	negative
#N/A	879	Mbaa1455	M abscessus	G216	GCA_003581895	sputum	Positive	Positive	negative	negative	negative	negative
#N/A	1004	Mbaa1456	M abscessus	G218	GCA_003071785	sputum	Positive	Positive	negative	negative	negative	negative
#N/A	952	Mbaa1457	M abscessus	G219	GCA_003072145	sputum	Positive	Positive	negative	negative	negative	negative
#N/A	841	Mbaa1459	M abscessus	G70	GCA_003581885	sputum	Positive	Positive	negative	negative	negative	negative
#N/A	895	Mbaa1460	M abscessus	G72	GCA_003581865	sputum	Positive	Positive	negative	negative	negative	negative
#N/A	946	Mbaa1461	M abscessus	G73	GCA_003581845	sputum	Positive	Positive	negative	negative	negative	negative
#N/A	896	Mbaa1463	M abscessus	G76	GCA_003581805	sputum	Positive	Positive	negative	negative	negative	negative
#N/A	276	Mbaa1465	M abscessus	G82	GCA_003581785	sputum	Positive	Positive	negative	negative	negative	negative
#N/A	1009	Mbaa1466	M abscessus	G84	GCA_003581765	sputum	Positive	Positive	negative	negative	negative	negative
#N/A	68	Mbaa1468	M abscessus	G86	GCA_003581735	sputum	Positive	Positive	negative	negative	negative	negative
#N/A	903	Mbaa1470	M abscessus	G89	GCA_003581725	sputum	Positive	Positive	negative	negative	negative	negative
#N/A	963	Mbaa1471	M abscessus	G90	GCA_003580165	sputum	Positive	Positive	negative	negative	negative	negative
#N/A	953	Mbaa1472	M abscessus	G91	GCA_003071975	Bronchoalveolar lavage	Positive	Positive	negative	negative	negative	negative
#N/A	591	Mbaa1473	M abscessus	G94	GCA_003071845	sputum	Positive	Positive	negative	negative	negative	negative
#N/A	584	Mbaa1475	M abscessus	G99	GCA_003580125	sputum	Positive	Positive	negative	negative	negative	negative

#N/A	1017	Mbaa1491	M abscessus	MAB_030201_1075	GCA_000523575	bronchoalveolar lavage	Positive	Positive	negative	negative	negative	negative
#N/A	1015	Mbaa1496	M abscessus	MAB_110811_1470	GCA_000523955	sputum	Positive	Positive	negative	negative	negative	negative
#N/A	83	Mbaa1500	M abscessus	NOV0213	GCA_001430775	sputum	Positive	Positive	negative	negative	negative	negative
#N/A	110	Mbaa1501	M abscessus	PAP001	GCA_001213205	sputum	Positive	Positive	negative	negative	negative	negative
#N/A	109	Mbaa1502	M abscessus	PAP002	GCA_001212845	sputum	Positive	Positive	negative	negative	negative	negative
#N/A	686	Mbaa1506	M abscessus	PAP008	GCA_001213945	sputum	Positive	Positive	negative	negative	negative	negative
#N/A	554	Mbaa1508	M abscessus	PAP010	GCA_001212985	sputum	Positive	Positive	negative	negative	negative	negative
#N/A	801	Mbaa1509	M abscessus	PAP011	GCA_001213725	sputum	Positive	Positive	negative	negative	negative	negative
#N/A	683	Mbaa1510	M abscessus	PAP012	GCA_001213385	sputum	Positive	Positive	negative	negative	negative	negative
#N/A	802	Mbaa1517	M abscessus	PAP019	GCA_001214185	sputum	Positive	Positive	negative	negative	negative	negative
#N/A	111	Mbaa1518	M abscessus	PAP020	GCA_001213825	sputum	Positive	Positive	negative	negative	negative	negative
#N/A	555	Mbaa1519	M abscessus	PAP022	GCA_001213165	sputum	Positive	Positive	negative	negative	negative	negative
#N/A	798	Mbaa1526	M abscessus	PAP029	GCA_001212865	sputum	Positive	Positive	negative	negative	negative	negative
#N/A	796	Mbaa1527	M abscessus	PAP030	GCA_001213965	sputum	Positive	Positive	negative	negative	negative	negative
#N/A	684	Mbaa1529	M abscessus	PAP032	GCA_001213685	sputum	Positive	Positive	negative	negative	negative	negative
#N/A	419	Mbaa1532	M abscessus	PAP036	GCA_001213605	sputum	Positive	Positive	negative	negative	negative	negative
#N/A	414	Mbaa1536	M abscessus	PAP040	GCA_001213225	sputum	Positive	Positive	negative	negative	negative	negative
#N/A	417	Mbaa1537	M abscessus	PAP041	GCA_001213745	sputum	Positive	Positive	negative	negative	negative	negative
#N/A	800	Mbaa1538	M abscessus	PAP042	GCA_001212805	sputum	Positive	Positive	negative	negative	negative	negative
#N/A	803	Mbaa1542	M abscessus	PAP046	GCA_001214085	sputum	Positive	Positive	negative	negative	negative	negative
#N/A	797	Mbaa1543	M abscessus	PAP047	GCA_001213505	sputum	Positive	Positive	negative	negative	negative	negative
#N/A	814	Mbaa1549	M abscessus	PAP054	GCA_001215625	sputum	Positive	Positive	negative	negative	negative	negative
#N/A	804	Mbaa1554	M abscessus	PAP060	GCA_001212885	sputum	Positive	Positive	negative	negative	negative	negative
#N/A	768	Mbaa1555	M abscessus	PAP062	GCA_001213885	sputum	Positive	Positive	negative	negative	negative	negative
#N/A	793	Mbaa1559	M abscessus	PAP067	GCA_001213705	sputum	Positive	Positive	negative	negative	negative	negative
#N/A	791	Mbaa1560	M abscessus	PAP068	GCA_001213785	sputum	Positive	Positive	negative	negative	negative	negative
#N/A	819	Mbaa1561	M abscessus	PAP069	GCA_001213285	sputum	Positive	Positive	negative	negative	negative	negative
#N/A	794	Mbaa1563	M abscessus	PAP071	GCA_001214985	sputum	Positive	Positive	negative	negative	negative	negative
#N/A	748	Mbaa1567	M abscessus	PAP075	GCA_001215445	sputum	Positive	Positive	negative	negative	negative	negative
#N/A	415	Mbaa1568	M abscessus	PAP076	GCA_001215845	sputum	Positive	Positive	negative	negative	negative	negative
#N/A	108	Mbaa1569	M abscessus	PAP077	GCA_001215045	sputum	Positive	Positive	negative	negative	negative	negative
#N/A	685	Mbaa1571	M abscessus	PAP079	GCA_001217145	sputum	Positive	Positive	negative	negative	negative	negative
#N/A	413	Mbaa1574	M abscessus	PAP082	GCA_001215385	sputum	Positive	Positive	negative	negative	negative	negative
#N/A	792	Mbaa1575	M abscessus	PAP083	GCA_001216305	sputum	Positive	Positive	negative	negative	negative	negative
#N/A	405	Mbaa1577	M abscessus	PAP085	GCA_001214625	sputum	Positive	Positive	negative	negative	negative	negative
#N/A	873	Mbaa1582	M abscessus	PAP090	GCA_001217125	sputum	Positive	Positive	negative	negative	negative	negative
#N/A	815	Mbaa1594	M abscessus	PAP102	GCA_001215005	sputum	Positive	Positive	negative	negative	negative	negative
#N/A	813	Mbaa1598	M abscessus	PAP106	GCA_001215565	sputum	Positive	Positive	negative	negative	negative	negative
#N/A	439	Mbaa1605	M abscessus	PAP113	GCA_001216145	sputum	Positive	Positive	negative	negative	negative	negative
#N/A	426	Mbaa1607	M abscessus	PAP115	GCA_001215965	sputum	Positive	Positive	negative	negative	negative	negative
#N/A	440	Mbaa1619	M abscessus	PAP127	GCA_001350895	sputum	Positive	Positive	negative	negative	negative	negative
#N/A	805	Mbaa1622	M abscessus	PAP130	GCA_001217525	sputum	Positive	Positive	negative	negative	negative	negative
#N/A	799	Mbaa1624	M abscessus	PAP132	GCA_001215265	sputum	Positive	Positive	negative	negative	negative	negative
#N/A	816	Mbaa1625	M abscessus	PAP133	GCA_001214585	sputum	Positive	Positive	negative	negative	negative	negative
#N/A	556	Mbaa1630	M abscessus	PAP138	GCA_001216065	sputum	Positive	Positive	negative	negative	negative	negative
#N/A	558	Mbaa1632	M abscessus	PAP140	GCA_001215165	sputum	Positive	Positive	negative	negative	negative	negative
#N/A	817	Mbaa1652	M abscessus	PAP161	GCA_001214925	sputum	Positive	Positive	negative	negative	negative	negative
#N/A	822	Mbaa1653	M abscessus	PAP163	GCA_001217185	sputum	Positive	Positive	negative	negative	negative	negative
#N/A	427	Mbaa1658	M abscessus	PAP170	GCA_001215945	sputum	Positive	Positive	negative	negative	negative	negative
#N/A	158	Mbaa1662	M abscessus	V06705	GCA_000443145	bronchoalveolar lavage	Positive	Positive	negative	negative	negative	negative
#N/A	1585	Mbab0841	M abscessus subsp bolletii	17	GCA_900139385	Respiratory system	negative	Positive	Positive	Positive	negative	negative
#N/A	1625	Mbab0845	M abscessus subsp bolletii	107	GCA_900136655	Respiratory system	negative	negative	Positive	Positive	negative	negative
#N/A	1602	Mbab0846	M abscessus subsp bolletii	135	GCA_900139545	Respiratory system	negative	negative	Positive	Positive	negative	negative

#N/A	1564	Mbab0851	M abscessus subsp bolletii	174	GCA_900130515	Respiratory system	negative	negative	Positive	Positive	negative	negative
#N/A	1557	Mbab0852	M abscessus subsp bolletii	185	GCA_900130615	Respiratory system	negative	negative	Positive	Positive	negative	negative
#N/A	1565	Mbab0854	M abscessus subsp bolletii	202	GCA_900130815	Respiratory system	negative	negative	Positive	Positive	negative	negative
#N/A	1623	Mbab0861	M abscessus subsp bolletii	240	GCA_900131255	Respiratory system	negative	negative	Positive	Positive	negative	negative
#N/A	1558	Mbab0862	M abscessus subsp bolletii	244	GCA_900131295	Respiratory system	negative	negative	Positive	Positive	negative	negative
#N/A	1620	Mbab0864	M abscessus subsp bolletii	268	GCA_900131565	Respiratory system	negative	negative	Positive	Positive	negative	negative
#N/A	1559	Mbab0867	M abscessus subsp bolletii	295	GCA_900131955	Respiratory system	negative	negative	Positive	Positive	negative	negative
#N/A	1560	Mbab0868	M abscessus subsp bolletii	305	GCA_900132055	Respiratory system	negative	negative	Positive	Positive	negative	negative
#N/A	1616	Mbab0870	M abscessus subsp bolletii	316	GCA_900132285	Respiratory system	negative	negative	Positive	Positive	negative	negative
#N/A	1614	Mbab0872	M abscessus subsp bolletii	321	GCA_900132345	Respiratory system	negative	negative	Positive	Positive	negative	negative
#N/A	1615	Mbab0873	M abscessus subsp bolletii	322	GCA_900132355	Respiratory system	negative	negative	Positive	Positive	negative	negative
#N/A	1561	Mbab0875	M abscessus subsp bolletii	334	GCA_900132455	Respiratory system	negative	negative	Positive	Positive	negative	negative
#N/A	1562	Mbab0877	M abscessus subsp bolletii	344	GCA_900132785	Respiratory system	negative	negative	Positive	Positive	negative	negative
#N/A	1622	Mbab0880	M abscessus subsp bolletii	377	GCA_900133335	Respiratory system	negative	negative	Positive	Positive	negative	negative
#N/A	1572	Mbab0883	M abscessus subsp bolletii	411	GCA_900134105	Respiratory system	negative	negative	Positive	Positive	negative	negative
#N/A	1570	Mbab0884	M abscessus subsp bolletii	432	GCA_900135035	Respiratory system	negative	negative	Positive	Positive	negative	negative
#N/A	1569	Mbab0885	M abscessus subsp bolletii	442	GCA_900134755	Respiratory system	negative	negative	Positive	Positive	negative	negative
#N/A	1606	Mbab0888	M abscessus subsp bolletii	457	GCA_900134915	Respiratory system	negative	negative	Positive	Positive	negative	negative
#N/A	1607	Mbab0889	M abscessus subsp bolletii	458	GCA_900134925	Respiratory system	negative	negative	Positive	Positive	negative	negative
#N/A	1595	Mbab0894	M abscessus subsp bolletii	512	GCA_900139985	Respiratory system	negative	negative	Positive	Positive	negative	negative
#N/A	1580	Mbab0898	M abscessus subsp bolletii	528	GCA_900139995	Respiratory system	negative	Positive	Positive	Positive	negative	negative
#N/A	1579	Mbab0899	M abscessus subsp bolletii	532	GCA_900140205	Respiratory system	negative	negative	Positive	Positive	negative	negative
#N/A	1581	Mbab0900	M abscessus subsp bolletii	536	GCA_900140215	Respiratory system	negative	Positive	Positive	Positive	negative	negative
#N/A	1662	Mbab0903	M abscessus subsp bolletii	748	GCA_900130975	Respiratory system	negative	negative	Positive	Positive	negative	negative
#N/A	1656	Mbab0904	M abscessus subsp bolletii	749	GCA_900130995	Respiratory system	negative	negative	Positive	Positive	negative	negative
#N/A	1658	Mbab0905	M abscessus subsp bolletii	750	GCA_900131455	Respiratory system	negative	negative	Positive	Positive	negative	negative
#N/A	1657	Mbab0906	M abscessus subsp bolletii	751	GCA_900131775	Respiratory system	negative	negative	Positive	Positive	negative	negative
#N/A	1655	Mbab0907	M abscessus subsp bolletii	752	GCA_900131795	Respiratory system	negative	negative	Positive	Positive	negative	negative
#N/A	1660	Mbab0908	M abscessus subsp bolletii	753	GCA_900132165	Respiratory system	negative	negative	Positive	Positive	negative	negative
#N/A	1659	Mbab0909	M abscessus subsp bolletii	754	GCA_900132465	Respiratory system	negative	negative	Positive	Positive	negative	negative
#N/A	1639	Mbab0910	M abscessus subsp bolletii	796	GCA_900131835	Respiratory system	negative	negative	Positive	Positive	negative	negative
#N/A	1652	Mbab0911	M abscessus subsp bolletii	845	GCA_900132985	Respiratory system	negative	negative	Positive	Positive	negative	negative
#N/A	1647	Mbab0912	M abscessus subsp bolletii	846	GCA_900134275	Respiratory system	negative	negative	Positive	Positive	negative	negative
#N/A	1648	Mbab0913	M abscessus subsp bolletii	847	GCA_900134285	Respiratory system	negative	negative	Positive	Positive	negative	negative
#N/A	1646	Mbab0914	M abscessus subsp bolletii	848	GCA_900134295	Respiratory system	negative	negative	Positive	Positive	negative	negative
#N/A	1649	Mbab0915	M abscessus subsp bolletii	850	GCA_900137135	Respiratory system	negative	negative	Positive	Positive	negative	negative
#N/A	1650	Mbab0916	M abscessus subsp bolletii	874	GCA_900135885	Respiratory system	negative	negative	Positive	Positive	negative	negative
#N/A	1651	Mbab0917	M abscessus subsp bolletii	875	GCA_900135895	Respiratory system	negative	negative	Positive	Positive	negative	negative
#N/A	1594	Mbab0919	M abscessus subsp bolletii	909	GCA_900133045	Respiratory system	negative	negative	Positive	Positive	Positive	negative
#N/A	1590	Mbab0920	M abscessus subsp bolletii	912	GCA_900133055	Respiratory system	negative	negative	Positive	Positive	negative	negative
#N/A	1591	Mbab0921	M abscessus subsp bolletii	920	GCA_900133105	Respiratory system	negative	negative	Positive	Positive	negative	negative
#N/A	1599	Mbab0922	M abscessus subsp bolletii	927	GCA_900133795	Respiratory system	negative	negative	Positive	Positive	negative	negative
#N/A	1592	Mbab0923	M abscessus subsp bolletii	933	GCA_900133625	Respiratory system	negative	negative	Positive	Positive	negative	negative
#N/A	1589	Mbab0924	M abscessus subsp bolletii	934	GCA_900133635	Respiratory system	negative	negative	Positive	Positive	negative	negative
#N/A	1593	Mbab0925	M abscessus subsp bolletii	946	GCA_900133785	Respiratory system	negative	negative	Positive	Positive	negative	negative
#N/A	1587	Mbab0926	M abscessus subsp bolletii	954	GCA_900134335	Respiratory system	negative	negative	Positive	Positive	negative	negative
#N/A	1575	Mbab0927	M abscessus subsp bolletii	959	GCA_900134425	Respiratory system	negative	negative	Positive	Positive	negative	negative
#N/A	1588	Mbab0928	M abscessus subsp bolletii	968	GCA_900134535	Respiratory system	negative	negative	Positive	Positive	negative	negative
#N/A	1584	Mbab0929	M abscessus subsp bolletii	979	GCA_900135995	Respiratory system	negative	Positive	Positive	Positive	negative	negative
#N/A	1582	Mbab0930	M abscessus subsp bolletii	1005	GCA_900136205	Respiratory system	negative	Positive	Positive	Positive	negative	negative
#N/A	1583	Mbab0931	M abscessus subsp bolletii	1008	GCA_900136235	Respiratory system	negative	Positive	Positive	Positive	negative	negative
#N/A	1577	Mbab0932	M abscessus subsp bolletii	1022	GCA_900137195	Respiratory system	negative	negative	Positive	Positive	negative	negative
#N/A	1576	Mbab0933	M abscessus subsp bolletii	1033	GCA_900137855	Respiratory system	negative	negative	Positive	Positive	negative	negative

#N/A	1600	Mbab0934	M abscessus subsp bolletii	1056	GCA_900137535	Respiratory system	negative	negative	Positive	Positive	negative	negative
#N/A	1661	Mbab0935	M abscessus subsp bolletii	1107	GCA_900141605	Respiratory system	negative	negative	Positive	Positive	negative	negative
#N/A	1578	Mbab0936	M abscessus subsp bolletii	1128	GCA_900137885	Respiratory system	negative	negative	Positive	Positive	negative	negative
#N/A	1638	Mbab1511	M abscessus	PAP013	GCA_001212905	sputum	negative	negative	Positive	Positive	negative	negative
#N/A	1626	Mbab1512	M abscessus	PAP014	GCA_001213985	sputum	negative	negative	Positive	Positive	negative	negative
#N/A	1631	Mbab1513	M abscessus	PAP015	GCA_001213305	sputum	negative	negative	Positive	Positive	negative	negative
#N/A	1627	Mbab1530	M abscessus	PAP033	GCA_001213665	sputum	negative	negative	Positive	Positive	negative	negative
#N/A	1632	Mbab1534	M abscessus	PAP038	GCA_001213445	sputum	negative	negative	Positive	Positive	negative	negative
#N/A	1630	Mbab1540	M abscessus	PAP044	GCA_001213845	sputum	negative	negative	Positive	Positive	negative	negative
#N/A	1633	Mbab1556	M abscessus	PAP063	GCA_001214065	sputum	negative	negative	Positive	Positive	negative	negative
#N/A	1634	Mbab1557	M abscessus	PAP064	GCA_001214045	sputum	negative	negative	Positive	Positive	negative	negative
#N/A	1636	Mbab1558	M abscessus	PAP065	GCA_001214105	sputum	negative	negative	Positive	Positive	negative	negative
#N/A	1608	Mbab1591	M abscessus	PAP099	GCA_001216025	sputum	negative	negative	Positive	Positive	negative	negative
#N/A	1628	Mbab1623	M abscessus	PAP131	GCA_001215465	sputum	negative	negative	Positive	Positive	negative	negative
#N/A	1635	Mbab1631	M abscessus	PAP139	GCA_001216965	sputum	negative	negative	Positive	Positive	negative	negative
#N/A	1629	Mbab1634	M abscessus	PAP142	GCA_001216265	sputum	negative	negative	Positive	Positive	negative	negative
#N/A	1637	Mbab1651	M abscessus	PAP160	GCA_001214225	sputum	negative	negative	Positive	Positive	negative	negative
#N/A	1466	Mbam0918	M abscessus subsp bolletii	893	GCA_900141415	Respiratory system	negative	negative	negative	negative	Positive	Positive
#N/A	1302	Mbam0939	M abscessus subsp bolletii	1S-151-0930	GCA_000271165	bronchial alveolar lavage	negative	negative	negative	negative	Positive	Positive
#N/A	1293	Mbam0942	M abscessus subsp bolletii	1S-154-0310	GCA_000308015	bronchial alveolar lavage	negative	negative	negative	negative	Positive	Positive
#N/A	1191	Mbam0946	M abscessus subsp bolletii	2B-0912-R	GCA_000270805	sputum	negative	negative	negative	negative	Positive	Positive
#N/A	1192	Mbam0948	M abscessus subsp bolletii	2B-1231	GCA_000270925	bronchial alveolar lavage	negative	negative	negative	negative	Positive	Positive
#N/A	1311	Mbam0958	M abscessus subsp massiliense	59	GCA_002801925	sputum	negative	negative	negative	negative	Positive	Positive
#N/A	1546	Mbam0962	M abscessus subsp massiliense	75	GCA_900132825	Respiratory system	negative	negative	negative	negative	Positive	Positive
#N/A	1091	Mbam0963	M abscessus subsp massiliense	76	GCA_900133475	Respiratory system	negative	negative	negative	negative	Positive	Positive
#N/A	1446	Mbam0964	M abscessus subsp massiliense	84	GCA_900136635	Respiratory system	negative	negative	negative	negative	Positive	Positive
#N/A	1361	Mbam0965	M abscessus subsp massiliense	93	GCA_900139645	Respiratory system	negative	negative	negative	negative	Positive	Positive
#N/A	1362	Mbam0967	M abscessus subsp massiliense	112	GCA_900136725	Respiratory system	negative	negative	negative	negative	Positive	Positive
#N/A	1363	Mbam0968	M abscessus subsp massiliense	113	GCA_900136715	Respiratory system	negative	negative	negative	negative	Positive	Positive
#N/A	1354	Mbam0972	M abscessus subsp massiliense	130	GCA_002801855	sputum	negative	negative	negative	negative	Positive	Positive
#N/A	1351	Mbam0973	M abscessus subsp massiliense	130	GCA_900139525	Respiratory system	negative	negative	negative	negative	Positive	Positive
#N/A	1344	Mbam0975	M abscessus subsp massiliense	139	GCA_900139605	Respiratory system	negative	negative	negative	negative	Positive	Positive
#N/A	1098	Mbam0976	M abscessus subsp massiliense	145	GCA_900130275	Respiratory system	negative	negative	negative	negative	Positive	Positive
#N/A	1493	Mbam0977	M abscessus subsp massiliense	147	GCA_900130305	Respiratory system	negative	negative	negative	negative	Positive	Positive
#N/A	1096	Mbam0978	M abscessus subsp massiliense	149	GCA_900130325	Respiratory system	negative	negative	negative	negative	Positive	Positive
#N/A	1494	Mbam0979	M abscessus subsp massiliense	150	GCA_900130335	Respiratory system	negative	negative	negative	negative	Positive	Positive
#N/A	1495	Mbam0980	M abscessus subsp massiliense	151	GCA_900130345	Respiratory system	negative	negative	negative	negative	Positive	Positive
#N/A	1496	Mbam0983	M abscessus subsp massiliense	154	GCA_900130365	Respiratory system	negative	negative	negative	negative	Positive	Positive
#N/A	1497	Mbam0984	M abscessus subsp massiliense	155	GCA_900130375	Respiratory system	negative	negative	negative	negative	Positive	Positive
#N/A	1498	Mbam0985	M abscessus subsp massiliense	156	GCA_900139655	Respiratory system	negative	negative	negative	negative	Positive	Positive
#N/A	1499	Mbam0986	M abscessus subsp massiliense	158	GCA_900130385	Respiratory system	negative	negative	negative	negative	Positive	Positive
#N/A	1174	Mbam0987	M abscessus subsp massiliense	164	GCA_900130435	Respiratory system	negative	negative	negative	negative	Positive	Positive
#N/A	1097	Mbam0988	M abscessus subsp massiliense	168	GCA_900130285	Respiratory system	negative	negative	negative	negative	Positive	Positive
#N/A	1099	Mbam0989	M abscessus subsp massiliense	169	GCA_900130465	Respiratory system	negative	negative	negative	negative	Positive	Positive
#N/A	1151	Mbam0990	M abscessus subsp massiliense	177	GCA_900130535	Respiratory system	negative	negative	negative	negative	Positive	Positive
#N/A	1152	Mbam0991	M abscessus subsp massiliense	178	GCA_900130665	Respiratory system	negative	negative	negative	negative	Positive	Positive
#N/A	1153	Mbam0992	M abscessus subsp massiliense	179	GCA_900130675	Respiratory system	negative	negative	negative	negative	Positive	Positive
#N/A	1209	Mbam0993	M abscessus subsp massiliense	182	GCA_900130575	Respiratory system	negative	negative	negative	negative	Positive	Positive
#N/A	1201	Mbam0994	M abscessus subsp massiliense	183	GCA_002800135	sputum	negative	negative	negative	negative	Positive	Positive
#N/A	1142	Mbam0995	M abscessus subsp massiliense	184	GCA_900130585	Respiratory system	negative	negative	negative	negative	Positive	Positive
#N/A	1105	Mbam0997	M abscessus subsp massiliense	187	GCA_900130635	Respiratory system	negative	negative	negative	negative	Positive	Positive
#N/A	1136	Mbam0999	M abscessus subsp massiliense	193	GCA_900130755	Respiratory system	negative	negative	negative	negative	Positive	Positive
#N/A	1203	Mbam1001	M abscessus subsp massiliense	198	GCA_900130775	Respiratory system	negative	negative	negative	negative	Positive	Positive

#N/A	1140	Mbam1002	M abscessus subsp massiliense	200	GCA_900130795	Respiratory system	negative	negative	negative	negative	Positive	Positive
#N/A	1141	Mbam1003	M abscessus subsp massiliense	201	GCA_900130805	Respiratory system	negative	negative	negative	negative	Positive	Positive
#N/A	1145	Mbam1004	M abscessus subsp massiliense	203	GCA_900130825	Respiratory system	negative	negative	negative	negative	Positive	Positive
#N/A	1138	Mbam1005	M abscessus subsp massiliense	204	GCA_900130835	Respiratory system	negative	negative	negative	negative	Positive	Positive
#N/A	1202	Mbam1006	M abscessus subsp massiliense	206	GCA_002801845	lavage fluid	negative	negative	negative	negative	Positive	Positive
#N/A	1143	Mbam1007	M abscessus subsp massiliense	207	GCA_900130865	Respiratory system	negative	negative	negative	negative	Positive	Positive
#N/A	1137	Mbam1008	M abscessus subsp massiliense	208	GCA_900130855	Respiratory system	negative	negative	negative	negative	Positive	Positive
#N/A	1175	Mbam1009	M abscessus subsp massiliense	211	GCA_900130885	Respiratory system	negative	negative	negative	negative	Positive	Positive
#N/A	1144	Mbam1010	M abscessus subsp massiliense	213	GCA_900130905	Respiratory system	negative	negative	negative	negative	Positive	Positive
#N/A	1139	Mbam1011	M abscessus subsp massiliense	219	GCA_900130945	Respiratory system	negative	negative	negative	negative	Positive	Positive
#N/A	1157	Mbam1012	M abscessus subsp massiliense	220	GCA_900131065	Respiratory system	negative	negative	negative	negative	Positive	Positive
#N/A	1158	Mbam1013	M abscessus subsp massiliense	236	GCA_900131205	Respiratory system	negative	negative	negative	negative	Positive	Positive
#N/A	1173	Mbam1015	M abscessus subsp massiliense	238	GCA_900131235	Respiratory system	negative	negative	negative	negative	Positive	Positive
#N/A	1502	Mbam1016	M abscessus subsp massiliense	241	GCA_900131265	Respiratory system	negative	negative	negative	negative	Positive	Positive
#N/A	1159	Mbam1017	M abscessus subsp massiliense	243	GCA_900140365	Respiratory system	negative	negative	negative	negative	Positive	Positive
#N/A	1503	Mbam1018	M abscessus subsp massiliense	246	GCA_900131305	Respiratory system	negative	negative	negative	negative	Positive	Positive
#N/A	1146	Mbam1020	M abscessus subsp massiliense	249	GCA_900131325	Respiratory system	negative	negative	negative	negative	Positive	Positive
#N/A	1150	Mbam1021	M abscessus subsp massiliense	256	GCA_900131385	Respiratory system	negative	negative	negative	negative	Positive	Positive
#N/A	1154	Mbam1022	M abscessus subsp massiliense	257	GCA_900131425	Respiratory system	negative	negative	negative	negative	Positive	Positive
#N/A	1170	Mbam1023	M abscessus subsp massiliense	259	GCA_900140385	Respiratory system	negative	negative	negative	negative	Positive	Positive
#N/A	1381	Mbam1024	M abscessus subsp massiliense	260	GCA_002800125	lavage fluid	negative	negative	negative	negative	Positive	Positive
#N/A	1160	Mbam1025	M abscessus subsp massiliense	260	GCA_900131735	Respiratory system	negative	negative	negative	negative	Positive	Positive
#N/A	1161	Mbam1026	M abscessus subsp massiliense	262	GCA_900131755	Respiratory system	negative	negative	negative	negative	Positive	Positive
#N/A	1177	Mbam1027	M abscessus subsp massiliense	271	GCA_900131585	Respiratory system	negative	negative	negative	negative	Positive	Positive
#N/A	1162	Mbam1028	M abscessus subsp massiliense	274	GCA_900131405	Respiratory system	negative	negative	negative	negative	Positive	Positive
#N/A	1169	Mbam1029	M abscessus subsp massiliense	275	GCA_900131635	Respiratory system	negative	negative	negative	negative	Positive	Positive
#N/A	1163	Mbam1030	M abscessus subsp massiliense	276	GCA_900131615	Respiratory system	negative	negative	negative	negative	Positive	Positive
#N/A	1168	Mbam1031	M abscessus subsp massiliense	278	GCA_900131655	Respiratory system	negative	negative	negative	negative	Positive	Positive
#N/A	1148	Mbam1032	M abscessus subsp massiliense	282	GCA_900131685	Respiratory system	negative	negative	negative	negative	Positive	Positive
#N/A	1149	Mbam1033	M abscessus subsp massiliense	284	GCA_900131695	Respiratory system	negative	negative	negative	negative	Positive	Positive
#N/A	1530	Mbam1034	M abscessus subsp massiliense	287	GCA_002800085	lavage fluid	negative	negative	negative	negative	Positive	Positive
#N/A	1176	Mbam1035	M abscessus subsp massiliense	287	GCA_900131725	Respiratory system	negative	negative	negative	negative	Positive	Positive
#N/A	1147	Mbam1036	M abscessus subsp massiliense	289	GCA_900131895	Respiratory system	negative	negative	negative	negative	Positive	Positive
#N/A	1171	Mbam1037	M abscessus subsp massiliense	290	GCA_900131945	Respiratory system	negative	negative	negative	negative	Positive	Positive
#N/A	1172	Mbam1038	M abscessus subsp massiliense	292	GCA_900131905	Respiratory system	negative	negative	negative	negative	Positive	Positive
#N/A	1436	Mbam1039	M abscessus subsp massiliense	294	GCA_900131935	Respiratory system	negative	negative	negative	negative	Positive	Positive
#N/A	1088	Mbam1040	M abscessus subsp massiliense	300	GCA_900131995	Respiratory system	negative	negative	negative	negative	Positive	Positive
#N/A	1164	Mbam1043	M abscessus subsp massiliense	306	GCA_900132065	Respiratory system	negative	negative	negative	negative	Positive	Positive
#N/A	1106	Mbam1045	M abscessus subsp massiliense	308	GCA_900132085	Respiratory system	negative	negative	negative	negative	Positive	Positive
#N/A	1081	Mbam1048	M abscessus subsp massiliense	324	GCA_900132375	Respiratory system	negative	negative	negative	negative	Positive	Positive
#N/A	1082	Mbam1049	M abscessus subsp massiliense	325	GCA_900132395	Respiratory system	negative	negative	negative	negative	Positive	Positive
#N/A	1535	Mbam1053	M abscessus subsp massiliense	347	GCA_900132795	Respiratory system	negative	negative	negative	negative	Positive	Positive
#N/A	1383	Mbam1054	M abscessus subsp massiliense	356	GCA_900132695	Respiratory system	negative	negative	negative	negative	Positive	Positive
#N/A	1207	Mbam1055	M abscessus subsp massiliense	359	GCA_900132705	Respiratory system	negative	negative	negative	negative	Positive	Positive
#N/A	1208	Mbam1056	M abscessus subsp massiliense	360	GCA_900132725	Respiratory system	negative	negative	negative	negative	Positive	Positive
#N/A	1437	Mbam1057	M abscessus subsp massiliense	368	GCA_900133235	Respiratory system	negative	negative	negative	negative	Positive	Positive
#N/A	1215	Mbam1058	M abscessus subsp massiliense	392	GCA_900133865	Respiratory system	negative	negative	negative	negative	Positive	Positive
#N/A	1216	Mbam1059	M abscessus subsp massiliense	396	GCA_900134075	Respiratory system	negative	negative	negative	negative	Positive	Positive
#N/A	1364	Mbam1061	M abscessus subsp massiliense	428	GCA_900134885	Respiratory system	negative	negative	negative	negative	Positive	Positive
#N/A	1533	Mbam1063	M abscessus subsp massiliense	443	GCA_900134765	Respiratory system	negative	negative	negative	negative	Positive	Positive
#N/A	1366	Mbam1066	M abscessus subsp massiliense	471	GCA_900138805	Respiratory system	negative	negative	negative	negative	Positive	Positive
#N/A	1457	Mbam1068	M abscessus subsp massiliense	476	GCA_900138845	Respiratory system	negative	negative	negative	negative	Positive	Positive
#N/A	1217	Mbam1070	M abscessus subsp massiliense	482	GCA_900138905	Respiratory system	negative	negative	negative	negative	Positive	Positive

#N/A	1450	Mbam1072	M abscessus subsp massiliense	485	GCA_900138955	Respiratory system	negative	negative	negative	negative	Positive	Positive
#N/A	1522	Mbam1075	M abscessus subsp massiliense	496	GCA_900136525	Respiratory system	negative	negative	negative	negative	Positive	Positive
#N/A	1534	Mbam1077	M abscessus subsp massiliense	519	GCA_900139715	Respiratory system	negative	negative	negative	negative	Positive	Positive
#N/A	1210	Mbam1078	M abscessus subsp massiliense	524	GCA_900140155	Respiratory system	negative	negative	negative	negative	Positive	Positive
#N/A	1211	Mbam1079	M abscessus subsp massiliense	525	GCA_900139005	Respiratory system	negative	negative	negative	negative	Positive	Positive
#N/A	1547	Mbam1080	M abscessus subsp massiliense	526	GCA_900139915	Respiratory system	negative	negative	negative	negative	Positive	Positive
#N/A	1330	Mbam1082	M abscessus subsp massiliense	538	GCA_900140445	Respiratory system	negative	negative	negative	negative	Positive	Positive
#N/A	1458	Mbam1083	M abscessus subsp massiliense	586	GCA_900140495	Respiratory system	negative	negative	negative	negative	Positive	Positive
#N/A	1465	Mbam1084	M abscessus subsp massiliense	598	GCA_900140585	Respiratory system	negative	negative	negative	negative	Positive	Positive
#N/A	1468	Mbam1085	M abscessus subsp massiliense	603	GCA_900140635	Respiratory system	Positive	negative	negative	negative	Positive	Positive
#N/A	1213	Mbam1086	M abscessus subsp massiliense	609	GCA_900139095	Respiratory system	negative	negative	negative	negative	Positive	Positive
#N/A	1548	Mbam1088	M abscessus subsp massiliense	618	GCA_900140755	Respiratory system	negative	negative	negative	negative	Positive	Positive
#N/A	1455	Mbam1089	M abscessus subsp massiliense	620	GCA_900140765	Respiratory system	negative	negative	negative	negative	Positive	Positive
#N/A	1431	Mbam1090	M abscessus subsp massiliense	628	GCA_900140775	Respiratory system	negative	negative	negative	negative	Positive	Positive
#N/A	1449	Mbam1091	M abscessus subsp massiliense	631	GCA_900140845	Respiratory system	negative	negative	negative	negative	Positive	Positive
#N/A	1464	Mbam1092	M abscessus subsp massiliense	635	GCA_900139905	Respiratory system	negative	negative	negative	negative	Positive	Positive
#N/A	1324	Mbam1093	M abscessus subsp massiliense	637	GCA_900140865	Respiratory system	negative	negative	negative	negative	Positive	Positive
#N/A	1463	Mbam1094	M abscessus subsp massiliense	651	GCA_900139775	Respiratory system	negative	negative	negative	negative	Positive	Positive
#N/A	1462	Mbam1095	M abscessus subsp massiliense	659	GCA_900139145	Respiratory system	negative	negative	negative	negative	Positive	Positive
#N/A	1518	Mbam1096	M abscessus subsp massiliense	663	GCA_900141045	Respiratory system	negative	negative	negative	negative	Positive	Positive
#N/A	1199	Mbam1097	M abscessus subsp massiliense	666	GCA_900141015	Respiratory system	negative	negative	negative	negative	Positive	Positive
#N/A	1200	Mbam1100	M abscessus subsp massiliense	682	GCA_900139225	Respiratory system	negative	negative	negative	negative	Positive	Positive
#N/A	1507	Mbam1102	M abscessus subsp massiliense	694	GCA_900141185	Respiratory system	negative	negative	negative	negative	Positive	Positive
#N/A	1448	Mbam1103	M abscessus subsp massiliense	704	GCA_900141245	Respiratory system	negative	negative	negative	negative	Positive	Positive
#N/A	1519	Mbam1104	M abscessus subsp massiliense	705	GCA_900141225	Respiratory system	negative	negative	negative	negative	Positive	Positive
#N/A	1428	Mbam1105	M abscessus subsp massiliense	707	GCA_900141275	Respiratory system	negative	negative	negative	negative	Positive	Positive
#N/A	1026	Mbam1106	M abscessus subsp massiliense	726	GCA_900140305	Respiratory system	negative	negative	negative	negative	Positive	Positive
#N/A	1027	Mbam1107	M abscessus subsp massiliense	727	GCA_900140295	Respiratory system	negative	negative	negative	negative	Positive	Positive
#N/A	1028	Mbam1108	M abscessus subsp massiliense	728	GCA_900140225	Respiratory system	negative	negative	negative	negative	Positive	Positive
#N/A	1365	Mbam1109	M abscessus subsp massiliense	730	GCA_900139835	Respiratory system	negative	negative	negative	negative	Positive	Positive
#N/A	1108	Mbam1110	M abscessus subsp massiliense	755	GCA_900130545	Respiratory system	negative	negative	negative	negative	Positive	Positive
#N/A	1540	Mbam1111	M abscessus subsp massiliense	784	GCA_900135875	Respiratory system	negative	negative	negative	negative	Positive	Positive
#N/A	1538	Mbam1112	M abscessus subsp massiliense	785	GCA_900136965	Respiratory system	negative	negative	negative	negative	Positive	Positive
#N/A	1539	Mbam1113	M abscessus subsp massiliense	786	GCA_900137095	Respiratory system	negative	negative	negative	negative	Positive	Positive
#N/A	1454	Mbam1114	M abscessus subsp massiliense	787	GCA_900141355	Respiratory system	negative	negative	negative	negative	Positive	Positive
#N/A	1329	Mbam1115	M abscessus subsp massiliense	788	GCA_900132185	Respiratory system	negative	negative	negative	negative	Positive	Positive
#N/A	1328	Mbam1116	M abscessus subsp massiliense	789	GCA_900132195	Respiratory system	negative	negative	negative	negative	Positive	Positive
#N/A	1325	Mbam1117	M abscessus subsp massiliense	790	GCA_900132495	Respiratory system	negative	negative	negative	negative	Positive	Positive
#N/A	1326	Mbam1118	M abscessus subsp massiliense	791	GCA_900135695	Respiratory system	negative	negative	negative	negative	Positive	Positive
#N/A	1254	Mbam1119	M abscessus subsp massiliense	792	GCA_900130715	Respiratory system	negative	negative	negative	negative	Positive	Positive
#N/A	1253	Mbam1120	M abscessus subsp massiliense	794	GCA_900131025	Respiratory system	negative	negative	negative	negative	Positive	Positive
#N/A	1247	Mbam1121	M abscessus subsp massiliense	795	GCA_900131815	Respiratory system	negative	negative	negative	negative	Positive	Positive
#N/A	1248	Mbam1122	M abscessus subsp massiliense	797	GCA_900132925	Respiratory system	negative	negative	negative	negative	Positive	Positive
#N/A	1246	Mbam1123	M abscessus subsp massiliense	798	GCA_900132895	Respiratory system	negative	negative	negative	negative	Positive	Positive
#N/A	1249	Mbam1124	M abscessus subsp massiliense	799	GCA_900133485	Respiratory system	negative	negative	negative	negative	Positive	Positive
#N/A	1426	Mbam1125	M abscessus subsp massiliense	812	GCA_900135755	Respiratory system	negative	negative	negative	negative	Positive	Positive
#N/A	1523	Mbam1126	M abscessus subsp massiliense	813	GCA_900137025	Respiratory system	negative	negative	negative	negative	Positive	Positive
#N/A	1408	Mbam1127	M abscessus subsp massiliense	825	GCA_900135735	Respiratory system	negative	negative	negative	negative	Positive	Positive
#N/A	1386	Mbam1128	M abscessus subsp massiliense	826	GCA_900135745	Respiratory system	negative	negative	negative	negative	Positive	Positive
#N/A	1377	Mbam1129	M abscessus subsp massiliense	837	GCA_900131515	Respiratory system	negative	negative	negative	negative	Positive	Positive
#N/A	1374	Mbam1130	M abscessus subsp massiliense	838	GCA_900131545	Respiratory system	negative	negative	negative	negative	Positive	Positive
#N/A	1375	Mbam1131	M abscessus subsp massiliense	839	GCA_900131865	Respiratory system	negative	negative	negative	negative	Positive	Positive
#N/A	1376	Mbam1132	M abscessus subsp massiliense	840	GCA_900132275	Respiratory system	negative	negative	negative	negative	Positive	Positive

#N/A	1109	Mbam1133	M abscessus subsp massiliense	842	GCA_900138085	Respiratory system	negative	negative	negative	negative	Positive	Positive
#N/A	1101	Mbam1134	M abscessus subsp massiliense	843	GCA_900137985	Respiratory system	negative	negative	negative	negative	Positive	Positive
#N/A	1341	Mbam1135	M abscessus subsp massiliense	856	GCA_900135835	Respiratory system	negative	negative	negative	negative	Positive	Positive
#N/A	1425	Mbam1136	M abscessus subsp massiliense	860	GCA_900131505	Respiratory system	negative	negative	negative	negative	Positive	Positive
#N/A	1413	Mbam1137	M abscessus subsp massiliense	861	GCA_900131855	Respiratory system	negative	negative	negative	negative	Positive	Positive
#N/A	1414	Mbam1138	M abscessus subsp massiliense	862	GCA_900131875	Respiratory system	negative	negative	negative	negative	Positive	Positive
#N/A	1415	Mbam1139	M abscessus subsp massiliense	863	GCA_900132215	Respiratory system	negative	negative	negative	negative	Positive	Positive
#N/A	1416	Mbam1140	M abscessus subsp massiliense	864	GCA_900132235	Respiratory system	negative	negative	negative	negative	Positive	Positive
#N/A	1417	Mbam1141	M abscessus subsp massiliense	865	GCA_900132225	Respiratory system	negative	negative	negative	negative	Positive	Positive
#N/A	1418	Mbam1142	M abscessus subsp massiliense	866	GCA_900132245	Respiratory system	negative	negative	negative	negative	Positive	Positive
#N/A	1419	Mbam1143	M abscessus subsp massiliense	867	GCA_900132535	Respiratory system	negative	negative	negative	negative	Positive	Positive
#N/A	1420	Mbam1144	M abscessus subsp massiliense	868	GCA_900133015	Respiratory system	negative	negative	negative	negative	Positive	Positive
#N/A	1421	Mbam1145	M abscessus subsp massiliense	869	GCA_900133585	Respiratory system	negative	negative	negative	negative	Positive	Positive
#N/A	1422	Mbam1146	M abscessus subsp massiliense	870	GCA_900134315	Respiratory system	negative	negative	negative	negative	Positive	Positive
#N/A	1423	Mbam1147	M abscessus subsp massiliense	871	GCA_900134325	Respiratory system	negative	negative	negative	negative	Positive	Positive
#N/A	1424	Mbam1148	M abscessus subsp massiliense	872	GCA_900137105	Respiratory system	negative	negative	negative	negative	Positive	Positive
#N/A	1110	Mbam1149	M abscessus subsp massiliense	873	GCA_900134235	Respiratory system	negative	negative	negative	negative	Positive	Positive
#N/A	1334	Mbam1150	M abscessus subsp massiliense	882	GCA_900131525	Respiratory system	negative	negative	negative	negative	Positive	Positive
#N/A	1331	Mbam1151	M abscessus subsp massiliense	883	GCA_900131535	Respiratory system	negative	negative	negative	negative	Positive	Positive
#N/A	1340	Mbam1152	M abscessus subsp massiliense	884	GCA_900132265	Respiratory system	negative	negative	negative	negative	Positive	Positive
#N/A	1335	Mbam1153	M abscessus subsp massiliense	885	GCA_900133025	Respiratory system	negative	negative	negative	negative	Positive	Positive
#N/A	1337	Mbam1154	M abscessus subsp massiliense	886	GCA_900133595	Respiratory system	negative	negative	negative	negative	Positive	Positive
#N/A	1256	Mbam1155	M abscessus subsp massiliense	899	GCA_900141425	Respiratory system	negative	negative	negative	negative	Positive	Positive
#N/A	1488	Mbam1156	M abscessus subsp massiliense	911	GCA_900133065	Respiratory system	negative	negative	negative	negative	Positive	Positive
#N/A	1544	Mbam1157	M abscessus subsp massiliense	919	GCA_900132975	Respiratory system	negative	negative	negative	negative	Positive	Positive
#N/A	1245	Mbam1158	M abscessus subsp massiliense	922	GCA_900133185	Respiratory system	negative	negative	negative	negative	Positive	Positive
#N/A	1165	Mbam1159	M abscessus subsp massiliense	924	GCA_900133155	Respiratory system	negative	negative	negative	negative	Positive	Positive
#N/A	1244	Mbam1160	M abscessus subsp massiliense	925	GCA_900133145	Respiratory system	negative	negative	negative	negative	Positive	Positive
#N/A	1545	Mbam1161	M abscessus subsp massiliense	926	GCA_900133165	Respiratory system	negative	negative	negative	negative	Positive	Positive
#N/A	1238	Mbam1162	M abscessus subsp massiliense	928	GCA_900133825	Respiratory system	negative	negative	negative	negative	Positive	Positive
#N/A	1352	Mbam1163	M abscessus subsp massiliense	932	GCA_900133605	Respiratory system	negative	negative	negative	negative	Positive	Positive
#N/A	1087	Mbam1164	M abscessus subsp massiliense	938	GCA_900133745	Respiratory system	negative	negative	negative	negative	Positive	Positive
#N/A	1084	Mbam1165	M abscessus subsp massiliense	939	GCA_900133725	Respiratory system	negative	negative	negative	negative	Positive	Positive
#N/A	1093	Mbam1166	M abscessus subsp massiliense	940	GCA_900133695	Respiratory system	negative	negative	negative	negative	Positive	Positive
#N/A	1473	Mbam1167	M abscessus subsp massiliense	943	GCA_900133675	Respiratory system	negative	negative	negative	negative	Positive	Positive
#N/A	1103	Mbam1168	M abscessus subsp massiliense	945	GCA_900133735	Respiratory system	negative	negative	negative	negative	Positive	Positive
#N/A	1179	Mbam1169	M abscessus subsp massiliense	947	GCA_900133765	Respiratory system	negative	negative	negative	negative	Positive	Positive
#N/A	1092	Mbam1170	M abscessus subsp massiliense	948	GCA_900133755	Respiratory system	negative	negative	negative	negative	Positive	Positive
#N/A	1180	Mbam1171	M abscessus subsp massiliense	950	GCA_900133615	Respiratory system	negative	negative	negative	negative	Positive	Positive
#N/A	1239	Mbam1172	M abscessus subsp massiliense	953	GCA_900134405	Respiratory system	negative	negative	negative	negative	Positive	Positive
#N/A	1181	Mbam1173	M abscessus subsp massiliense	958	GCA_900134445	Respiratory system	negative	negative	negative	negative	Positive	Positive
#N/A	1477	Mbam1174	M abscessus subsp massiliense	960	GCA_900134475	Respiratory system	negative	negative	negative	negative	Positive	Positive
#N/A	1475	Mbam1175	M abscessus subsp massiliense	967	GCA_900134495	Respiratory system	negative	negative	negative	negative	Positive	Positive
#N/A	1243	Mbam1176	M abscessus subsp massiliense	969	GCA_900134545	Respiratory system	negative	negative	negative	negative	Positive	Positive
#N/A	1104	Mbam1177	M abscessus subsp massiliense	971	GCA_900134575	Respiratory system	negative	negative	negative	negative	Positive	Positive
#N/A	1380	Mbam1178	M abscessus subsp massiliense	974	GCA_900134565	Respiratory system	negative	negative	negative	negative	Positive	Positive
#N/A	1182	Mbam1179	M abscessus subsp massiliense	981	GCA_900136005	Respiratory system	negative	negative	negative	negative	Positive	Positive
#N/A	1186	Mbam1180	M abscessus subsp massiliense	983	GCA_900136025	Respiratory system	negative	negative	negative	negative	Positive	Positive
#N/A	1487	Mbam1181	M abscessus subsp massiliense	995	GCA_900136125	Respiratory system	negative	negative	negative	negative	Positive	Positive
#N/A	1240	Mbam1182	M abscessus subsp massiliense	999	GCA_900136175	Respiratory system	negative	negative	negative	negative	Positive	Positive
#N/A	1321	Mbam1183	M abscessus subsp massiliense	1001	GCA_900136195	Respiratory system	negative	negative	negative	negative	Positive	Positive
#N/A	1314	Mbam1184	M abscessus subsp massiliense	1004	GCA_900135955	Respiratory system	negative	negative	negative	negative	Positive	Positive
#N/A	1320	Mbam1185	M abscessus subsp massiliense	1006	GCA_900135965	Respiratory system	negative	negative	negative	negative	Positive	Positive

#N/A	1241	Mbam1186	M abscessus subsp massiliense	1007	GCA_900136225	Respiratory system	negative	negative	negative	negative	Positive	Positive
#N/A	1319	Mbam1187	M abscessus subsp massiliense	1012	GCA_900136275	Respiratory system	negative	negative	negative	negative	Positive	Positive
#N/A	1486	Mbam1188	M abscessus subsp massiliense	1014	GCA_900136315	Respiratory system	negative	negative	negative	negative	Positive	Positive
#N/A	1411	Mbam1189	M abscessus subsp massiliense	1015	GCA_900136295	Respiratory system	negative	negative	negative	negative	Positive	Positive
#N/A	1315	Mbam1190	M abscessus subsp massiliense	1017	GCA_900136325	Respiratory system	negative	negative	negative	negative	Positive	Positive
#N/A	1410	Mbam1191	M abscessus subsp massiliense	1018	GCA_900136375	Respiratory system	negative	negative	negative	negative	Positive	Positive
#N/A	1316	Mbam1192	M abscessus subsp massiliense	1021	GCA_900137295	Respiratory system	negative	negative	negative	negative	Positive	Positive
#N/A	1183	Mbam1193	M abscessus subsp massiliense	1032	GCA_900137725	Respiratory system	negative	negative	negative	negative	Positive	Positive
#N/A	1317	Mbam1194	M abscessus subsp massiliense	1035	GCA_900137945	Respiratory system	negative	negative	negative	negative	Positive	Positive
#N/A	1379	Mbam1195	M abscessus subsp massiliense	1038	GCA_900137335	Respiratory system	negative	negative	negative	negative	Positive	Positive
#N/A	1185	Mbam1196	M abscessus subsp massiliense	1049	GCA_900137475	Respiratory system	negative	negative	negative	negative	Positive	Positive
#N/A	1318	Mbam1197	M abscessus subsp massiliense	1052	GCA_900137485	Respiratory system	negative	negative	negative	negative	Positive	Positive
#N/A	1485	Mbam1198	M abscessus subsp massiliense	1057	GCA_900137555	Respiratory system	negative	negative	negative	negative	Positive	Positive
#N/A	1257	Mbam1199	M abscessus subsp massiliense	1066	GCA_900137625	Respiratory system	negative	negative	negative	negative	Positive	Positive
#N/A	1476	Mbam1200	M abscessus subsp massiliense	1072	GCA_900137695	Respiratory system	negative	negative	negative	negative	Positive	Positive
#N/A	1336	Mbam1201	M abscessus subsp massiliense	1091	GCA_900133195	Respiratory system	negative	negative	negative	negative	Positive	Positive
#N/A	1339	Mbam1202	M abscessus subsp massiliense	1092	GCA_900133875	Respiratory system	negative	negative	negative	negative	Positive	Positive
#N/A	1338	Mbam1203	M abscessus subsp massiliense	1093	GCA_900134655	Respiratory system	negative	negative	negative	negative	Positive	Positive
#N/A	1333	Mbam1204	M abscessus subsp massiliense	1094	GCA_900134635	Respiratory system	negative	negative	negative	negative	Positive	Positive
#N/A	1327	Mbam1205	M abscessus subsp massiliense	1113	GCA_900135985	Respiratory system	negative	negative	negative	negative	Positive	Positive
#N/A	1427	Mbam1206	M abscessus subsp massiliense	1115	GCA_900137835	Respiratory system	negative	negative	negative	negative	Positive	Positive
#N/A	1332	Mbam1207	M abscessus subsp massiliense	1117	GCA_900132635	Respiratory system	negative	negative	negative	negative	Positive	Positive
#N/A	1252	Mbam1208	M abscessus subsp massiliense	1118	GCA_900134685	Respiratory system	negative	negative	negative	negative	Positive	Positive
#N/A	1250	Mbam1209	M abscessus subsp massiliense	1119	GCA_900136345	Respiratory system	negative	negative	negative	negative	Positive	Positive
#N/A	1251	Mbam1210	M abscessus subsp massiliense	1120	GCA_900137875	Respiratory system	negative	negative	negative	negative	Positive	Positive
#N/A	1242	Mbam1211	M abscessus subsp massiliense	1126	GCA_900136395	Respiratory system	negative	negative	negative	negative	Positive	Positive
#N/A	1072	Mbam1212	M abscessus subsp massiliense	1127	GCA_900137245	Respiratory system	negative	negative	negative	negative	Positive	Positive
#N/A	1372	Mbam1213	M abscessus subsp massiliense	1140	GCA_900138155	Respiratory system	negative	negative	negative	negative	Positive	Positive
#N/A	1373	Mbam1214	M abscessus subsp massiliense	1141	GCA_900138165	Respiratory system	negative	negative	negative	negative	Positive	Positive
#N/A	1478	Mbam1215	M abscessus subsp massiliense	1164	GCA_900141475	Respiratory system	negative	negative	negative	negative	Positive	Positive
#N/A	1029	Mbam1216	M abscessus subsp massiliense	1166	GCA_900141515	Respiratory system	negative	negative	negative	negative	Positive	Positive
#N/A	1030	Mbam1217	M abscessus subsp massiliense	1167	GCA_900141505	Respiratory system	negative	negative	negative	negative	Positive	Positive
#N/A	1031	Mbam1218	M abscessus subsp massiliense	1168	GCA_900138135	Respiratory system	negative	negative	negative	negative	Positive	Positive
#N/A	1032	Mbam1219	M abscessus subsp massiliense	1172	GCA_900135905	Respiratory system	negative	negative	negative	negative	Positive	Positive
#N/A	1069	Mbam1220	M abscessus subsp massiliense	1173	GCA_900137185	Respiratory system	negative	negative	negative	negative	Positive	Positive
#N/A	1033	Mbam1221	M abscessus subsp massiliense	1174	GCA_900135915	Respiratory system	negative	negative	negative	negative	Positive	Positive
#N/A	1034	Mbam1222	M abscessus subsp massiliense	1177	GCA_900138505	Respiratory system	negative	negative	negative	negative	Positive	Positive
#N/A	1322	Mbam1224	M abscessus subsp massiliense	1398	GCA_002801825	sputum	negative	negative	negative	negative	Positive	Positive
#N/A	1298	Mbam1227	M abscessus subsp massiliense	3377	GCA_002800065	sputum	negative	negative	negative	negative	Positive	Positive
#N/A	1350	Mbam1228	M abscessus subsp massiliense	3742	GCA_002800045	sputum	negative	negative	negative	negative	Positive	Positive
#N/A	1035	Mbam1232	M abscessus subsp massiliense	1168_1	GCA_900138295	Respiratory system	negative	negative	negative	negative	Positive	Positive
#N/A	1036	Mbam1233	M abscessus subsp massiliense	1168_10	GCA_900138375	Respiratory system	negative	negative	negative	negative	Positive	Positive
#N/A	1037	Mbam1234	M abscessus subsp massiliense	1168_11	GCA_900138395	Respiratory system	negative	negative	negative	negative	Positive	Positive
#N/A	1038	Mbam1235	M abscessus subsp massiliense	1168_12	GCA_900138425	Respiratory system	negative	negative	negative	negative	Positive	Positive
#N/A	1039	Mbam1236	M abscessus subsp massiliense	1168_13	GCA_900138405	Respiratory system	negative	negative	negative	negative	Positive	Positive
#N/A	1040	Mbam1237	M abscessus subsp massiliense	1168_14	GCA_900138415	Respiratory system	negative	negative	negative	negative	Positive	Positive
#N/A	1041	Mbam1238	M abscessus subsp massiliense	1168_15	GCA_900138435	Respiratory system	negative	negative	negative	negative	Positive	Positive
#N/A	1042	Mbam1239	M abscessus subsp massiliense	1168_16	GCA_900138465	Respiratory system	negative	negative	negative	negative	Positive	Positive
#N/A	1043	Mbam1240	M abscessus subsp massiliense	1168_17	GCA_900138455	Respiratory system	negative	negative	negative	negative	Positive	Positive
#N/A	1044	Mbam1241	M abscessus subsp massiliense	1168_18	GCA_900138445	Respiratory system	negative	negative	negative	negative	Positive	Positive
#N/A	1045	Mbam1242	M abscessus subsp massiliense	1168_19	GCA_900138475	Respiratory system	negative	negative	negative	negative	Positive	Positive
#N/A	1046	Mbam1243	M abscessus subsp massiliense	1168_2	GCA_900138305	Respiratory system	negative	negative	negative	negative	Positive	Positive
#N/A	1047	Mbam1244	M abscessus subsp massiliense	1168_20	GCA_900138485	Respiratory system	negative	negative	negative	negative	Positive	Positive

#N/A	1048	Mbam1245	M abscessus subsp massiliense	1168_3	GCA_900138325	Respiratory system	negative	negative	negative	negative	Positive	Positive
#N/A	1049	Mbam1246	M abscessus subsp massiliense	1168_4	GCA_900138315	Respiratory system	negative	negative	negative	negative	Positive	Positive
#N/A	1050	Mbam1247	M abscessus subsp massiliense	1168_5	GCA_900138345	Respiratory system	negative	negative	negative	negative	Positive	Positive
#N/A	1051	Mbam1248	M abscessus subsp massiliense	1168_6	GCA_900138335	Respiratory system	negative	negative	negative	negative	Positive	Positive
#N/A	1052	Mbam1249	M abscessus subsp massiliense	1168_7	GCA_900138355	Respiratory system	negative	negative	negative	negative	Positive	Positive
#N/A	1064	Mbam1250	M abscessus subsp massiliense	1168_8	GCA_900138385	Respiratory system	negative	negative	negative	negative	Positive	Positive
#N/A	1053	Mbam1251	M abscessus subsp massiliense	1168_9	GCA_900138365	Respiratory system	negative	negative	negative	negative	Positive	Positive
#N/A	1272	Mbam1252	M abscessus subsp massiliense	3275STDY5961976	GCA_900169205	skin and soft tissue	negative	negative	negative	negative	Positive	Positive
#N/A	1345	Mbam1254	M abscessus subsp massiliense	A173	GCA_002799965	lavage fluid	negative	negative	negative	negative	Positive	Positive
#N/A	1346	Mbam1255	M abscessus subsp massiliense	A174	GCA_002801795	sputum	negative	negative	negative	negative	Positive	Positive
#N/A	1255	Mbam1256	M abscessus subsp massiliense	A186	GCA_002799945	sputum	negative	negative	negative	negative	Positive	Positive
#N/A	1536	Mbam1257	M abscessus subsp massiliense	a205	GCA_002799915	sputum	negative	negative	negative	negative	Positive	Positive
#N/A	1356	Mbam1258	M abscessus subsp massiliense	A222	GCA_002799905	sputum	negative	negative	negative	negative	Positive	Positive
#N/A	1378	Mbam1261	M abscessus subsp massiliense	A254	GCA_002801785	sputum	Positive	negative	negative	negative	Positive	Positive
#N/A	1312	Mbam1262	M abscessus subsp massiliense	A267	GCA_002799845	lavage fluid	negative	negative	negative	negative	Positive	Positive
#N/A	1524	Mbam1263	M abscessus subsp massiliense	A268	GCA_002801725	sputum	negative	negative	negative	negative	Positive	Positive
#N/A	1294	Mbam1266	M abscessus subsp massiliense	A323	GCA_002799805	sputum	negative	negative	negative	negative	Positive	Positive
#N/A	1295	Mbam1267	M abscessus subsp massiliense	A365	GCA_002799765	sputum	negative	negative	negative	negative	Positive	Positive
#N/A	1353	Mbam1268	M abscessus subsp massiliense	A39	GCA_002799695	sputum	negative	negative	negative	negative	Positive	Positive
#N/A	1349	Mbam1269	M abscessus subsp massiliense	A399	GCA_002799685	sputum	negative	negative	negative	negative	Positive	Positive
#N/A	1347	Mbam1270	M abscessus subsp massiliense	A425	GCA_002799725	sputum	negative	negative	negative	negative	Positive	Positive
#N/A	1299	Mbam1271	M abscessus subsp massiliense	A428	GCA_002799655	sputum	negative	negative	negative	negative	Positive	Positive
#N/A	1206	Mbam1273	M abscessus subsp massiliense	A484	GCA_002799715	sputum	negative	negative	negative	negative	Positive	Positive
#N/A	1528	Mbam1275	M abscessus subsp massiliense	A70	GCA_002799625	sputum	negative	negative	negative	negative	Positive	Positive
#N/A	1387	Mbam1276	M abscessus subsp massiliense	aerosol_1	GCA_900138515	Respiratory system	negative	negative	negative	negative	Positive	Positive
#N/A	1388	Mbam1277	M abscessus subsp massiliense	aerosol_10	GCA_900138705	Respiratory system	negative	negative	negative	negative	Positive	Positive
#N/A	1389	Mbam1278	M abscessus subsp massiliense	aerosol_11	GCA_900138715	Respiratory system	negative	negative	negative	negative	Positive	Positive
#N/A	1405	Mbam1279	M abscessus subsp massiliense	aerosol_13	GCA_900138545	Respiratory system	negative	negative	negative	negative	Positive	Positive
#N/A	1406	Mbam1280	M abscessus subsp massiliense	aerosol_14	GCA_900138555	Respiratory system	negative	negative	negative	negative	Positive	Positive
#N/A	1390	Mbam1281	M abscessus subsp massiliense	aerosol_15	GCA_900138575	Respiratory system	negative	negative	negative	negative	Positive	Positive
#N/A	1391	Mbam1282	M abscessus subsp massiliense	aerosol_17	GCA_900138595	Respiratory system	negative	negative	negative	negative	Positive	Positive
#N/A	1404	Mbam1283	M abscessus subsp massiliense	aerosol_18	GCA_900138615	Respiratory system	negative	negative	negative	negative	Positive	Positive
#N/A	1407	Mbam1284	M abscessus subsp massiliense	aerosol_19	GCA_900138585	Respiratory system	negative	negative	negative	negative	Positive	Positive
#N/A	1392	Mbam1285	M abscessus subsp massiliense	aerosol_24	GCA_900138655	Respiratory system	negative	negative	negative	negative	Positive	Positive
#N/A	1393	Mbam1286	M abscessus subsp massiliense	aerosol_25	GCA_900138625	Respiratory system	negative	negative	negative	negative	Positive	Positive
#N/A	1394	Mbam1287	M abscessus subsp massiliense	aerosol_26	GCA_900138645	Respiratory system	negative	negative	negative	negative	Positive	Positive
#N/A	1395	Mbam1288	M abscessus subsp massiliense	aerosol_4	GCA_900138675	Respiratory system	negative	negative	negative	negative	Positive	Positive
#N/A	1396	Mbam1289	M abscessus subsp massiliense	aerosol_5	GCA_900138665	Respiratory system	negative	negative	negative	negative	Positive	Positive
#N/A	1397	Mbam1290	M abscessus subsp massiliense	aerosol_6	GCA_900138685	Respiratory system	negative	negative	negative	negative	Positive	Positive
#N/A	1398	Mbam1291	M abscessus subsp massiliense	aerosol_7	GCA_900138695	Respiratory system	negative	negative	negative	negative	Positive	Positive
#N/A	1399	Mbam1292	M abscessus subsp massiliense	aerosol_9	GCA_900138725	Respiratory system	negative	negative	negative	negative	Positive	Positive
#N/A	1400	Mbam1293	M abscessus subsp massiliense	aerosol_Bronch_1	GCA_900136405	Respiratory system	negative	negative	negative	negative	Positive	Positive
#N/A	1401	Mbam1294	M abscessus subsp massiliense	aerosol_Bronch_2	GCA_900136425	Respiratory system	negative	negative	negative	negative	Positive	Positive
#N/A	1402	Mbam1295	M abscessus subsp massiliense	aerosol_Sputum_1	GCA_900138525	Respiratory system	negative	negative	negative	negative	Positive	Positive
#N/A	1403	Mbam1296	M abscessus subsp massiliense	aerosol_Sputum_2	GCA_900138635	Respiratory system	negative	negative	negative	negative	Positive	Positive
#N/A	1054	Mbam1304	M abscessus subsp massiliense	Room_15_sink	GCA_900138535	Respiratory system	negative	negative	negative	negative	Positive	Positive
#N/A	1469	Mbam1306	M abscessus	23	GCA_001792635	respiratory	negative	negative	negative	negative	Positive	Positive
#N/A	1541	Mbam1307	M abscessus	175	GCA_003072265	sputum	negative	negative	negative	negative	negative	negative
#N/A	1184	Mbam1320	M abscessus	47J26	GCA_000231155	sputum	negative	negative	negative	negative	Positive	Positive
#N/A	1508	Mbam1329	M abscessus	5S-0422	GCA_000271065	sputum	negative	negative	negative	negative	negative	negative
#N/A	1509	Mbam1330	M abscessus	5S-0708	GCA_000271085	sputum	negative	negative	negative	negative	negative	negative
#N/A	1510	Mbam1331	M abscessus	5S-0817	GCA_000271005	lung autopsy	negative	negative	negative	negative	negative	negative
#N/A	1515	Mbam1332	M abscessus	5S-0921	GCA_000270965	lymphnode biopsy	negative	negative	negative	negative	negative	negative

#N/A	1511	Mbam1334	M abscessus	5S-1215	GCA_000271045	sputum	negative	negative	negative	negative	negative	negative
#N/A	1085	Mbam1357	M abscessus	FLAC008	GCA_001606275	Homo sapiens	negative	negative	negative	negative	Positive	Positive
#N/A	1456	Mbam1358	M abscessus	FLAC012	GCA_002142915	Homo sapiens	negative	negative	negative	negative	Positive	Positive
#N/A	1444	Mbam1366	M abscessus	FLAC040	GCA_002142855	Homo sapiens	negative	negative	negative	negative	Positive	Positive
#N/A	1451	Mbam1371	M abscessus	FLAC050	GCA_002142835	Homo sapiens	negative	negative	negative	negative	Positive	Positive
#N/A	1452	Mbam1372	M abscessus	FLAC051	GCA_002142785	Homo sapiens	negative	negative	negative	negative	Positive	Positive
#N/A	1461	Mbam1373	M abscessus	FLAC052	GCA_002142755	Homo sapiens	negative	negative	negative	negative	Positive	Positive
#N/A	1506	Mbam1375	M abscessus	FLAC054	GCA_001610675	Homo sapiens	negative	negative	negative	negative	Positive	Positive
#N/A	1445	Mbam1377	M abscessus	FLAC078	GCA_002142765	Homo sapiens	negative	negative	negative	negative	Positive	Positive
#N/A	1525	Mbam1380	M abscessus	G105	GCA_003583035	sputum	negative	negative	negative	negative	Positive	Positive
#N/A	1212	Mbam1382	M abscessus	G107	GCA_003583005	Bronchoalveolar lavage	negative	negative	negative	negative	Positive	Positive
#N/A	1308	Mbam1391	M abscessus	G116	GCA_003582855	sputum	negative	negative	negative	negative	Positive	Positive
#N/A	1367	Mbam1399	M abscessus	G126	GCA_003582745	Bronchoalveolar lavage	negative	negative	negative	negative	Positive	Positive
#N/A	1409	Mbam1404	M abscessus	G135	GCA_003582655	sputum	negative	negative	negative	negative	Positive	Positive
#N/A	1542	Mbam1405	M abscessus	G137	GCA_003582645	sputum	negative	negative	negative	negative	Positive	Positive
#N/A	1167	Mbam1408	M abscessus	G141	GCA_003076855	sputum	negative	negative	negative	negative	Positive	Positive
#N/A	1237	Mbam1412	M abscessus	G147	GCA_003072155	sputum	negative	negative	negative	negative	Positive	Positive
#N/A	1197	Mbam1416	M abscessus	G153	GCA_003076875	sputum	negative	negative	negative	negative	Positive	Positive
#N/A	1296	Mbam1418	M abscessus	G157	GCA_003582475	Bronchoalveolar lavage	negative	negative	negative	negative	Positive	Positive
#N/A	1412	Mbam1427	M abscessus	G173	GCA_003582375	sputum	negative	negative	negative	negative	Positive	Positive
#N/A	1306	Mbam1438	M abscessus	G188	GCA_003582175	sputum	negative	negative	negative	negative	Positive	Positive
#N/A	1307	Mbam1439	M abscessus	G190	GCA_003582165	sputum	negative	negative	negative	negative	Positive	Positive
#N/A	1532	Mbam1448	M abscessus	G203	GCA_003071905	sputum	negative	negative	negative	negative	Positive	Positive
#N/A	1543	Mbam1451	M abscessus	G207	GCA_003072295	sputum	negative	negative	negative	negative	Positive	Positive
#N/A	1204	Mbam1458	M abscessus	G220	GCA_003076795	sputum	negative	negative	negative	negative	Positive	Positive
#N/A	1348	Mbam1462	M abscessus	G75	GCA_003581825	sputum	negative	negative	negative	negative	Positive	Positive
#N/A	1300	Mbam1464	M abscessus	G77	GCA_003072165	sputum	negative	negative	negative	negative	Positive	Positive
#N/A	1205	Mbam1469	M abscessus	G88	GCA_003072345	Bronchoalveolar lavage	negative	negative	negative	negative	Positive	Positive
#N/A	1089	Mbam1474	M abscessus	G95	GCA_003580145	sputum	negative	negative	negative	negative	Positive	Positive
#N/A	1371	Mbam1481	M abscessus	M148	GCA_000280655	sputum	negative	negative	negative	negative	Positive	Positive
#N/A	1194	Mbam1493	M abscessus	MAB_082312_2272	GCA_000523735	Homo sapiens	negative	negative	negative	negative	Positive	Positive
#N/A	1279	Mbam1494	M abscessus	MAB_091912_2446	GCA_000500185	sputum	negative	negative	negative	negative	Positive	Positive
#N/A	1236	Mbam1504	M abscessus	PAP006	GCA_001213185	sputum	negative	negative	negative	negative	Positive	Positive
#N/A	1232	Mbam1505	M abscessus	PAP007	GCA_001214025	sputum	negative	negative	negative	negative	Positive	Positive
#N/A	1219	Mbam1507	M abscessus	PAP009	GCA_001213065	sputum	negative	negative	negative	negative	Positive	Positive
#N/A	1233	Mbam1514	M abscessus	PAP016	GCA_001213545	sputum	negative	negative	negative	negative	Positive	Positive
#N/A	1220	Mbam1515	M abscessus	PAP017	GCA_001213125	sputum	negative	negative	negative	negative	Positive	Positive
#N/A	1262	Mbam1516	M abscessus	PAP018	GCA_001212825	sputum	negative	negative	negative	negative	Positive	Positive
#N/A	1065	Mbam1520	M abscessus	PAP023	GCA_001212965	sputum	negative	negative	negative	negative	Positive	Positive
#N/A	1230	Mbam1521	M abscessus	PAP024	GCA_001213325	sputum	negative	negative	negative	negative	Positive	Positive
#N/A	1221	Mbam1522	M abscessus	PAP025	GCA_001213805	sputum	negative	negative	negative	negative	Positive	Positive
#N/A	1228	Mbam1523	M abscessus	PAP026	GCA_001214125	sputum	negative	negative	negative	negative	Positive	Positive
#N/A	1222	Mbam1524	M abscessus	PAP027	GCA_001213245	sputum	negative	negative	negative	negative	Positive	Positive
#N/A	1259	Mbam1525	M abscessus	PAP028	GCA_001213905	sputum	negative	negative	negative	negative	Positive	Positive
#N/A	1234	Mbam1528	M abscessus	PAP031	GCA_001213765	sputum	negative	negative	negative	negative	Positive	Positive
#N/A	1223	Mbam1531	M abscessus	PAP034	GCA_001213145	sputum	negative	negative	negative	negative	Positive	Positive
#N/A	1229	Mbam1533	M abscessus	PAP037	GCA_001213585	sputum	negative	negative	negative	negative	Positive	Positive
#N/A	1074	Mbam1535	M abscessus	PAP039	GCA_001213405	sputum	negative	negative	negative	negative	Positive	Positive
#N/A	1382	Mbam1539	M abscessus	PAP043	GCA_001213025	sputum	negative	negative	negative	negative	Positive	Positive
#N/A	1231	Mbam1541	M abscessus	PAP045	GCA_001213865	sputum	negative	negative	negative	negative	Positive	Positive
#N/A	1079	Mbam1544	M abscessus	PAP048	GCA_001213625	sputum	negative	negative	negative	negative	Positive	Positive
#N/A	1129	Mbam1545	M abscessus	PAP049	GCA_001214165	sputum	negative	negative	negative	negative	Positive	Positive
#N/A	1269	Mbam1546	M abscessus	PAP050	GCA_001213465	sputum	negative	negative	negative	negative	Positive	Positive

#N/A	1260	Mbam1547	M abscessus	PAP051	GCA_001214565	sputum	negative	negative	negative	negative	Positive	Positive
#N/A	1264	Mbam1548	M abscessus	PAP052	GCA_001214205	sputum	negative	negative	negative	negative	Positive	Positive
#N/A	1120	Mbam1550	M abscessus	PAP055	GCA_001215245	sputum	negative	negative	negative	negative	Positive	Positive
#N/A	1113	Mbam1551	M abscessus	PAP057	GCA_001215485	sputum	negative	negative	negative	negative	Positive	Positive
#N/A	1112	Mbam1552	M abscessus	PAP058	GCA_001213425	sputum	negative	negative	negative	negative	Positive	Positive
#N/A	1066	Mbam1553	M abscessus	PAP059	GCA_001214005	sputum	negative	negative	negative	negative	Positive	Positive
#N/A	1384	Mbam1562	M abscessus	PAP070	GCA_001214145	sputum	negative	negative	negative	negative	Positive	Positive
#N/A	1075	Mbam1564	M abscessus	PAP072	GCA_001214645	sputum	negative	negative	negative	negative	Positive	Positive
#N/A	1055	Mbam1565	M abscessus	PAP073	GCA_001216825	sputum	negative	negative	negative	negative	Positive	Positive
#N/A	1078	Mbam1566	M abscessus	PAP074	GCA_001217165	sputum	negative	negative	negative	negative	Positive	Positive
#N/A	1076	Mbam1570	M abscessus	PAP078	GCA_001214725	sputum	negative	negative	negative	negative	Positive	Positive
#N/A	1071	Mbam1572	M abscessus	PAP080	GCA_001215325	sputum	negative	negative	negative	negative	Positive	Positive
#N/A	1077	Mbam1573	M abscessus	PAP081	GCA_001217225	sputum	negative	negative	negative	negative	Positive	Positive
#N/A	1063	Mbam1576	M abscessus	PAP084	GCA_001214465	sputum	negative	negative	negative	negative	Positive	Positive
#N/A	1067	Mbam1578	M abscessus	PAP086	GCA_001215645	sputum	negative	negative	negative	negative	Positive	Positive
#N/A	1070	Mbam1579	M abscessus	PAP087	GCA_001216005	sputum	negative	negative	negative	negative	Positive	Positive
#N/A	1370	Mbam1580	M abscessus	PAP088	GCA_001216925	sputum	negative	negative	negative	negative	Positive	Positive
#N/A	1224	Mbam1581	M abscessus	PAP089	GCA_001215065	sputum	negative	negative	negative	negative	Positive	Positive
#N/A	1123	Mbam1583	M abscessus	PAP091	GCA_001214285	sputum	negative	negative	negative	negative	Positive	Positive
#N/A	1056	Mbam1584	M abscessus	PAP092	GCA_001214305	sputum	negative	negative	negative	negative	Positive	Positive
#N/A	1227	Mbam1585	M abscessus	PAP093	GCA_001214405	sputum	negative	negative	negative	negative	Positive	Positive
#N/A	1086	Mbam1586	M abscessus	PAP094	GCA_001214885	sputum	negative	negative	negative	negative	Positive	Positive
#N/A	1131	Mbam1587	M abscessus	PAP095	GCA_001213365	sputum	negative	negative	negative	negative	Positive	Positive
#N/A	1057	Mbam1588	M abscessus	PAP096	GCA_001215905	sputum	negative	negative	negative	negative	Positive	Positive
#N/A	1263	Mbam1589	M abscessus	PAP097	GCA_001212945	sputum	negative	negative	negative	negative	Positive	Positive
#N/A	1225	Mbam1590	M abscessus	PAP098	GCA_001217365	sputum	negative	negative	negative	negative	Positive	Positive
#N/A	1130	Mbam1592	M abscessus	PAP100	GCA_001215345	sputum	negative	negative	negative	negative	Positive	Positive
#N/A	1266	Mbam1593	M abscessus	PAP101	GCA_001216185	sputum	negative	negative	negative	negative	Positive	Positive
#N/A	1126	Mbam1595	M abscessus	PAP103	GCA_001216225	sputum	negative	negative	negative	negative	Positive	Positive
#N/A	1155	Mbam1596	M abscessus	PAP104	GCA_001216625	sputum	negative	negative	negative	negative	Positive	Positive
#N/A	1132	Mbam1597	M abscessus	PAP105	GCA_001217405	sputum	negative	negative	negative	negative	Positive	Positive
#N/A	1116	Mbam1599	M abscessus	PAP107	GCA_001216665	sputum	negative	negative	negative	negative	Positive	Positive
#N/A	1058	Mbam1600	M abscessus	PAP108	GCA_001216365	sputum	negative	negative	negative	negative	Positive	Positive
#N/A	1117	Mbam1601	M abscessus	PAP109	GCA_001215285	sputum	negative	negative	negative	negative	Positive	Positive
#N/A	1267	Mbam1602	M abscessus	PAP110	GCA_001214445	sputum	negative	negative	negative	negative	Positive	Positive
#N/A	1122	Mbam1603	M abscessus	PAP111	GCA_001216405	sputum	negative	negative	negative	negative	Positive	Positive
#N/A	1135	Mbam1604	M abscessus	PAP112	GCA_001216865	sputum	negative	negative	negative	negative	Positive	Positive
#N/A	1134	Mbam1606	M abscessus	PAP114	GCA_001217285	sputum	negative	negative	negative	negative	Positive	Positive
#N/A	1115	Mbam1608	M abscessus	PAP116	GCA_001214525	sputum	negative	negative	negative	negative	Positive	Positive
#N/A	1491	Mbam1609	M abscessus	PAP117	GCA_001216165	sputum	negative	negative	negative	negative	Positive	Positive
#N/A	1121	Mbam1610	M abscessus	PAP118	GCA_001216725	sputum	negative	negative	negative	negative	Positive	Positive
#N/A	1119	Mbam1611	M abscessus	PAP119	GCA_001214825	sputum	negative	negative	negative	negative	Positive	Positive
#N/A	1226	Mbam1612	M abscessus	PAP120	GCA_001216905	sputum	negative	negative	negative	negative	Positive	Positive
#N/A	1265	Mbam1613	M abscessus	PAP121	GCA_001216885	sputum	negative	negative	negative	negative	Positive	Positive
#N/A	1268	Mbam1614	M abscessus	PAP122	GCA_001214665	sputum	negative	negative	negative	negative	Positive	Positive
#N/A	1059	Mbam1615	M abscessus	PAP123	GCA_001215025	sputum	negative	negative	negative	negative	Positive	Positive
#N/A	1118	Mbam1616	M abscessus	PAP124	GCA_001214505	sputum	negative	negative	negative	negative	Positive	Positive
#N/A	1111	Mbam1617	M abscessus	PAP125	GCA_001214265	sputum	negative	negative	negative	negative	Positive	Positive
#N/A	1114	Mbam1618	M abscessus	PAP126	GCA_001215305	sputum	negative	negative	negative	negative	Positive	Positive
#N/A	1125	Mbam1620	M abscessus	PAP128	GCA_001214845	sputum	negative	negative	negative	negative	Positive	Positive
#N/A	1258	Mbam1621	M abscessus	PAP129	GCA_001217325	sputum	negative	negative	negative	negative	Positive	Positive
#N/A	1261	Mbam1626	M abscessus	PAP134	GCA_001217465	sputum	negative	negative	negative	negative	Positive	Positive
#N/A	1235	Mbam1627	M abscessus	PAP135	GCA_001215205	sputum	negative	negative	negative	negative	Positive	Positive

#N/A	1060	Mbam1628	M abscessus	PAP136	GCA_001216805	sputum	negative	negative	negative	negative	Positive	Positive
#N/A	1061	Mbam1629	M abscessus	PAP137	GCA_001216045	sputum	negative	negative	negative	negative	Positive	Positive
#N/A	1080	Mbam1633	M abscessus	PAP141	GCA_001214685	sputum	negative	negative	negative	negative	Positive	Positive
#N/A	1073	Mbam1635	M abscessus	PAP143	GCA_001214345	sputum	negative	negative	negative	negative	Positive	Positive
#N/A	1068	Mbam1636	M abscessus	PAP144	GCA_001217105	sputum	negative	negative	negative	negative	Positive	Positive
#N/A	1062	Mbam1637	M abscessus	PAP145	GCA_001214805	sputum	negative	negative	negative	negative	Positive	Positive
#N/A	1276	Mbam1638	M abscessus	PAP146	GCA_001215605	sputum	negative	negative	negative	negative	Positive	Positive
#N/A	1156	Mbam1639	M abscessus	PAP147	GCA_001217485	sputum	negative	negative	negative	negative	Positive	Positive
#N/A	1275	Mbam1640	M abscessus	PAP148	GCA_001216245	sputum	negative	negative	negative	negative	Positive	Positive
#N/A	1278	Mbam1641	M abscessus	PAP149	GCA_001214945	sputum	negative	negative	negative	negative	Positive	Positive
#N/A	1133	Mbam1642	M abscessus	PAP150	GCA_001215225	sputum	negative	negative	negative	negative	Positive	Positive
#N/A	1286	Mbam1643	M abscessus	PAP151	GCA_001214905	sputum	negative	negative	negative	negative	Positive	Positive
#N/A	1287	Mbam1644	M abscessus	PAP152	GCA_001216105	sputum	negative	negative	negative	negative	Positive	Positive
#N/A	1282	Mbam1645	M abscessus	PAP154	GCA_001215705	sputum	negative	negative	negative	negative	Positive	Positive
#N/A	1283	Mbam1646	M abscessus	PAP155	GCA_001217005	sputum	negative	negative	negative	negative	Positive	Positive
#N/A	1289	Mbam1647	M abscessus	PAP156	GCA_001215125	sputum	negative	negative	negative	negative	Positive	Positive
#N/A	1280	Mbam1648	M abscessus	PAP157	GCA_001215745	sputum	negative	negative	negative	negative	Positive	Positive
#N/A	1492	Mbam1649	M abscessus	PAP158	GCA_001217065	sputum	negative	negative	negative	negative	Positive	Positive
#N/A	1124	Mbam1650	M abscessus	PAP159	GCA_001216685	sputum	negative	negative	negative	negative	Positive	Positive
#N/A	1277	Mbam1654	M abscessus	PAP164	GCA_001216525	sputum	negative	negative	negative	negative	Positive	Positive
#N/A	1284	Mbam1655	M abscessus	PAP167	GCA_001216325	sputum	negative	negative	negative	negative	Positive	Positive
#N/A	1285	Mbam1656	M abscessus	PAP168	GCA_001214385	sputum	negative	negative	negative	negative	Positive	Positive
#N/A	1290	Mbam1657	M abscessus	PAP169	GCA_001215825	sputum	negative	negative	negative	negative	Positive	Positive
#N/A	1281	Mbam1659	M abscessus	PAP171	GCA_001214605	sputum	negative	negative	negative	negative	Positive	Positive
#N/A	#N/A	Mche1664	M chelonae	203	GCA_001853695	python	negative	negative	negative	negative	negative	negative
#N/A	#N/A	Mche1665	M chelonae	1557	GCA_001853585	Not available	negative	negative	negative	negative	negative	negative
#N/A	#N/A	Mche1666	M chelonae	1558	GCA_001853805	sputum	negative	negative	negative	negative	negative	negative
#N/A	#N/A	Mche1667	M chelonae	15510	GCA_001853845	soft tissue (calf)	negative	negative	negative	negative	negative	negative
#N/A	#N/A	Mche1668	M chelonae	15511	GCA_001853865	soft tissue (foot)	negative	negative	negative	negative	negative	negative
#N/A	#N/A	Mche1669	M chelonae	15512	GCA_001853925	sputum	negative	negative	negative	negative	negative	negative
#N/A	#N/A	Mche1670	M chelonae	15513	GCA_001853885	soft tissue (axilla)	negative	negative	negative	negative	negative	negative
#N/A	#N/A	Mche1671	M chelonae	15514	GCA_001853965	eye	negative	negative	negative	negative	negative	negative
#N/A	#N/A	Mche1672	M chelonae	15515	GCA_001853945	knee (synovial fluid)	negative	negative	negative	negative	negative	negative
#N/A	#N/A	Mche1673	M chelonae	15517	GCA_001853985	soft tissue (finger)	negative	negative	negative	negative	negative	negative
#N/A	#N/A	Mche1674	M chelonae	15519	GCA_001853895	soft tissue (skin)	negative	negative	negative	negative	negative	negative
#N/A	#N/A	Mche1675	M chelonae	15520	GCA_001854015	soft tissue (leg)	negative	negative	negative	negative	negative	negative
#N/A	#N/A	Mche1676	M chelonae	24998	GCA_001853535	seahorse, ovary	negative	negative	negative	negative	negative	negative
#N/A	#N/A	Mche1677	M chelonae	29191	GCA_001853745	seadragon, soft tissue	negative	negative	negative	negative	negative	negative
#N/A	#N/A	Mche1678	M chelonae	29192	GCA_001853715	seadragon, liver mesentery	negative	negative	negative	negative	negative	negative
#N/A	#N/A	Mche1679	M chelonae	35599	GCA_001853605	seahorse, tail	negative	negative	negative	negative	negative	negative
#N/A	#N/A	Mche1680	M chelonae	35600	GCA_001853615	seahorse, ovary	negative	negative	negative	negative	negative	negative
#N/A	#N/A	Mche1681	M chelonae	37256	GCA_001942575	Cichlid, spleen	negative	negative	negative	negative	negative	negative
#N/A	#N/A	Mche1682	M chelonae	42895	GCA_001853545	seahorse, skeletal muscle	negative	negative	negative	negative	negative	negative
#N/A	#N/A	Mche1683	M chelonae	96-1705	GCA_002013595	Foot biopsy	negative	negative	negative	negative	negative	negative
#N/A	#N/A	Mche1684	M chelonae	96-1717	GCA_002013725	Tissue from hand	negative	negative	negative	negative	negative	negative
#N/A	#N/A	Mche1685	M chelonae	96-1720	GCA_002013305	Leg abscess	negative	negative	negative	negative	negative	negative
#N/A	#N/A	Mche1686	M chelonae	96-1724	GCA_002013635	Leg abscess	negative	negative	negative	negative	negative	negative
#N/A	#N/A	Mche1687	M chelonae	96-1728	GCA_002013315	liver tissue	negative	negative	negative	negative	negative	negative
#N/A	#N/A	Mche1688	M chelonae	ATCC-19237	GCA_001942555	gastric lavage	negative	negative	negative	negative	negative	negative
#N/A	#N/A	Mche1689	M chelonae	ATCC-35752	GCA_000971805	Not available	negative	negative	negative	negative	negative	negative
#N/A	#N/A	Mche1690	M chelonae	B17_S14	GCA_001853775	aquarium	negative	negative	negative	negative	negative	negative
#N/A	#N/A	Mche1691	M chelonae	B20_S13	GCA_001853665	aquarium	negative	negative	negative	negative	negative	negative
#N/A	#N/A	Mche1692	M chelonae	B3_S15	GCA_001853685	Not available	negative	negative	negative	negative	negative	negative

#N/A	#N/A	Mche1693	M chelonae	CCUG-47445	GCA_001632805	Not available	negative	negative	negative	negative	negative	negative
#N/A	#N/A	Mche1694	M chelonae	D16Q24	GCA_002013815	Tap water	negative	negative	negative	negative	negative	negative
#N/A	#N/A	Mche1695	M chelonae	D16R10	GCA_002013675	surface water	negative	negative	negative	negative	negative	negative
#N/A	#N/A	Mche1696	M chelonae	D16R14	GCA_002013825	Tap water	negative	negative	negative	negative	negative	negative
#N/A	#N/A	Mche1697	M chelonae	D16R19	GCA_002013515	surface water	negative	negative	negative	negative	negative	negative
#N/A	#N/A	Mche1698	M chelonae	D16R2	GCA_002013455	Tap water	negative	negative	negative	negative	negative	negative
#N/A	#N/A	Mche1699	M chelonae	D16R20	GCA_002013535	surface water	negative	negative	negative	negative	negative	negative
#N/A	#N/A	Mche1700	M chelonae	D16R7	GCA_002013855	Tap water	negative	negative	negative	negative	negative	negative
#N/A	#N/A	Mche1701	M chelonae	D16R9	GCA_002013485	Tap water	negative	negative	negative	negative	negative	negative
#N/A	#N/A	Mche1703	M chelonae	Trumpetfish	GCA_001854035	trumpetfish	negative	negative	negative	negative	negative	negative
#N/A	#N/A	Mfra1704	M franklinii	1559	GCA_001853795	soft tissue (nasal)	negative	negative	negative	negative	negative	negative
#N/A	#N/A	Mfra1705	M franklinii	CV002	GCA_002013895	skin lesion	negative	negative	negative	negative	negative	negative
#N/A	#N/A	Mfra1706	M franklinii	D16Q19	GCA_002013715	Water work	negative	negative	negative	negative	negative	negative
#N/A	#N/A	Mfra1707	M franklinii	D16R27	GCA_002013295	Tap water	negative	negative	negative	negative	negative	negative
#N/A	#N/A	Mfra1708	M franklinii	DSM-45524	GCA_002086225	Not available	negative	negative	negative	negative	negative	negative
#N/A	#N/A	Mimm1709	M immunogenum	ATCC-700505	GCA_002101665	metalworking fluids	negative	negative	negative	negative	negative	negative
#N/A	#N/A	Mimm1710	M immunogenum	CCUG-47286	GCA_001605725	Not available	negative	negative	negative	negative	negative	negative
#N/A	#N/A	Mimm1711	M immunogenum	CD11-6	GCA_001655155	Duodenal mucosa Celiac	negative	negative	negative	negative	negative	negative
#N/A	#N/A	Mimm1712	M immunogenum	FLAC016	GCA_001677135	Homo sapiens	negative	negative	negative	negative	negative	negative
#N/A	#N/A	Mimm1713	M immunogenum	H008	GCA_001296275	drinking water	negative	negative	negative	negative	negative	negative
#N/A	#N/A	Mimm1714	M immunogenum	H060	GCA_001296265	drinking water	negative	negative	negative	negative	negative	negative
#N/A	#N/A	Mimm1715	M immunogenum	H068	GCA_001296245	drinking water	negative	negative	negative	negative	negative	negative
#N/A	#N/A	Mimm1716	M immunogenum	H074	GCA_001296255	drinking water	negative	negative	negative	negative	negative	negative
#N/A	#N/A	Mimm1717	M immunogenum	H088	GCA_001296345	drinking water	negative	negative	negative	negative	negative	negative
#N/A	#N/A	Mimm1718	M immunogenum	H097	GCA_001296415	drinking water	negative	negative	negative	negative	negative	negative
#N/A	#N/A	Mimm1719	M immunogenum	H106	GCA_001296385	drinking water	negative	negative	negative	negative	negative	negative
#N/A	#N/A	Mimm1720	M immunogenum	H113	GCA_001296405	drinking water	negative	negative	negative	negative	negative	negative
#N/A	#N/A	Mimm1721	M immunogenum	HXV	GCA_001296335	drinking water	negative	negative	negative	negative	negative	negative
#N/A	#N/A	Mimm1722	M immunogenum	HXXI	GCA_001296425	drinking water	negative	negative	negative	negative	negative	negative
#N/A	#N/A	Mimm1723	M immunogenum	MC-779	GCA_002013555	bronchoalveolar fluid	negative	negative	negative	negative	negative	negative
#N/A	#N/A	Mimm1724	M immunogenum	SMUC14	GCA_000878425	brain abscess	negative	negative	negative	negative	negative	negative
#N/A	#N/A	Msal1725	M salmoniphilum	D16Q15	GCA_002013645	Tap water	negative	negative	negative	negative	negative	negative
#N/A	#N/A	Msal1726	M salmoniphilum	SC	GCA_002013685	Chinook salmon	negative	negative	negative	negative	negative	negative
#N/A	#N/A	Msao1727	M saopaulense	8489	GCA_001853625	turtle, soft tissue	negative	negative	negative	negative	negative	negative
#N/A	#N/A	Msao1728	M saopaulense	8528	GCA_001853765	cow, aquarium	negative	negative	negative	negative	negative	negative
#N/A	#N/A	Msao1729	M saopaulense	CCUG-66554	GCA_002086715	Not available	negative	Positive	negative	negative	negative	negative

1. SNP: Order in the core genome SNP tree shown in Figure 2, from top to bottom. cgMLST: Order in the cgMLST tree shown in Figure S2, from top to bottom.
2. Assignment to MABC subspecies. Mbaa = *M. abscessus* subspecies *abscessus*, Mbab = *M. abscessus* subspecies *bolletii* and Mbam = *M. abscessus* subspecies *massiliense*.
3. Genus/species/subspecies description as used in the NCBI Genomes database.
4. Accession number to the genome assembly used in the analysis.
